# Establishment and Evaluation of a Parametric Population Pharmacokinetic Model Repository for Ganciclovir and Valganciclovir

**DOI:** 10.3390/pharmaceutics15071801

**Published:** 2023-06-23

**Authors:** Wenyu Yang, Wenyao Mak, Amanda Gwee, Meng Gu, Yue Wu, Yufei Shi, Qingfeng He, Xiaoqiang Xiang, Bing Han, Xiao Zhu

**Affiliations:** 1 Department of Clinical Pharmacy and Pharmacy Administration, School of Pharmacy, Fudan University, Shanghai 201203, China; 21211030109@m.fudan.edu.cn (W.Y.); makwenyao@gmail.com (W.M.); 20211030060@fudan.edu.cn (M.G.); yufei_shi@fudan.edu.cn (Y.S.); qf_he@fudan.edu.cn (Q.H.); xiangxq@fudan.edu.cn (X.X.); 2 Department of Pharmacy, Minhang Hospital, Fudan University, Shanghai 201199, China; 3 Department of General Medicine, Royal Children’s Hospital, Parkville, VIC 3052, Australia; amanda.gwee@rch.org.au; 4 Infectious Diseases Group, Murdoch Children’s Research Institute, Parkville, VIC 3052, Australia; 5 Department of Paediatrics, The University of Melbourne, Parkville, VIC 3010, Australia; 6 Department of Clinical Pharmacy, Shenzhen Children’s Hospital Affiliated to Shantou University Medical College, Shenzhen 518038, China; wuyue161@163.com

**Keywords:** ganciclovir, valganciclovir, population pharmacokinetics, model-informed precision dosing, model repository

## Abstract

Background: Ganciclovir and valganciclovir are used for prophylaxis and treatment of cytomegalovirus infection. However, there is great interindividual variability in ganciclovir’s pharmacokinetics (PK), highlighting the importance of individualized dosing. To facilitate model-informed precision dosing (MIPD), this study aimed to establish a parametric model repository of ganciclovir and valganciclovir by summarizing existing population pharmacokinetic information and analyzing the sources of variability. (2) Methods: A total of four databases were searched for published population PK models. We replicated these models, evaluated the impact of covariates on clearance, calculated the probability of target attainment for each model based on a predetermined dosing regimen, and developed an area under the concentration–time curve (AUC) calculator using maximum a posteriori Bayesian estimation. (3) Results: A total of 16 models, one- or two-compartment models, were included. The most significant covariates were body size (weight and body surface area) and renal function. The results show that 5 mg/kg/12 h of ganciclovir could make the AUC_0–24h_ within 40–80 mg·h/L for 50.03% pediatrics but cause AUC_0–24h_ exceeding the exposure thresholds for toxicity (120 mg·h/L) in 51.24% adults. (4) Conclusions: Dosing regimens of ganciclovir and valganciclovir should be adjusted according to body size and renal function. This model repository has a broad range of potential applications in MIPD.

## 1. Introduction

Ganciclovir (GCV) is often used for, but not limited to, the treatment or prophylaxis of cytomegalovirus (CMV) disease in immunocompromised patients, such as those with acquired immunodeficiency syndrome and iatrogenic immunosuppression associated with organ transplantation or chemotherapy of neoplastic disease [1]. GCV’s antiviral activity is mediated through its triphosphate, which inhibits viral DNA polymerase and slows DNA elongation [2]. Valganciclovir (VGCV) is an L-valine ester prodrug of GCV formulated as an oral solution or tablet to overcome the low bioavailability of oral GCV (~5%) [3]. VGCV has a higher oral bioavailability of approximately 60% [4]. Plasma protein binding of GCV is minimal, accounting for approximately 1 to 2% over the concentration range of 0.5–51 mg/L [5]. GCV clearance correlates well with renal function as over 90% is eliminated unchanged in urine [6].

Currently, there is no consensus on the pharmacokinetic–pharmacodynamic (PK–PD) endpoints for GCV due to a paucity of clinical studies examining efficacy targets in adults or pediatric populations. Both the trough concentration (C_trough_) and the area under the concentration–time curve (AUC) have been used for therapeutic drug monitoring (TDM). Recently, a 24 h AUC (AUC_0–24h_) above 50 mg·h/L has been suggested by Märtson et al. [7] for prophylaxis and 80–120 mg·h/L for treatment of CMV infection.

Several studies have shown that GCV exhibited high interindividual variability (IIV) in different populations [8,9,10,11]. As a result, the use of a one-size-fits-all dose for all patients could result in treatment failure. Individualized dosing and TDM play crucial roles in optimizing GCV efficacy and safety. Population pharmacokinetics (PPK) analyses can identify covariates that influence PK parameters and produce estimates of individual PK parameters through Bayesian forecasting to develop individualized therapy for GCV.

To achieve individualized dosing, a comprehensive understanding of the PK parameters and PPK models of GCV is necessary. However, to our knowledge, there are no published PPK model repositories. Therefore, the overarching aim of our work was to establish a parametric PPK model repository of GCV and VGCV. We would like to highlight that the goal of this study is not to prove that these models are comparable with each other, but rather to showcase the heterogeneity. Specifically, we attempted to (1) identify all published PPK models to the best of our capability through a comprehensive literature search, and to reorganize these models using standardized R codes; (2) assess the performance of different PPK models in this repository by comparing the concentration–time profiles and covariates effects; and (3) illustrate the utility of the model repository with two examples (i.e., Monte Carlo simulation for the probability of target attainment (PTA) and AUC calculator based on maximum a posteriori Bayesian estimation (MAP-BE)). By building this model repository and demonstrating the sources of variability, we aim to facilitate subsequent utilization of these models to improve precision dosing of GCV and VCGV.

## 2. Methods

### 2.1. Search Strategy

In an attempt to identify all parametric PPK models, a literature search was performed on the PubMed, Embase, Scopus and Web of Science databases from inception to 28 May 2023, according to the Preferred Reporting Items for Systematic Reviews and Meta-analyses (PRISMA) reporting guideline [12]. Search terms included those related to the drug of interest (ganciclovir, BW-759, valganciclovir, Cytovene, and Valcyte) and terms specific to PPK models included population pharmacokinetic, NONMEM, MONOLIX, and *nlmixr*. The latter terms were derived from a publication by Li et al. [13]. Two authors (W.Y. and M.G.) conducted the literature search and study selection independently using EndNote (version 20.0.0; Thomson Scientific, Box Hill, Victoria, Australia). A third senior investigator (Q.H.) was consulted to resolve any discrepancies between the two authors. The complete search strategies for each database, and inclusion and exclusion criteria are available in Supplementary Materials.

### 2.2. Information Extraction

We extracted the following information from the included studies: (1) study characteristics including subject demographics, sampling strategies, dosage regimens, and quantitative methods; (2) PPK modeling characteristics, including software and algorithms used, PK parameters and related formulas, between-subject variability (BSV, which was recorded as the coefficient of variation (CV), and %CV=$\sqrt{omega^{2}}*100\%$), residual unexplained variability (RUV), validation methods; and (3) covariate information, including the list of all covariates that were tested, selection criteria and the subset of covariates that had significant effects on the PK parameters.

### 2.3. Quality Control of the PPK Model Repository

Quality control (QC) procedures were undertaken to screen and rectify any issues related to the establishment of the model repository. We created 4 age-stratified cohorts of virtual patients (neonates, infants, children and adults), these typical virtual patients were designed to reflect the target population of each model as accurately as possible (See Table 1 for details). Although the *age* covariate had been converted into a categorical variable, this should not impede the validity of the virtual patient cohort as the representative values of each cohort were derived from the median of observed data. The corresponding steady-state concentration–time curves for each virtual patients were obtained by simulations (each simulation was performed on 1000 typical virtual patients) with rxode2 package (version 2.0.12) of R (version 4.2.2). The principle that guided the QC procedures was that, given the models were appropriate to describe the corresponding PPK characteristics, the simulated concentration–time curves of the same typical virtual patients generated by different models should be comparable. This was defined as the 95% confidential interval (CI) of the geometric mean of time to maximum concentration (T_max_), maximum concentration (C_max_) and other PK parameters of 1000 virtual patients in a study should fall within the range of 50–200% of the geometric mean of PK parameters of all the same virtual patients. When significant differences in the simulated concentration–time curves were detected (i.e., the C_max_ was outside of this range), potential model reproduction errors were first examined and excluded, and then the covariates included in the models were compared to identify possible causes that affected the PK behaviors.

The PK parameters used for similarity comparison, including C_max_, T_max_, and the half-life time (t_1/2_) were derived from non-compartment analysis (NCA) analysis of drug concentrations in virtual patients by using IQnca (Version 1.3.0) and Rmisc (Version 1.5.1) package.

All virtual patients received 5 mg/kg GCV by intravenous infusion over 1 h every 12 h (q12h). For VGCV, neonates, infants, and children received 10 mg/kg q12h while adult received 900 mg q12h. For pediatrics, their dosing regimens followed the commonly used dosing regimens of the included studies; for adults, the dosing regimen was based on the third international consensus guidelines on the management of cytomegalovirus in solid-organ transplantation [14]. The R codes of model repository establishment were provided in Supplementary Materials.

### 2.4. Effect of Covariates on Clearance Variation

Clearance (CL) is a crucial parameter for AUC, and AUC plays a central role in the individualized dosing of GCV. Thus, the comparison of the effects of different covariates on CL was necessary. To explore whether the covariate effect on CL was clinically meaningful and to understand between-study differences in covariate impacts on CL, we used a forest plot to comprehensively compare covariate effects across studies. Weight, estimated glomerular filtration rate (eGFR) and creatinine clearance (CLcr) were scaled to the same range and a uniform covariate value was set as the reference (refer to Table S1 for details). For other continuous covariates (serum creatinine, SCR and body surface area, BSA) that were only identified in one study, the minimum and maximum values of that covariate were used to calculate CL and the reference CL value was calculated using the median covariate value of each study.

For binary covariates, such as the presence of critical illness (defined as “1” for critically ill patients and “0” for others), the common condition would be treated as the reference (COV_i_ = 0). The uncommon condition would be treated as the test (COV_i_ = 1). CL_i_ = CL_common_ + CL_diff_ * COV_i_. The range of CL_i_ would be [CL_common_, CL_commpn_ + CL_diff_] (if CL_diff_ > 0), or [CL_common_ + CL_diff_, CL_common_] (if CL_diff_ < 0).

Then, the effect range of identified covariate on CL was calculated by the following formula:

(1) $$\mathrm{Covariate}\;\mathrm{effect}=\frac{\mathrm{The}\;\mathrm{minimum}\;\mathrm{CL}\;\mathrm{or}\;\mathrm{the}\;\mathrm{maximum}\;\mathrm{CL}}{\mathrm{Reference}\;\mathrm{CL}}\times 100\%$$

We considered covariates effects outside of the 80–125% boundary as clinically significant, based on the standard used in bioequivalence studies [13,15]. Detailed R codes can be found in the Supplementary Materials.

### 2.5. Application of the PPK Model Repository

In order to demonstrate the practical application of the model repository in MIPD, we will provide two illustrative examples.

#### 2.5.1. Monte Carlo Simulation for the Probability of Target Attainment

We calculated the PTA of simulated concentration–time curves to evaluate the commonly used dosing regimens of GCV and VGCV. The trapezoidal method was used to calculate the steady-state AUC_0–24h_ for 1000 virtual patients. The prophylaxis target used was 40–80 mg·h/L [16], while AUC_0–24h_ of 80–120 mg·h/L was considered treatment target [7]. AUC exceeding 120 mg·h/L posed toxic risk.

#### 2.5.2. AUC Calculator Based on Maximum a Posteriori Method

When using GCV and VGCV in a real-world setting, clinicians often need to calculate the AUC to determine whether the patient has achieved the appropriate level of exposure. To highlight the model repository’s convenience in MIPD, we developed an AUC_0–24h_ calculator based on the *maximum a posteriori*-Bayesian Estimation (MAP-BE) using R shiny (version 1.7.4). Four main components made up the core function of the calculator: (1) PPK model parameter information; (2) model defined by rxode2 package; (3) the objective function; (4) function used for MAP estimation [17].

## 3. Results

### 3.1. Identification of the Included Studies

**Figure 1:**
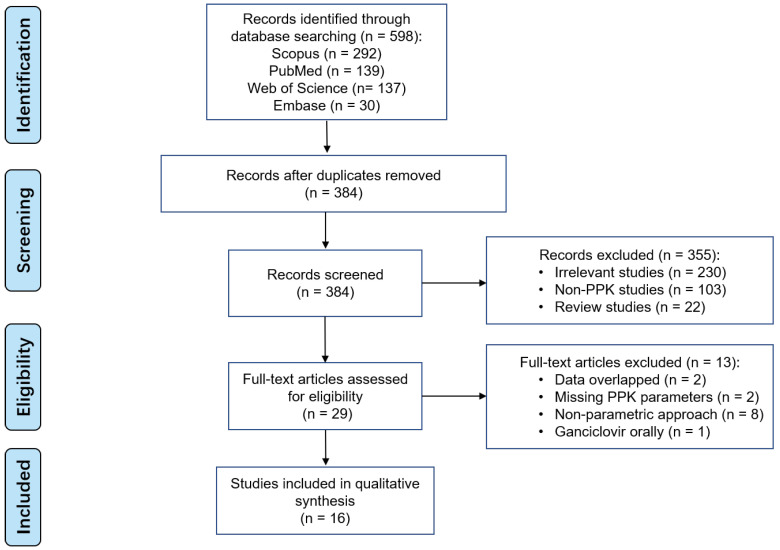
PRISMA flow diagram for the identification of ganciclovir parametric PPK studies.

598 studies were found in the initial search across various databases. A total of 16 studies were included in the final analysis, as shown in Figure 1. No additional records were identified from other sources.

### 3.2. Overview of Included PPK Models for GCV and VGCV

#### 3.2.1. Study and PPK Model Characteristics

All the included studies were published between 1995 and 2023. The characteristics of each study are summarized in Table 2. Twelve of the sixteen studies were prospective [8,11,18,19,20,21,22,23,24,25,26,27] and four studies were retrospective [9,10,28,29]. The number of subjects ranged from 8 to 105, with only two studies with less than 10 subjects [8,24]. Five studies utilized both intensive and sparse sampling strategies [8,18,20,22,27], six studies took samples by intensive sampling strategies only [9,21,23,24,26,28], one study used a sparse sampling strategy [25] and the remaining four studies did not report this information [10,11,19,29]. Seven studies were conducted only in pediatrics [8,9,11,19,20,23,29], two studies included both children and adults [25,28], and seven studies only enrolled adults [10,18,21,22,24,26,27]. Seven papers studied both GCV and VGCV [9,11,20,21,22,23,27], four were of VGCV alone [8,25,26,28], and five were of GCV [10,18,19,24,29]. The most common GCV dosing regimen was 5 mg/kg q12h [9,11,21,22,23,24,29]. For VGCV, a dose of 10 mg/kg q12h was used in children for pre-emptive therapy [8,9,23], while a dose of 900 mg q12h was prescribed in adults for treatment [21,22,25].

Modeling strategies and final pharmacokinetic parameters of the included studies are summarized in Table 3. Most PPK models were analyzed with NONMEM, with only two that used Monolix or Phoenix NLME [11,29]. First-order conditional estimation with interaction (FOCE-I) was the most commonly used algorithm. Eleven studies described GCV PPK as a two-compartment model [9,11,18,21,22,23,24,25,26,27,28] while another five studies concluded with a one-compartment model [8,10,19,20,29], four of which were conducted in the pediatric populations with sparse sampling [8,19,20,29]. Interestingly, our results suggested that the one-compartment model was more likely to be chosen when the sampling was sparse. In addition, studies that were conducted on critically ill patients were more likely to use the one-compartment model. Absorption of VGCV in all related studies was described as a first-order absorption process. It is important to note that the pharmacokinetics parameters reported in this study were given in terms of GCV alone. Two studies (Lalagkas et al. [27] and Caldés et al. [21], Table 3) had converted VGCV dose based on the conversion equation $Dose_{GCV}=Dose_{VGCV}\times 0.72$ to arrive at the appropriate GCV dosage for inclusion in the model building process. The ratio of 0.72 was based on the difference in molecular weight for GCV and VGCV. The remaining VGCV studies that reported bioavailability did not perform such a conversion.

BSV was described by an exponential model in all the included studies. RUV was described by a proportional model in ten studies [8,10,11,18,19,22,24,25,28,29], exponential model in three studies [20,23,26], additive model in one study [9], and combined proportional and additive model in two study. Two studies reported interoccasion variability (IOV). Facchin et al. [28] found an IOV in CL of 14.4%, 77.2% in peripheral volume of distribution (Vp), and 111.4% in absorption rate constant (ka). Perrottet et al. [22] reported an IOV in CL of 12%. Two studies published before 2000 did not report model evaluation results [18,19], but after careful inspection, the performance of these two models was comparable to others. Other studies were evaluated by internal validation; three of them also underwent external validation [11,26,27]. GOF, VPC, normalized prediction distribution error (NPDE) and bootstraps were often used as internal validation methods.

**Table 3:** Model strategies and final pharmacokinetic parameters of the included studies.

Study (Publication Year)	Software/ Algorithm	Fixed Effect Parameters		Between-Subject Variability (%)	Residual Unexplained Variability	Internal Validation	External Validation (N = No. of Subjects)	Model Application	Simulation Target
Lalagkas et al. (2023) * [27]	NONMEM / FOCE-I	CL (L/h) Vc (L) Q (L/h) Vp (L) Ka (1/h) F Tlag (h)	=6.93 × (CKD-EPI/55)^0.817^ × (BW/70)^0.75^ = 43.1 × (BW/70) = 9.23 × (BW/70)^0.75^ = 219 × (BW/70) = 0.766 = 0.699 = 0.331	29.9 36.1 / 103.4 45.7 16.6 /	28.2% (proportional error) 0.237 mg/L (additive error)	GOF pcVPC NPDE Bootstrap	N = 22	evaluate and design dosing regime	AUC_0–24h_: 40–50 mg/L·h
Nguyen et al. (2021) [11]	Monolix / SAEM	CL (L/h) V_c_ (L) Q (L/h) V_p_ (L) K_a_ (1/h) F	=2.55 × (BW/11.7)^0.75^ × (eGFR/167)^0.763^× 0.806^critically ill ^ = 5.96 × (BW/11.7) = 0.222 × (BW/11.7)^0.75 ^ = 1.29 × (BW/11.7) = 0.506 = 0.438	48.6 46.9 / / / /	47.7% (proportional error)	GOF NPDE pcVPC	N = 35	design dosing regime	preventive AUC_0–24h_: 40–80 mg·h/L curative AUC_0–24h_: 80–120 mg·h/L
Franck et al. (2021) [9]	NONMEM / NR	CL (L/h) V_c_ (L) Q (L/h) V_p_ (L) Tlag (h) K_a_ (1/h) F	=6.9 × (BW/26.7)^0.75^ × (CrCL/149.8)^0.88^ = 9.7 × (BW/26.7) = 10.9 = 7.6 × (BW/26.7) = 0.33 = 0.73 = 0.43	66.3 76.8 / / / 83.7 55.7	0.98 mg/L (additive error)	GOF pcVPC NPDE Bootstrap	NR	design dosing regime	AUC_0–24h_: 40–60 mg·h/L
Chen et al. (2021) [26]	NONMEM / FOCE	CL/F (L/h) V_c_/F (L) Q/F (L/h) V_p_/F (L) K_a_ (1/h) Tlag (h)	=7.09 × (1 + CLcr/68.3 × 1.08) = 10.8 = 3.96 = 174 = 0.23 = 0.93	27.2 153 63.1 107 / /	42.9% (exponential error)	GOF VPC Bootstrap	N = 30	LSS design dosing regime	AUC_0–24h_: 40–50 mg·h/L
Li et al. (2021) [29]	Phoenix NLME / FOCE-LB	CL (L/h) V_c_ (L)	=5.23 × KF^0.92^ × (BW/12.0)^1.02^ = 11.35 × (BW/12.0)^0.80^	12.9 65.8	8.23% (proportional error)	GOF VPC Bootstrap NPDE	NR	design dosing regime	AUC_0–24h_: 40–50 mg·h/L
Krens et al. (2020) [10]	NONMEM / FOCE-I	CL (L/h) V_c_ (L)	=2.3 × (CKD-EPI/65)^0.71 ^ = 42	47.0 80.0	43% (proportional error)	GOF VPC Bootstrap	NR	evaluate dosing regime	C_trough_ > 1.5 mg/L
Facchin et al. (2019) [28]	NONMEM / FOCE-I	CL/F (L/h) V_c_/F (L) Q/F (L/h) V_p_/F (L) K_a_ (1/h) Tlag (h)	=9.07 × (SCR/72.5)^-0.768^ × BSA^1.31^ × 1.15^GENDER ^ = 45 × BSA^1.28^ × 1.14^GENDER^ = 1.46 = 18.5 = 6.96 = 0.86	16.0 9.3 / 54.6 59.2 /	23.5% (proportional error)	GOF pcVPC NPDE Bootstrap	NR	design dosing regime	AUC_ss-12h_ AUC_ss-24h_
Horvatits et al. (2014) [24]	NONMEM / FOCE	CL (L/h) V_c_ (L) Q (L/h) V_p_ (L)	=2.2 = 32.4 = 16.8 = 33.5	61.5 33.6 34.7 60.6	7.22% (proportional error)	GOF VPC	NR	design dosing regime	AUC_0–24h_: 50 mg·h/L C_trough_ > 2 mg/L
Vezina et al. (2014) [25]	NONMEM / FOCE-I	CL/F (L/h) V_c_/F (L) Q/F (L/h) V_p_/F (L) K_a_ (1/h) Tlag (h)	=14.5 × ((CLcr/60) × (70/BW))^0.492^ × (BW/70)^0.75 ^ = 87.5 × (BW/70) = 4.80 × (BW/70)^0.75^ = 42.6 × (BW/70) = 3 = 0.5	33.5 / / / / /	32.7% (proportional error)	GOF VPC NPDE Bootstrap	NR	evaluate dosing regime	AUC_0-ꝏ_: 40–50 mg·h/L
Vezina et al. (2010) [8]	NONMEM / FOCE-I	CL/F (L/h) V_c_/F (L) K_a_ (1/h)	=7.33 = 35.1 = 0.85	36.3 41.4 74.3	33.5% (proportional error)	GOF	NR	analysis of efficacy	AUC_0–ꝏ_
Caldés et al. (2009) * [21]	NONMEM / FOCE-I	CL (L/h) V_c_ (L) Q (L/h) V_p_ (L) K_a_ (1/h) F Tlag (h)	=7.49 × (CLcr/57) = 31.90 = 10.2 = 32.0 = 0.895 = 0.825 = 0.382	32.7 47.6 / / 68.1 22.1 /	14.3% (proportional error) 0.465 μg/mL (additive error)	GOF Bootstrap	NR	evaluate and design dosing regime	AUC_0–24h_: 45 mg·h/L
Perrottet et al. (2009) [22]	NONMEM / FOCE	CL (L/h) V_c_ (L) Q (L/h) V_p_ (L) F K_a_ (1/h)	=θ_GraftType_ × GFR_MDRD_ × 1.21^sex ^ = 24 × (BW/70) × 0.78^sex^ = 4.1 = 22 = 0.6 = 0.56	26 20 / / / /	21% (proportional error)	GOF	NR	analysis of prophylactic efficacy and tolerability	AUC C_trough_
Zhao et al. (2009) [23]	NONMEM / FOCE	CL/F (L/h) V_c_/F (L) V_p_/F (L) Q/F (L/h) K_a_ (1/h) Tlag (h)	=8.04 × (CLcr/89)^2.93^ + 3.62 × (BW/28) = 5.2 = 30.7 = 3.97 = 0.369 = 0.743	23.83 58.22 / / 32.25 /	20.93% (exponential error)	GOF VPC Bootstrap	NR	design dosing regime	AUC_0–24h_: 45 mg·h/L C_trough_: 0.5 mg/L, 1 mg/L
Acosta et al. (2007) [20]	NONMEM / FOCE-I	CL (L/h) V (L) K_a_ (1/h) F	=0.146 × BW^1.68 ^ = 1.15 × BW = 0.591 = 0.536	28.4 / / 12.4	45.4% (exponential error)	GOF	NR	evaluate dosing regime	AUC_0–12h_: 27 mg·h/L
Zhou et al. (1996) [19]	NONMEM / NR	CL (L/h) V_c_ (L)	=0.262 + (0.00271 × ASCC) = 0.627 + (0.437 × BW)	35.4 COV = 28.5 30.1	8.46% (proportional error)	NR	NR	evaluate effect of covariates	concentration–time profiles
Yuen et al. (1995) [18]	NONMEM / NR	CL (L/h) V_c_ (L) V_p_ (L) Q (L/h)	=0.382 + 0.168 × BW × CLcr/100 × (1-T) × (1-CMV) = 0.381 × BW = 0.511 × BW = 13.4	47.5 27.5 / /	36.1% (proportional error)	NR	NR	evaluate effect of HIV	concentration–time profiles

*: These two models converted the VGCV doses to their equivalent GCV content multiplying the VGCV dose by 0.72 (the ratio between the molecular weights of GCV and VGCV). ASCC: approximated creatinine clearance from serum (mL/min/1.73 m^2^); BSA: body surface area (m^2^); BW: body weight (kg); CKD-EPI: the estimated glomerular filtration rate calculated by the Chronic Kidney Disease Epidemiology Collaboration (CKD-EPI) equation; CL: clearance; CLcr: creatinine clearance (mL/min); critically ill: 1 for critically ill patients and 0 for others; CMV: CMV = 0 for CMV-shedding patients and 0.41 for patients with CMV retinitis; COV: covariance between CL and V_c_; CrCL: creatinine clearance (mL/min/1.73 m^2^); C_trough_: trough concentration; eGFR: the estimated glomerular filtration rate (mL/min/1.73 m^2^); F: bioavailability; FOCE: first-order conditional estimation; FOCE-I: first-order conditional estimation with the interaction; FOCE-LB: first-order conditional estimation method with the η-ε interaction option; GENDER: gender, 1 for male and 0 for female; GFR_MDRD_: four-variable modification of diet in renal disease estimated GFR (L/h); GOF: goodness-of-fit plot; Ka: absorption rate constant; KF: kidney function, KF = eGFR/(120 mL/min/ 1.73 m^2^); LSS: limited sampling strategy; NPDE: normalized prediction distribution errors; pcVPC: prediction-corrected visual predictive check; Q: intercompartment clearance; SAEM: stochastic approximation expectation maximization; SCR: serum creatinine concentration (μmol/L); Sex: for male, sex = 0 and for female, sex = 1; T: T = 0 for non-transplant patients and 0.76 for transplant patients; Tlag: lag time; V_c_: central volume of distribution; V_p_: peripheral volume of distribution; VPC: visual predictive check; θ_GraftType_: θ_kidney_ = 1.68, θ_heart_ = 0.86, θ_lung/liver_ = 1.17.

#### 3.2.2. Application of Model-Based Simulation

All studies performed model-based simulations. A variety of PK endpoints were utilized in simulations. AUC was used in 13 studies, while AUC_0–24h_ was most commonly used in 8 studies. AUC_0–12h_ and AUC_0-inf_ were also used. Four studies utilized C_trough_ as their PK endpoint. Nine studies proposed new dosing regimens to achieve targets [9,11,21,23,24,26,27,28,29]. Five studies evaluated existing dosing regimens [10,20,21,25,27]. Two studies performed efficacy and safety analyses [8,22], and two of the earliest studies evaluated the covariate effect on PK behaviors [18,19].

### 3.3. Overview of PPK Model Repository

#### 3.3.1. QC result

Similarity comparison was used to ensure accuracy of model repository construction. After excluding construction errors, the 95%CI of the PK parameters’ geometric mean was mainly distributed within 70–150% of the geometric means of same virtual patients.

PK behaviors of GCV in the same pediatric typical virtual patients from different PPK models were comparable (Supplementary Material Figure S1). However, t_1/2_ of Horvatits et al. [24] was larger than others because its original data came from critically ill patients receiving continuous venovenous hemodiafiltration (CVVHDF). It should be noted that the final PPK model had controlled for the extracorporeal therapy given to the patients. After oral administration of VGCV, the PK behaviors of GCV in the same typical virtual patients from most PPK models were also comparable (Supplementary Material Figure S2) except that virtual infants of Vezina et al. [25] showed lower t_1/2_ values, and this could be attributed to a limited 3 out of 95 subjects between 0 and 24 months, thus this model could not describe the process of GCV in typical virtual infants very well.

After QC and subsequent verification, the model repository quality was deemed satisfactory.

#### 3.3.2. Comparison of GCV and VGCV PK Profiles

**Figure 2:**
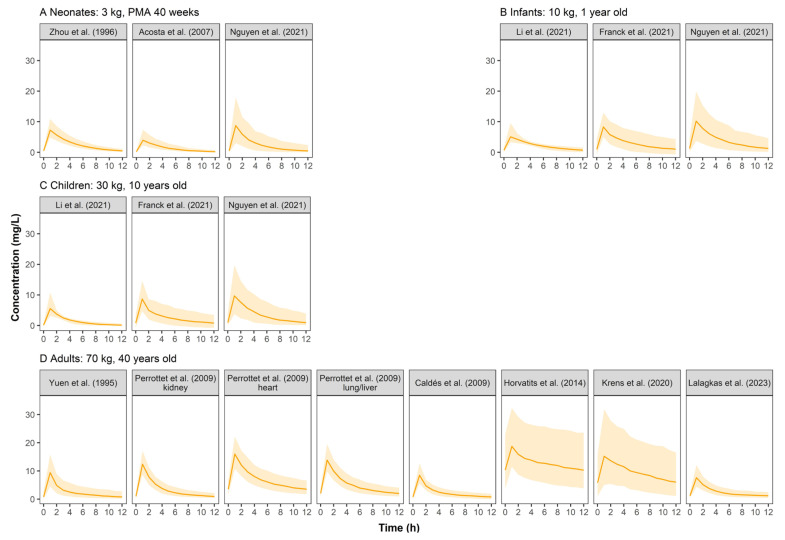
Simulated ganciclovir concentration–time profiles at steady state for neonates (**A**), infants (**B**), children (**C**), and adults (**D**) after intravenous infusion of ganciclovir in retrieved studies. The solid line represents the median of the simulated concentration–time profile. The light shadows represent the 10th–90th percentiles of the simulated concentration–time profiles. All patients were assumed to be males receiving GCV monotherapy at 5 mg/kg q12h.

Simulated GCV concentration–time profiles were displayed in Figure 2. The PK profiles across pediatrics were comparable because the weight-based dosing regimen had largely resolved PK differences between pediatrics, indicating that body weight could significantly influence GCV’s PK. Adults showed higher serum concentrations than pediatrics at a similar dose of 5 mg/kg. Furthermore, simulated concentrations based on the models established by Krens et al. [10] and Horvatits et al. [24] showed high variability when the dosing regimen of 5 mg/kg q12h was used.

**Figure 3:**
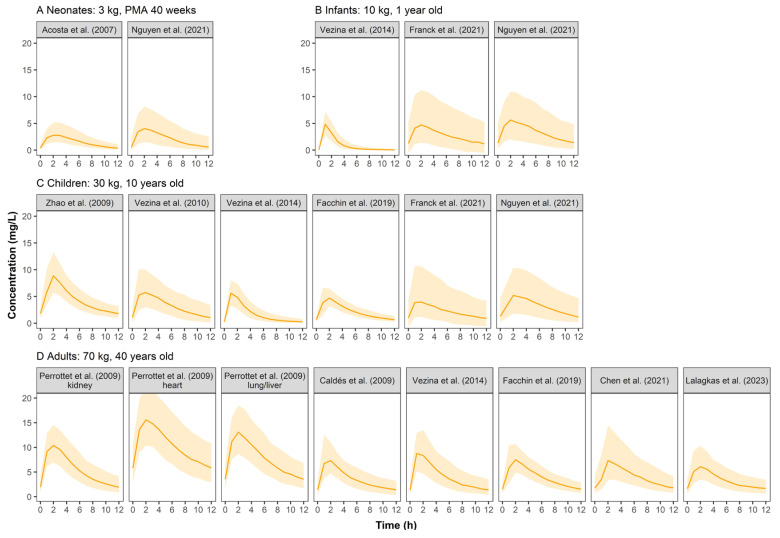
Simulated ganciclovir concentration–time profiles at steady state for neonates (**A**), infants (**B**), children (**C**), and adults (**D**) after oral administration of valganciclovir in retrieved studies. The solid line represents the median of the simulated concentration–time profile. The light shadows represent the 10th–90th percentiles of the simulated concentration–time profiles. All patients were assumed to be males. For neonates, infants and children, VGCV monotherapy was given at a dose of 10 mg/kg q12h, while 900 mg q12h was for adults.

Figure 3 shows the concentration–time profiles of VGCV. In pediatric groups, the simulated profiles were comparable between published models. Adults had higher concentrations than pediatrics.

#### 3.3.3. Covariate Screening and Covariate Effect

**Figure 4:**
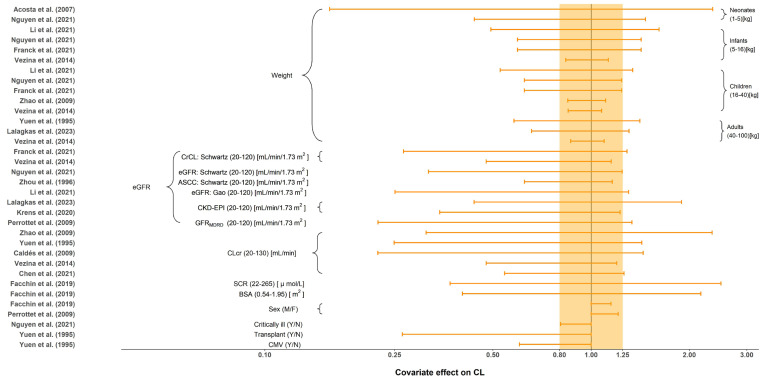
Covariate effect on the clearance of ganciclovir. The horizontal bars represent the covariate effect on clearance in each study. The typical value of clearance in each study was considered to be 1. The effect of each covariate for clearance is displayed by the ratio of clearance in the range of each covariate to the typical clearance value. The shaded area ranges from 0.8 to 1.25. ASCC: approximated creatinine clearance from serum; BSA: body surface area; CKD-EPI: the estimated glomerular filtration rate calculated by the Chronic Kidney Disease Epidemiology Collaboration (CKD-EPI) equation; CLcr: creatinine clearance (mL/min); CMV: cytomegalovirus; CrCL: creatinine clearance (mL/min/1.73 m^2^); eGFR: the estimated glomerular filtration rate; SCR: serum creatinine; Y: yes; N: no; M: male; F: female [9,10,11,18,19,20,21,22,23,25,26,27,28,29].

All tested covariates that had an effect on CL, distribution volume of central compartment (Vc), intercompartment clearance (Q) and distribution volume of the peripheral compartment (Vp) are summarized in Table 4. The stepwise method typically employed for covariate screening included forward inclusion and backward elimination. No covariates were investigated in Horvatits et al. [24] due to the limited number of included subjects (n = 9). The most influential covariates were body weight and renal function indicators, such as eGFR and CLcr. The impact of each covariate on CL is shown in Figure 4. Body size, including weight and BSA, were evaluated and included as significant covariates in 9 (56.3%) studies. Compared to the reference value, seven out of nine demonstrated significant impact of body size on CL with greater than 20% change under the normal range of body size [9,11,18,20,25,27,29]. Furthermore, the effect of renal function, such as CLcr, eGFR or SCR, was reported in 13 (81.3%) studies. Given the normal range of renal function, all of them demonstrated greater than 20% change in CL when compared to the reference value. Renal function affects renal clearance of GCV, which is the primary eliminate pathway of GCV. Only two studies investigated the influence of sex on CL (limited impact with less than a 20% difference) [22,28]. Yuen et al. [18] and Nguyen et al. [11] also investigated the effect of transplants, CMV-shedding, and critical illness on CL, where only transplant and CMV-shedding showed a significant influence. Body size and sex were reported to be the significant covariates on Vc. The only covariate identified on Q and Vp was weight.

### 3.4. Model Repository Applications

#### 3.4.1. Probability of Target Attainment

**Figure 5:**
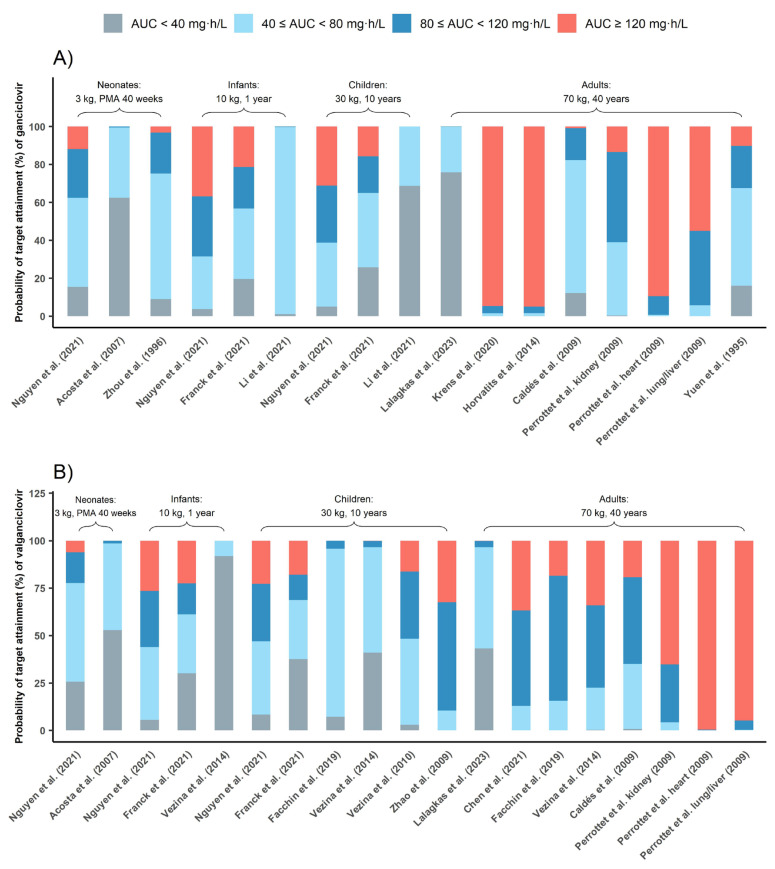
Probability of target attainment (%) of ganciclovir (**A**) and valganciclovir (**B**). The grey bar represents PTAs of AUC_0–24h_ below 40 mg·h/L, the red bar represents PTAs of AUC_0–24h_ above 120 mg·h/L, the blue bar represents PTAs of AUC_0–24h_ between 40 and 80 mg·h/L and the light blue bar represents PTAs of AUC_0–24h_ between 80 and 120 mg·h/L [9,10,11,18,19,20,21,22,23,25,26,27,28,29].

Figure 5 depicts PTA for commonly used dosing regimens based on published PPK models, with each model characterizing a specific population. Results indicated that 5 mg/kg q12h GCV dosing in adults carried a risk of overexposure (AUC_0–24h_ above 120 mg·h/L) with three out of six models suggesting that half of the subjects were overexposed [10,22,24]. According to the PTA of all PPK models involving adults (Table S2), 51.24% adults’ AUC_0–24h_ could exceed 120 mg·h/L with 5 mg/kg/12 h GCV dose regimen. In contrast, this dosing regimen showed good prophylaxis in most pediatric studies, 46.44% pediatrics could reach the prophylaxis target.

Further, 10 mg/kg/12 h VGCV was also effective in achieving prophylaxis in pediatrics, while 900 mg/12 h VGCV could lead to overexposure in adults.

#### 3.4.2. AUC calculator based on MAP-BE

As an example, we used the model established by Franck et al. [9] to develop a MAP-BE-based calculator for calculating the AUC_0–24h_ post-administration of GCV and VGCV. It requires the patient’s dosing and sampling information, weight, and creatinine clearance (CrCL, mL/min/1.73 m^2^) to calculate AUC_0–24h_ after the first dose. Its results were consistent with NONMEM (see the Supplementary Materials). This AUC calculator demo is available online. (https://ganciclovir.shinyapps.io/Example_GCVAUCCalculator/). Researchers can create similar calculators by modifying the model code (Supplementary Materials).

## 4. Discussion

To our best knowledge, this study was the first to build and share a parametric PPK model repository of GCV and VGCV. The repository is characterized by simulations of concentration–time profiles and covariate effect evaluation, where we demonstrated the potential of the constituent models in estimating the AUC and PTA of GCV and VGCV. The work provides evidence for individualized dosing based on the patient’s weight and renal function, as the commonly used dosing regimens tend to miss treatment targets in pediatrics.

### 4.1. Simulated Concentration–Time Profiles of Ganciclovir

The simulated concentration–time profiles (Figure 2) of most studies demonstrated a comparable curve in pediatrics when a dose of 5 mg/kg GCV was administered, suggesting that weight-based dosing is critical. Three studies’ (Horvatits et al. [24], Krens et al. [10], and Li et al. [29]) simulated profiles that were different from the others. We found that all three studies included critically ill patients in whom PK can be complicated by hemodynamic instability with varied volumes of distribution and fluctuating renal function. For the study of Horvatits et al., the model was also limited by the small number of patients (9 patients), with no covariates successfully included, and the IIV not clearly explained.

There were three other possible reasons why Li et al.’s PK profiles were different from others: (1) only 11.5% of the patients were children over six years old, so the 10-year-old virtual children did not match with the studied population; (2) the very sparse sampling strategy (138 samples from 104 subjects) might also limit the model performance; (3) the Gao formula [30] used in this study is more applicable to calculate eGFR of children with moderate renal failure.

### 4.2. Simulated Concentration–Time Profiles of Valganciclovir

The simulated concentration–time profiles of VGCV (Figure 3) were similar across pediatric studies except for Zhao et al. [23]. The patient population in Zhao et al. was unique as they were children who received a kidney transplant and mycophenolate mofetil as the immunosuppressant. It is known that mycophenolate mofetil could reduce the renal clearance of GCV, thus resulting in higher plasma concentrations in the simulation [31]. In Perrottet et al. [22], patients were stratified into three subgroups according to the type of organ transplant received. Patients who received a heart transplant had significantly higher exposure to GCV than the other two subgroups (liver and lung). The use of tacrolimus (heart subgroup) could have reduced renal blood flow to a greater extent than cyclosporine (liver and lung subgroups), resulting in the reduced renal elimination of GCV and the subsequent larger exposure to the drug [32]. Under the commonly used dosing regimen, the exposure level of VGCV in adults was similar to that of GCV at different ages. However, the exposure in pediatrics was significantly lower than GCV, indicating that dose optimization of VGCV for pediatrics is urgently needed.

### 4.3. Covariates Effects on Estimated PK Parameters

Eight of the 16 included studies identified body weight as a covariate on CL. Our results suggested that as age increased, the influence of weight on CL decreased. In neonates, the effect of weight on CL was marked with the clearance of those at the highest weight (5 kg) being at least 3.34 fold the CL in neonates weighing 1 kg. In contrast, the difference in clearance between the heaviest adult patient weighing 100 kg and the lightest patient weighing 40 kg was at most only 2.43 fold. This phenomenon may occur because pediatric patients are in a state of continuous growth and development, so the fluctuations are larger than in adults (the percentage of weight gain decreases with age). This is consistent with the notion that weight is often considered a significant covariate in pediatric population studies but is rarely included in adult studies.

Thirteen of the sixteen included studies identified renal function as a covariate on CL. GCV is mainly excreted in the urine through glomerular filtration and active tubular secretion. As such, the patient’s renal function would have a greater impact on the PK behavior of GCV. The forest plot suggested that regardless of the renal function indicator (i.e., eGFR, CLcr, SCR), the covariate effect on CL was clinically significant (outside the range of 80%–125%). This indicates that testing renal function’s influence is important when constructing the PPK model of GCV.

Two studies identified gender as a significant covariate, but the subsequent effect evaluation suggested that gender was not a clinically meaningful covariate on CL. The gender effect could manifest in other covariates, such as a discrepancy in body size.

### 4.4. Envisioning the Application of Model Repository

This study constructed a model repository of GCV and VGCV population pharmacokinetic models. Our research has the potential to significantly decrease the amount of time required for conducting literature searches. Leveraging our repository, other researchers can quickly perform external evaluations on their data to identify a suitable model or to explore the factors that influence the predictive ability of the model in different clinical settings. In addition, researchers can perform model averaging to reduce the impact of uncertainty in a single model and to identify the most appropriate predictions for individual patients, thus simplifying the process of precision dosing and reducing the burden of model validation.

New dosing regimens can be evaluated comprehensively using the models in the repository and the provided codes. Our work could significantly improve the efficiency of dosing regimens evaluation and the AUC calculator based on MAP-BE could be useful for determining AUC. The source code related to the PPK model could be modified to develop calculators that are based on new models. In addition, an AUC calculator can be built for each model in the repository, and medical staff can make decisions considering the AUC results calculated by multiple models.

The model repository can also be docked with the “PopED” package, and the published model in the repository can be selected as the prior information to optimize the experimental design. The package also supports the use of “rxode2” to build a preset model. The “nlmixr” package, as a free and open-source package, supports nonlinear mixed effects analysis in R, and can be seamlessly connected with the relevant code of the model repository, improving the efficiency of modeling.

### 4.5. Limitations

Our study has some limitations. Firstly, only the parametric PPK models were included in this study, and the non-parametric PPK models were excluded because the parameters of the non-parametric models were hard to bridge to parametric models. Secondly, in our simulation, we used several typical virtual patients in order to facilitate comparisons across studies. Hence, the simulation results may not be representative enough for the distribution of covariates in clinical practice. In other words, the typical patients may not represent the populations studied. Thirdly, the construction of the model repository was done manually in this study, and the next step could be to automatically generate a model repository using artificial intelligence technology [33].

## 5. Conclusions

The model repository of parametric population pharmacokinetic models for GCV and VGCV is useful for promoting MIPD. Optimization of the GCV and VGCV dosing regimen should consider the patient’s renal function. Our AUC calculator may provide a useful tool for clinicians to perform TDM during their routine work.

## Figures and Tables

**Table 1:** Details of typical virtual patients for simulation.

Virtual Patients	Neonates	Infants	Children	Adults
Sex	Male	Male	Male	Male
Age	40 weeks (PMA)	1 year old	10 years old	40 years old
Weight (kg)	3	10	30	70
Height (cm)	50	70	130	170
SCR (μmol/L)	30	50	70	95

PMA: postmenstrual age; SCR: serum creatinine.

**Table 2:** Characteristics of included population pharmacokinetic studies.

Study (Publication Year)	Country (Type of Study)	No. of Subjects (M/F)	Population Characteristic	No. of Observations	Sampling of Schedule	Age Mean ± SD Median (Range)	Weight (kg) Mean ± SD Median (Range]	Formulation	Dose Regimen Mean ± SD Median (Range]	Bioassay [LLOQ]
Lalagkas et al. (2023) [27]	Spain (Prospective)	60 (39/21)	Caucasian patients with established CMV infections undergoing allogeneic SOT (kidney, liver and heart)	640	IS ^1^ and SS ^2^	57 years (22–78)	68 (43–131)	GCV i.v. VGCV p.o.	GCV for CMV infection were 2.5 mg/kg/12 h, 2.5 mg/kg/24 h, and 1.25 mg/kg/24 h. VGCV for CMV infection were of 450 mg every 12, 24, and 48 h. For prophylaxis, VGCV was given at 450 mg every 24, 48 and 84 h	HPLC [0.5 mg/L]
Nguyen et al. (2021) [11]	France (Prospective)	105 (59/46)	Pediatrics	374	NR	2.5 years (0.01–17.3)	11.7 (2.6–80)	GCV i.v. VGCV p.o.	GCV: 10 mg/kg/day (1.2–15.4) VGCV: 36 mg/kg/day (14.6–83.8) GCV and VGCV were administered twice daily	LC–MS [0.05 μg/mL]
Franck et al. (2021) [9]	Canada (Retrospective)	50 (30/20)	Pediatric SOT and SCT recipients	580	IS ^1^ for GCV IS ^2^ for VGCV	7.5 years (0.5–17.4)	26.7 (5.96–87)	GCV i.v. VGCV p.o.	Pre-emptive approach for the prevention of CMV disease: 5 mg/kg/12 h GCV or 10 mg/kg/12 h VGCV	HPLC [0.039 mg/L]
Chen et al. (2021) [26]	China (Prospective)	70 (46/24)	Adult Chinese renal allograft recipients	768	IS ^3^	42.3 ± 9.95 years	61.1 ± 11.0	VGCV p.o.	450 mg/day 900 mg/day	LC–MS GCV: [0.048 mg/L] VGCV: [0.0048 mg/L]
Li et al. (2021) [29]	China (Retrospective)	104 (54/50)	Critically ill pediatric patients	138	NR	3.06 ± 2.99 years 2.46 years (0.10–12.83)	13.7 ± 8.3 12.0 (2.5–55.0)	GCV i.v.	5 mg/kg/12 h	HPLC [0.1 μg/mL]
Krens et al. (2020) [10]	Netherlands (Retrospective)	34 (17/17)	Critically ill patients	128	NR	56 years (30–82)	70 (44–140)	GCV i.v.	2.8 mg/kg/day (0.7–20)	HPLC [0.5 mg/L] LC–MS/MS [0.1 mg/L]
Facchin et al. (2019) [28]	France (Retrospective)	104 (66/38)	Children with renal transplant	1212	IS ^4^	12.2 years (2.1–20.5)	30.35 (11.9–83.0)	VGCV p.o.	18.5 mg/kg once a day or twice a day (5.0–70.2)	HPLC [0.25 mg/mL]
Horvatits et al. (2014) [24]	Australia (Prospective)	9 (8/1)	Critically ill patients with suspected or proven CMV infection	NR	IS ^5^	56 ± 9 years	86 ± 25	GCV i.v.	5 mg/kg in 0.5 h infusion via a central line	HPLC [5 ng/mL]
Vezina et al. (2014) [25]	United States (Prospective)	95 (60/35)	Pediatric and adult kidney, liver and lung transplant patients	269	SS	children: 0 to 24 months: 15 months (6–17) 2 to 11 years: 7 years (5–10) 12 to 17 years: 13 years (12–15) adults: 53 years (18–78)	children: 33 (6.9–61.1) adults: 71.8 (8.05–115)	VGCV tablet, oral solution	Most subjects: VGCV tablet, 900 mg every 24 h or 450 mg every 12, 24 or 48 h 8 subjects (7 children): VGCV oral solution, 350 mg, 300 mg, 270 mg, 225 mg, 150 mg or 75 mg every 24 h	HPLC [50 ng/mL]
Vezina et al. (2010) [8]	United States (Prospective)	8 (6/2)	Pediatric SOT patients at risk for Epstein–Barr virus disease	43	SS and IS ^6^	2.1 years (1.3–6.2)	14.1 (9.4–19.8)	VGCV suspension	11.1 mg/kg/12 h (10.1–12.1) 7.4 mg/kg/day (5.3–11.3)	HPLC [25 ng/mL]
Caldés et al. (2009) [21]	Spain (Prospective)	20 (10/10)	SOT recipients (kidney, liver or heart) with established CMV infection	382	IS ^7^	55.7 ± 11.8 years	66.2 ± 12.9	GCV i.v. VGCV p.o.	5 mg/kg/12 h GCV for 5 days followed by 900 mg/12 h VGCV for 16 days	HPLC [0.5 μg/mL]
Perrottet et al. (2009) [22]	Switzerland (Prospective)	65 (45/20)	SOT recipients (kidney, lung or heart)	437	SS and IS	55 years (18–70)	72 (46–115)	GCV i.v. VGCV p.o.	GCV: 5 mg/kg/12 h for treatment VGCV: 900 mg/12 h for treatment, 450 or 900 mg/day for prophylaxis	HPLC [0.1 μg/mL]
Zhao et al. (2009) [23]	France (Prospective)	22 (11/11)	Pediatric renal transplant patients	164	IS ^8^	10 ± 5 years 9 years (3–17)	34 ± 19 28 (12–76)	VGCV p.o.	Prophylactic therapy: 900 mg/24 h VGCV; Pre-emptive therapy: 5 mg/kg/12 h GCV for 15 days followed by 10 mg/kg/12 h VGCV for 3 months	HPLC [0.25 μg/mL]
Acosta et al. (2007) [20]	United States (Prospective)	24 (13/11)	Neonates with symptomatic congenital CMV disease	484	IS ^9^ and SS ^10^	study1.0: 30 days (11–34) study2.0: 20 days (8–33)	study1.0: 2.7 (2.1–3.4) study2.0: 2.9 (1.9–4.4)	GCV i.v. VGCV p.o.	GCV: 6 mg/kg/12 h VGCV: 14 mg/kg/12 h	LC–MS [0.4 μg/mL]
Zhou et al. (1996) [19]	United States (Prospective)	27 NR	Newborns with acute symptomatic CMV disease	219	NR	Newborns	NR	GCV i.v.	A single dose of 4 or 6 mg/kg, 1 h constant-rate infusion	HPLC [0.1 μg/mL]
Yuen et al. (1995) [18]	United States (Prospective)	53 NR	31 patients with CMV retinitis, 17 were shedding CMV in urine and 5 with SOT and renal dysfunction	558	SS ^11^ and IS ^12^	NR	NR	GCV i.v.	1.2–5.0 mg/kg, 1 h constant-rate infusion	HPLC [0.25 μg/mL]

CMV: cytomegalovirus; GCV: ganciclovir; HPLC: high-performance liquid chromatography; IS: intensive sampling; i.v.: intravenous administration; LC–MS: liquid chromatography-mass spectrometer; LLOQ: lower limit of quantitation; NR: not reported; p.o.: oral administration; SCT: stem-cell transplantation; SOT: solid-organ transplantation; SS: sparse sampling; VGCV: valganciclovir.^1^ IS: 0 (pre-dose), 0.5, 1, 1.5, 2, 3, 4, 6, 8, 10 and 12 h post dose. ^2^ SS: 0.5–1.5, 4–5 and 6–8 h post dose. ^3^ IS: 0 (pre-dose), 0.5, 1, 1.5, 2, 6 and 12 h post dose. ^4^ IS: 0 (pre-dose), 0.5, 0.75, 1, 1.5, 2, 6 and 12 h post dose. ^5^ IS: 0 (pre-dose), 0.5, 1, 1.5, 2, 3, 4, 6, 8, 12 and 24 h post dose. ^6^ IS: 1, 2, 4, 8, 12 and/or 24 h post dose. ^7^ IS: 0, 0.5, 1, 1.5, 3, 6, 8 and 24 h post dose. ^8^ IS: 0 (pre-dose), 1, 2, 4, 6, 8 and 12 h post dose. ^9^ IS: 0 (pre-dose), 0.5, 1, 1.5, 2, 3, 4, 6, 8, 12 and/or 24 h post dose. ^10^ IS: 0 (pre-dose), 1, 2, 4, 8, 12 and/or 16 and 24 h post dose. ^11^ IS: 0, 1, 2–3, 5–7 and 10–12 h post dose for study1.0; 0, 0.25–0.75, 1–3, 5–7 and 10–12 h post dose. ^12^ SS: 0.5 and 3 h post dose. ^13^ SS: blood samples were collected at irregular times. ^14^ IS: 0.5, 1, 2, 3, 4, 6, 8, 12, 15 and 24 h post dose.

**Table 4:** List of tested and significant covariates in included models.

Study (Publication Year)	Tested covariates			Covariate Selection Criteria		Significant Covariates			
	Demographic	Laboratory Tests	Co-Administration	Forward Inclusion	Backward Elimination	CL	Vc	Q	Vp
Lalagkas et al. (2023) [27]	Weight, BSA, lean body weight, total body water, sex, age	SCR	NR	*p* < 0.05	*p* < 0.01	CKD-EPI, weight	Weight	Weight	Weight
Nguyen et al. (2021) [11]	Weight, age, height, BSA, sex, critically ill	eGFR	NR	*p* < 0.05	*p* < 0.01	Weight, eGFR, critically ill	Weight	Weight	Weight
Franck et al. (2021) [9]	Weight, BSA, sex, age, ethnicity, transplant type, formulation	SCR, urea, CrCL	NR	*p* < 0.05	*p* < 0.01	Weight, CrCL	Weight	NR	Weight
Chen et al. (2021) [26]	Weight, sex, age, BSA,	CLcr	NR	*p* < 0.05	*p* < 0.01	CLcr	NR	NR	NR
Li et al. (2021) [29]	Weight, sex, age, height, BSA	BUN, SCR, UA, TBIL, ALT, AST, KF	NR	*p* < 0.05	*p* < 0.01	Weight, KF	Weight	NR	NR
Krens et al. (2020) [10]	Weight, sex, age, IBW, ethnicity, comorbidity, CVVH	SCR, CKD-EPI, serum sodium	NR	*p* < 0.05	*p* < 0.001	CKD-EPI	NR	NR	NR
Facchin et al. (2019) [28]	Weight, gender, age, height, BSA, underlying disease	SCR, serum uremia, proteinuria, CrCL	Mycophenolate mofetil, tacrolimus, cyclosporine, azathioprine	*p* < 0.05	*p* < 0.01	BSA, SCR, GENDER	BSA, GENDER	NR	NR
Horvatits et al. (2014) * [24]	NR	NR	NR	NR	NR	NR	NR	NR	NR
Vezina et al. (2014) [25]	Weight, sex, age, transplant type, donor source, recipient race, formulation, days post-transplant	SCR	Thymoglobulin, basiliximab, methylprednisolone, tacrolimus, ciclosporin or sirolimus	*p* < 0.005	*p* < 0.005	CLcr, weight	Weight	Weight	Weight
Vezina et al. (2010) [8]	Weight, sex, age, height, BSA, transplant type	CrCL	NR	*p* < 0.05	NR	NR	NR	NR	NR
Caldés et al. (2009) [21]	Weight, sex, age	CLcr	Cyclosporine, mycophenolate mofetil, sirolimus, tacrolimus	*p* < 0.05	*p* < 0.01	CLcr	NR	NR	NR
Perrottet et al. (2009) [22]	Weight, sex, age, height, transplant type, comorbidity	GFR	Cyclosporine, tacrolimus, mycophenolate, cotrimoxazole	NR	NR	GFR_MDRD_, sex	Weight, sex	NR	NR
Zhao et al. (2009) [23]	Weight, age, height	CLcr, AST, ALT, serum protein	Prednisone, mycophenolate mofetil	*p* < 0.01	*p* < 0.001	CLcr, weight	NR	NR	NR
Acosta et al. (2007) [20]	Weight, sex, PNA, BSA	NR	NR	*p* < 0.05	NR	Weight	Weight	NR	NR
Zhou et al. (1996) [19]	Weight	ASCC, PLAT	NR	*p* < 0.001	*p* < 0.005	ASCC	Weight	NR	NR
Yuen et al. (1995) [18]	Weight, transplant (yes/no)	CLcr, CMV-shedding or CMV retinitis	NR	*p* < 0.005	NR	Weight, CLcr, transplant(yes/no), CMV-shedding or CMV retinitis	Weight	NR	Weight

ASCC, approximated creatinine clearance from serum (mL/min/1.73 m^2^); ALT, alanine aminotransferase concentration; AST, aspartate aminotransferase concentration; BSA, body surface area; BUN, blood urea nitrogen; CKD-EPI, the estimated glomerular filtration rate calculated by the Chronic Kidney Disease Epidemiology Collaboration (CKD-EPI) equation; CLcr, creatinine clearance (mL/min); CMV, cytomegalovirus; CrCL, creatinine clearance (mL/min/1.73 m^2^); CVVH, application of continuous veno-venous hemofiltration; eGFR, the estimated glomerular filtration rate (mL/min/1.73 m^2^); GFRMDRD, four-variable modification of diet in renal disease estimated GFR (L/h); IBW, ideal body weight; KF, kidney function, KF = eGFR/(120 mL/min/ 1.73 m^2^); PLAT, platelet count; PNA, postnatal age; SCR, serum creatinine concentration; TBIL, total bilirubin concentration; UA, uric acid. *: Covariates were not included in the model due to the small number of patients (9 patients).

## Data Availability

The data generated during and/or analyzed during the current study are available from the corresponding author on reasonable request.

## Supplementary Materials

The following supporting information can be downloaded at: https://www.mdpi.com/article/10.3390/pharmaceutics15071801/s1, Table S1: The uniform covariate range and reference covariate value; Table S2: Total probability of target attainment of pediatrics and adults; Figure S1: Similarity comparison (%) of simulated pharmacokinetic profiles after intravenous infusion of ganciclovir; Figure S2: Similarity comparison (%) of simulated pharmacokinetic profiles after oral administration of valganciclovir.
