# Supplementary material for: Establishment and Evaluation of a Parametric Population Pharmacokinetic Model Repository for Ganciclovir and Valganciclovir
Source: Pharmaceutics. 2023 Jun 23;15(7):1801. doi: 10.3390/pharmaceutics15071801 (PMC10386724; doi:10.3390/pharmaceutics15071801)
Supplement: Supplementary file 1 [file pharmaceutics-15-01801-s001.zip › pharmaceutics-2399814-supplementary.pdf]

Contents of Supplementary Materials

Table S1. The uniform covariate range and reference covariate value ... 2
Table S2. Total probability of target attainment of pediatrics and adults. ... 3
Figure S1. Similarity comparison (%) of simulated pharmacokinetic profiles after intravenous infusion of ganciclovir. ... 4
Figure S2. Similarity comparison (%) of simulated pharmacokinetic profiles after oral administration of valganciclovir. ... 5
Search strategies ... 6
R Codes of Model Repository Establishment ... 8
- Lalagkas et al.(2023) ... 8
- Nguyen et al.(2021) ... 11
- Franck et al.(2021) ... 19
- Chen et al.(2021) ... 25
- Li et al.(2021) ... 27
- Krens et al.(2020) ... 31
- Facchin et al.(2019) ... 33
- Horvatits et al.(2014) ... 37
- Vezina et al.(2014) ... 39
- Vezina et al.(2010) ... 44
- Caldés et al.(2009) ... 46
- Perrottet et al.(2009) ... 49
- Zhao et al.(2009) ... 57
- Acosta et al.(2007) ... 59
- Zhou et al.(1996) ... 62
- Yuen et al.(1995) ... 64

R Codes of Covariate Effects Evaluation ... 79
R Codes of AUC Calculator based on MAP-BE ... 82
- # Globally used objects and functions ... 82
- # Define Shiny Server ... 89
- # Define UI of Shiny app ... 93

Validation of the $AUC_{0\text{-}24h}$ calculator with NONMEM ... 95

Table S1. The uniform covariate range and reference covariate value

Covariate		Covariate range	Reference covariate value
Weight (kg)	Neonates	1-5	3
	Infants	5-16	10
	Children	16-40	30
	Adults	40-100	70
eGFR (mL/min/1.73m^2)		20-120	90
CLcr (mL/min)		20-130	90

CLcr: creatinine clearance; eGFR: the estimated glomerular filtration rate.

Table S2. Total probability of target attainment of pediatrics and adults.

**Drug**	**Ganciclovir i.v.**		**Valganciclovir p.o.**	
**Population**	**Pediatrics**	**Adults**	**Pediatrics**	**Adults**
Dose regimen	5 mg/kg/12h	5 mg/kg/12h	10 mg/kg/12h	900 mg/12h
PTA (%) of AUC ≤ 40 mg·h/L	23.43	20.92	30.34	14.77
PTA (%) of 40 < AUC ≤ 80 mg·h/L	46.44	24.20	40.47	20.44
PTA (%) of 80 < AUC ≤ 120 mg·h/L	18.88	17.89	20.71	30.56
PTA (%) of AUC ≥ 120 mg·h/L	20.02	51.24	18.04	46.01

AUC: area-under-the-concentration-time-curve; i.v.: intravenous administration; p.o.: oral administration; PTA: probability of target attainment.

Figure S1. Similarity comparison (%) of simulated pharmacokinetic profiles after intravenous infusion of ganciclovir.

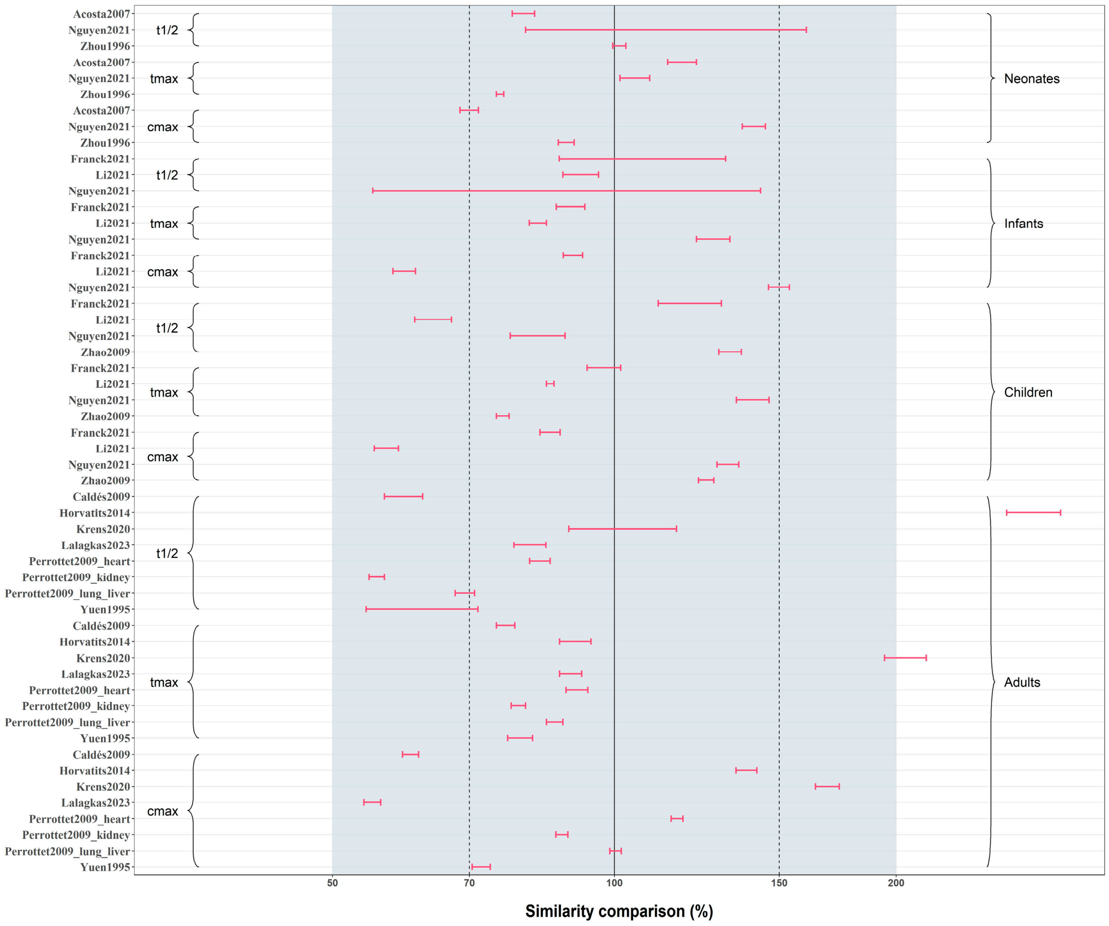

The red horizontal bars represent the geometric mean ratio with 95% CI of each model. The shaded gray area ranges from 50% to 200% of reference values. The geometic means of PK parameters in each age group was considered to be the reference values.

Figure S2. Similarity comparison (%) of simulated pharmacokinetic profiles after oral administration of valganciclovir.

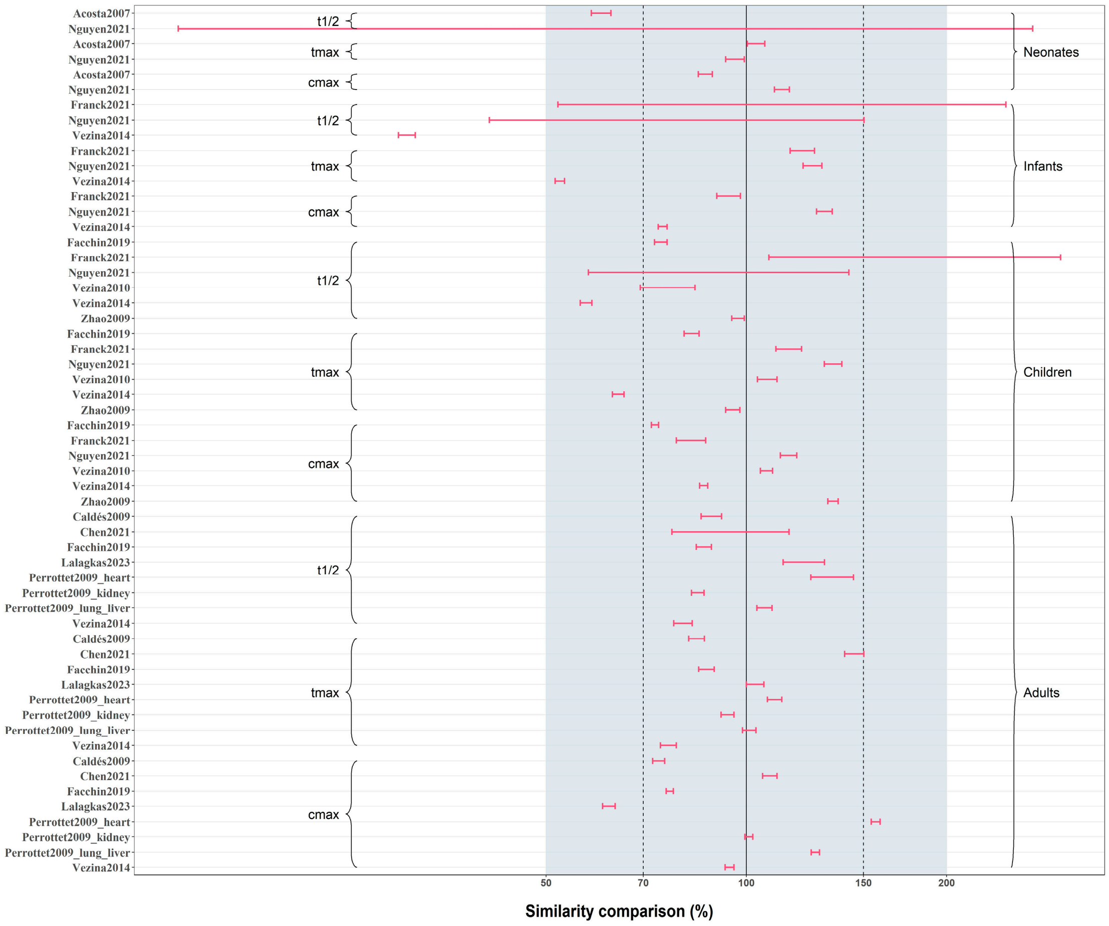

The red horizontal bars represent the geometric mean ratio with 95% CI of each model. The shaded gray area ranges from 50% to 200% of reference values. The geometic means of PK parameters in each age group was considered to be the reference values.

Search strategies

Search date: May 28, 2023

1. Search strategy of PubMed

'ganciclovir' or 'gancyclovir' or 'BW-759' or 'ganciclovir sodium' or 'Ganciclovir, Monosodium Salt' or 'RS-21592' or 'BIOLF-62' or 'Cytovene' or 'valganciclovir' or 'Ganciclovir L-valyl Ester' or 'Ganciclovir L valyl Ester' or 'Valcyt' or 'Valganciclovir Hydrochloride' or 'Valcyte' and 'population pharmacokinetic' or 'pharmacokinetic' or 'nonlinear mixed effect model' or 'NONMEM' or 'Pmetrics' or 'WINNONMIX' or 'ADAPT' or 'P-PHARM' or 'nlmixr' or 'NLME' or 'USC*PACK' or 'MONOLIX'.

Filters applied: Humans, English.

2. Search strategy of Embase

▼ Search History (5)

# ▲	Searches	Results	Type	Actions
1	exp ganciclovir/ or exp valganciclovir/	31483	Advanced	Display Results | More
2	population pharmacokinetic.mp.	8356	Advanced	Display Results | More
3	exp pharmacokinetic modeling software/	1872	Advanced	Display Results | More
4	2 or 3	9615	Advanced	Display Results | More
5	1 and 4	30	Advanced	Display Results | More

3. Search strategy of Scopus

(TITLE-ABS-KEY (ganciclovir*) OR TITLE-ABS-KEY (gancyclovir) OR TITLE-ABS-KEY (bw-759) OR TITLE-ABS-KEY (ganciclovir AND sodium) OR TITLE-ABS-KEY (ganciclovir, AND monosodium AND salt) OR TITLE-ABS-KEY (rs-21592) OR TITLE-ABS-KEY (biolf-62) OR TITLE-ABS-KEY (cytovene) OR TITLE-ABS-KEY (valganciclovir*) OR TITLE-ABS-KEY (ganciclovir AND l-valyl AND ester) OR TITLE-ABS-KEY (ganciclovir AND l AND valyl AND ester) OR TITLE-ABS-KEY (valcyt) OR TITLE-ABS-KEY (valganciclovir AND hydrochloride) OR TITLE-ABS-KEY (valcyte)) AND (TITLE-ABS-KEY (population AND pharmacokinetic) OR TITLE-ABS-KEY (pharmacokinetics) OR TITLE-ABS-KEY (nonlinear AND mixed AND effect AND model) OR TITLE-ABS-KEY (nonmem) OR TITLE-ABS-KEY (pmetrics) OR TITLE-ABS-KEY (winnonmix) OR TITLE-ABS-KEY (adapt) OR TITLE-ABS-KEY (p-pharm) OR TITLE-ABS-KEY (nlmixr) OR TITLE-ABS-KEY (nlme) OR TITLE-ABS-KEY (usc*pack) OR TITLE-ABS-KEY (monolix)) AND (EXCLUDE (DOCTYPE , "re")) AND (LIMIT-TO (LANGUAGE , "English")) AND (LIMIT-TO (EXACTKEYWORD , "Ganciclovir") OR LIMIT-TO (EXACTKEYWORD , "Human") OR LIMIT-TO (EXACTKEYWORD , "Humans") OR LIMIT-TO (EXACTKEYWORD , "Male") OR LIMIT-TO (EXACTKEYWORD , "Female") OR LIMIT-TO (EXACTKEYWORD , "Adult") OR LIMIT-TO (EXACTKEYWORD , "Valganciclovir") OR LIMIT-TO (EXACTKEYWORD , "Middle Aged") OR LIMIT-TO (EXACTKEYWORD , "Child") OR LIMIT-TO (EXACTKEYWORD , "Aged") OR LIMIT-TO (EXACTKEYWORD , "Adolescent") OR LIMIT-TO (EXACTKEYWORD , "Infant") OR LIMIT-TO (EXACTKEYWORD , "Child, Preschool") OR LIMIT-TO (EXACTKEYWORD , "Preschool Child") OR EXCLUDE (EXACTKEYWORD , "Nonhuman") OR EXCLUDE

(EXACTKEYWORD , "Human Cell") OR EXCLUDE (EXACTKEYWORD , "Human Tissue") OR EXCLUDE (EXACTKEYWORD , "Animals") OR LIMIT-TO (EXACTKEYWORD , "Newborn") OR LIMIT-TO (EXACTKEYWORD , "Infant, Newborn") OR LIMIT-TO (EXACTKEYWORD , "Young Adult") OR LIMIT-TO (EXACTKEYWORD , "School Child") OR LIMIT-TO (EXACTKEYWORD , "Children"))

4. Search strategy of Web of Science

("ganciclovir" or "gancyclovir" or "BW-759" or "ganciclovir sodium" or "Ganciclovir, Monosodium Salt" or "RS-21592" or "BIOLF-62" or "Cytovene" or "valganciclovir" or "Ganciclovir L-valyl Ester" or "Ganciclovir L valyl Ester" or "valcyt" or "valganciclovir hydrochloride" or "valcyte") AND ("population pharmacokinetic" or "pharmacokinetic" or "nonlinear mixed effect model" or "NONMEM" or "Pmetrics" or "WINNONMIX" or "ADAPT" or "P-PHARM" or "nlmixr" or "NLME" or "USC*PACK" or "MONOLIX")

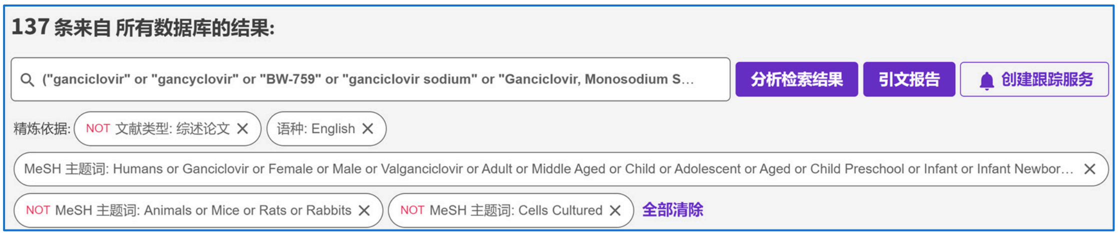

Inclusion criteria:

Inclusion criteria were imposed during study selection. Eligible papers must also meet the following criteria:

(1) GCV was administered intravenously (papers with oral administration were excluded)

(2) VGCV was administered orally

(3) PPK modeling was undertaken via a parametric non-linear mixed effect approach.

Exclusion criteria:

We excluded studies that fulfilled the following criteria:

(1) non-human studies,

(2) not written in English,

(3) review or methodology studies,

(4) non-PPK studies,

(5) studies that used duplicate data or overlapping cohorts with other studies (we selected the study with a larger sample size and excluded the rest)

(6) incomplete PPK parameter or covariate information.

R Codes of Model Repository Establishment

```r
# Establish a model repository of parametric PPK models for GCV and
VGCV using RxODE
# Author: Wenyu Yang
# Email: 21211030109@m.fudan.edu.cn

rm(list=ls())
#set working directory to current folder
curr.dir<-dirname(rstudioapi::getActiveDocumentContext()$path)
setwd(curr.dir)

# load R packages
library(tidyverse) # for data visualisation and manipulation
library(rxode2) # for simulation
library(cowplot) # for combine figures

#create fold for figure output
output_dir <- "simPK"
if (!file.exists(output_dir)) {dir.create (output_dir)}
```

Lalagkas et al.(2023)

```r
# 1.##Lalagkas et al.(2023)## ---------------------------------------
--------------
# Define model ------------------------------------------------------
------
# CL = 6.93 × (CKD-EPI/55)^0.817 X (BW/70)^0.75 (L/h)
# V2 = 43.1 × (BW/70) (L)
# Q  = 9.23 x (BW/70)^0.75 (L/h)
# V3 = 219 x (BW/70) (L)
# Ka = 0.766 (h-1)
# F1 = 0.699
# Tlag = 0.331 (h)
# BSV (CV%): CL = 29.9%, V2 = 36.1%, V3 = 103.4%, Ka = 45.7%, F1 =
16.6%
# prop.err = 0.282, add.err = 0.237 mg/L
set.seed(123456)
rxSetSeed(123456)

mod1 <- rxode2({
```

```
  CL   = TVCL*exp(eta.CL);
  V2   = TVV2*exp(eta.V2);
  Q    = TVQ;
  V3   = TVV3*exp(eta.V3);
  KA   = TVKA*exp(eta.KA);
  F1   = TVF1*exp(eta.F1);

  C2 = centr/V2;
  C3 = peri/V3;
  d/dt(depot) = -KA*depot;
  d/dt(centr) =  F1*KA*depot - CL*C2 - Q*C2 + Q*C3;
  d/dt(peri)  = Q*C2 - Q*C3;
  alag(depot) = TLAG;

  cp = C2*(1 + prop.err.sd) + add.err.sd;
})


# Adults -------------------------------------------------------------
------
# Define typical patient: adults
BW <- 70   # kg
SCR <- 95/88.4 # mg/dL (1mg/dL = 88.4umol/L)
AGE <- 40 # years
k <- 0.9 # k = 9 for males, k = 0.7 for females
a <- 0.411 # a = 0.411 for males, a = 0.329 for females
CKD_EPI <- 141*min(SCR/k,1)^a*max(SCR/k,1)^-1.209*0.993^AGE

# Define fixed effect parameters
theta <- c(TVCL=6.93*(CKD_EPI/55)^0.817*(BW/70)^0.75,
         TVV2=43.1*(BW/70),
         TVQ=9.23*(BW/70)^0.75,
         TVV3=219*(BW/70),
         TVKA=0.766,
         TVF1=0.699,
         TLAG=0.331)

# Define between subject variability
omega <- lotri(eta.CL ~ 0.0893, eta.V2 ~ 0.13,
             eta.V3 ~ 1.07, eta.KA ~ 0.209, eta.F1 ~ 0.0275)

# Define unexplained variability
sigma <- lotri(prop.err.sd ~ 0.282^2, add.err.sd ~ 0.237^2)
```

```
# DOSE of GCV(iv) input ---------------------------------------------
---
dose_gcv <- 5*BW # 5 mg/kg/12h

# Define event record
ev1 <- et(amount.units = "mg", time.units = "hours") %>%
  add.dosing(dosing.to = "centr",
             dose = dose_gcv,  # mg
             rate = dose_gcv/1, # infusion for 1h
             nbr.doses = 14,
             dosing.interval = 12,
             start.time = 0) %>%
  # 0-12h after the last dose
  add.sampling(seq(from=156,to=168,by=1))

# Perform simulation
# total number of subject: 1000
sim1 <- rxSolve(mod1,theta,ev1,omega=omega,sigma=sigma,nSub=1000)

# Concentration
pk_gcv <- sim1 %>%
  group_by(time) %>%
  # 10-90% Prediction interval
  summarise(medconc = median(cp),
            lowconc = quantile(cp,0.1),
            highconc = quantile(cp, 0.9)) %>%
  ungroup() %>%
  # time after last dose
  mutate(tald = time-156)

dat1 <- pk_gcv %>%
  mutate(study="Lalagkas2023",
         drug="GCV",
         pop="adult")

# DOSE of VGCV(oral) input ------------------------------------------
---
dose_vgcv <- 900*0.72 # 900 mg/12h × 0.72 (the ratio between the
molecular weights of GCV and VGCV)

# Define event record
ev2 <- et(amount.units = "mg", time.units = "hours") %>%
  add.dosing(dosing.to = "depot",
             dose = dose_vgcv,  # mg
```

```
            nbr.doses = 14,
            dosing.interval = 12,
            start.time = 0) %>%
  # 0-12h after the last dose
  add.sampling(seq(from=156,to=168,by=1))

# Perform simulation
# total number of subject: 1000
sim2 <- rxSolve(mod1,theta,ev2,omega=omega,sigma=sigma,nSub=1000)

# Concentration
pk_vgcv <- sim2 %>%
  group_by(time) %>%
  # 10-90% Prediction interval
  summarise(medconc = median(cp),
           lowconc = quantile(cp,0.1),
           highconc = quantile(cp, 0.9)) %>%
  ungroup() %>%
  # time after last dose
  mutate(tald = time-156)


dat2 <- pk_vgcv %>%
  mutate(study="Lalagkas2023",
         drug="VGCV",
         pop="adult")
```

Nguyen et al.(2021)

```
# 2.##Nguyen et al.(2021)## -----------------------------------------
-------------
# Define model ------------------------------------------------------
------
# Ka = 0.506 (h-1)
# CL = 2.55 × (BW/11.7)^0.75 × (eGFR/167)^0.763 X 0.806^critically
ill  (L/h)
# V2 = 5.96 × (BW/11.7) (L)
# Q  = 0.222 x (BW/11.7)^0.75 (L/h)
# V3 = 1.29 x (BW/11.7) (L)
# F1 = 0.438
# BSV (CV%): CL = 48.6%, V2 = 46.9%
# prop.err = 0.477
```

```
# eGFR: estimated glomerular filtration rate (mL/min/1.73 m2); BW
(kg)

# Typical patient1: children(boys),10 years
old,BW=30kg,HT=130cm,SCR=70umol/L
# Typical patient2:
neonates(boys),PMA=40weeks,HT=50cm,BW=3kg,SCR=30umol/L
# Typical patient3: infants(boys),1 year
old,HT=70cm,BW=10kg,SCR=50umol/L
# eGFR = k*HT(cm)/SCR(mg/dL), calculated by Schwartz formula
# critically ill: 1 for critically ill patients and 0 for others,
choose 0
# Dose: GCV iv, 5 mg/kg/12h, inf=1h; VGCV oral, 10 mg/kg/12h

set.seed(123456)
rxSetSeed(123456)

mod2 <- rxode2({
  CL   = TVCL*exp(eta.CL);
  V2   = TVV2*exp(eta.V2);
  Q    = TVQ;
  V3   = TVV3;
  KA   = TVKA;
  F1   = TVF1;

  C2 = centr/V2;
  C3 = peri/V3;
  d/dt(depot)  = -KA*depot;
  d/dt(centr)  =  F1*KA*depot - CL*C2 - Q*C2 + Q*C3;
  d/dt(peri)   = Q*C2 - Q*C3;

  cp = C2*(1 + prop.err.sd);
})


# Children -----------------------------------------------------------
------
# Define typical patient: children
BW <- 30   # kg
HT <- 130  # cm
SCR <- 70/88.4 # mg/dL (1mg/dL = 88.4umol/L)
k <- 0.55 # k = 0.55 for boys aged 2 years to less than 13 years
eGFR <- k*HT/SCR # ~90mL/min/1.73 m2, calculated by Schwartz formula
```

```r
# Define fixed effect parameters
theta <- c(TVCL=2.55*((BW/11.7)^0.75)*(eGFR/167)^0.763*0.806^0,
           TVV2=5.96*(BW/11.7),
           TVQ=0.222*(BW/11.7)^0.75,
           TVV3=1.29*(BW/11.7),
           TVKA=0.506,
           TVF1=0.438)

# Define between subject variability
omega <- lotri(eta.CL ~ 0.486^2, eta.V2 ~ 0.469^2)

# Define unexplained variability
sigma <- lotri(prop.err.sd ~ 0.477^2)

# DOSE of GCV(iv) input ---------------------------------------------
---
dose_gcv <- 5*BW # 5 mg/kg/12h for children

# Define event record
ev1 <- et(amount.units = "mg", time.units = "hours") %>%
  add.dosing(dosing.to = "centr",
             dose = dose_gcv,  # mg
             rate = dose_gcv/1, # infusion for 1h
             nbr.doses = 14,
             dosing.interval = 12,
             start.time = 0) %>%
  # 0-12h after the last dose
  add.sampling(seq(from=156,to=168,by=1))

# Perform simulation
# total number of subject: 1000
sim1 <- rxSolve(mod2,theta,ev1,omega=omega,sigma=sigma,nSub=1000)

# Concentration
pk_gcv <- sim1 %>%
  group_by(time) %>%
  # 10-90% Prediction interval
  summarise(medconc = median(cp),
            lowconc = quantile(cp,0.1),
            highconc = quantile(cp, 0.9)) %>%
  ungroup() %>%
  # time after last dose
  mutate(tald = time-156)
```

```r
dat3 <- pk_gcv %>%
  mutate(study="Nguyen2021",
         drug="GCV",
         pop="child")

# DOSE of VGCV(oral) input -----------------------------------------
---
dose_vgcv <- 10*BW # 10 mg/kg/12h for children

# Define event record
ev2 <- et(amount.units = "mg", time.units = "hours") %>%
  add.dosing(dosing.to = "depot",
             dose = dose_vgcv,  # mg
             nbr.doses = 14,
             dosing.interval = 12,
             start.time = 0) %>%
  # 0-12h after the last dose
  add.sampling(seq(from=156,to=168,by=1))

# Perform simulation
# total number of subject: 1000
sim2 <- rxSolve(mod2,theta,ev2,omega=omega,sigma=sigma,nSub=1000)

# Concentration
pk_vgcv <- sim2 %>%
  group_by(time) %>%
  # 10-90% Prediction interval
  summarise(medconc = median(cp),
            lowconc = quantile(cp,0.1),
            highconc = quantile(cp, 0.9)) %>%
  ungroup() %>%
  # time after last dose
  mutate(tald = time-156)

# make the margin smooth by lowess
l0.1 <- lowess(pk_vgcv$tald, pk_vgcv$lowconc, f=0.35)
l0.5 <- lowess(pk_vgcv$tald, pk_vgcv$medconc, f=0.35)
l0.9 <- lowess(pk_vgcv$tald, pk_vgcv$highconc, f=0.35)

df0.1 <- data.frame(tald=l0.1$x,lowconc=l0.1$y)
df0.5 <- data.frame(tald=l0.5$x,medconc=l0.5$y)
df0.9 <- data.frame(tald=l0.9$x,highconc=l0.9$y)

vgcv_dat <- df0.1 %>%
```

```r
  left_join(df0.5,by="tald") %>%
  left_join(df0.9,by="tald")

dat4 <- vgcv_dat %>%
  mutate(study="Nguyen2021",
         drug="VGCV",
         pop="child")

# Neonates -----------------------------------------------------------
------
# Define typical patient: neonates
BW <- 3   # kg
HT <- 50  # cm
SCR <- 30/88.4 # mg/dL (1mg/dL = 88.4umol/L)
k <- 0.45 # k = 0.45 for subjects aged less than 2 years;
eGFR <- k*HT/SCR # ~ 66 mL/min/1.73 m2, calculated by Schwartz
formula

# Define fixed effect parameters
theta <- c(TVCL=2.55*((BW/11.7)^0.75)*(eGFR/167)^0.763*0.806^0,
          TVV2=5.98*(BW/11.7),
          TVQ=0.222*(BW/11.7)^0.75,
          TVV3=1.29*(BW/11.7),
          TVKA=0.506,
          TVF1=0.438)

# Define between subject variability
omega <- lotri(eta.CL ~ 0.486^2, eta.V2 ~ 0.469^2)

# Define unexplained variability
sigma <- lotri(prop.err.sd ~ 0.477^2)


# DOSE of GCV(iv) input ----------------------------------------------
---
dose_gcv <- 5*BW # 5 mg/kg/12h for neonates

# Define event record
ev3 <- et(amount.units = "mg", time.units = "hours") %>%
  add.dosing(dosing.to = "centr",
           dose = dose_gcv,  # mg
           rate = dose_gcv/1, # infusion for 1h
           nbr.doses = 14,
           dosing.interval = 12,
```

```
          start.time = 0) %>%
  # 0-12h after the last dose
  add.sampling(seq(from=156,to=168,by=1))

# Perform simulation
# total number of subject: 1000
sim3 <- rxSolve(mod2,theta,ev3,omega=omega,sigma=sigma,nSub=1000)

# Concentration
pk_gcv <- sim3 %>%
  group_by(time) %>%
  # 10-90% Prediction interval
  summarise(medconc = median(cp),
          lowconc = quantile(cp,0.1),
          highconc = quantile(cp, 0.9)) %>%
  ungroup() %>%
  # time after last dose
  mutate(tald = time-156)


dat5 <- pk_gcv %>%
  mutate(study="Nguyen2021",
        drug="GCV",
        pop="neonate")

# DOSE of VGCV(oral) input -----------------------------------------
---
dose_vgcv <- 10*BW # 10 mg/kg/12h for neonates

# Define event record
ev4 <- et(amount.units = "mg", time.units = "hours") %>%
  add.dosing(dosing.to = "depot",
          dose = dose_vgcv,  # mg
          nbr.doses = 14,
          dosing.interval = 12,
          start.time = 0) %>%
  # 0-12h after the last dose
  add.sampling(seq(from=156,to=168,by=1))

# Perform simulation
# total number of subject: 1000
sim4 <- rxSolve(mod2,theta,ev4,omega=omega,sigma=sigma,nSub=1000)

# Concentration
```

```
pk_vgcv <- sim4 %>%
  group_by(time) %>%
  # 10-90% Prediction interval
  summarise(medconc = median(cp),
           lowconc = quantile(cp,0.1),
           highconc = quantile(cp, 0.9)) %>%
  ungroup() %>%
  # time after last dose
  mutate(tald = time-156)


dat6 <- pk_vgcv %>%
  mutate(study="Nguyen2021",
        drug="VGCV",
        pop="neonate")

# Infants ------------------------------------------------------------
-----
# Define typical patient: infants
BW <- 10   # kg
HT <- 70  # cm
SCR <- 50/88.4 # mg/dL (1mg/dL = 88.4umol/L)
k <- 0.45 # k = 0.45 for subjects aged less than 2 years;
eGFR <- k*HT/SCR # ~ 55.7 mL/min/1.73 m2, calculated by Schwartz
formula

# Define fixed effect parameters
theta <- c(TVCL=2.55*((BW/11.7)^0.75)*(eGFR/167)^0.763*0.806^0,
          TVV2=5.98*(BW/11.7),
          TVQ=0.222*(BW/11.7)^0.75,
          TVV3=1.29*(BW/11.7),
          TVKA=0.506,
          TVF1=0.438)

# Define between subject variability
omega <- lotri(eta.CL ~ 0.486^2, eta.V2 ~ 0.469^2)

# Define unexplained variability
sigma <- lotri(prop.err.sd ~ 0.477^2)


# DOSE of GCV(iv) input ---------------------------------------------
---
dose_gcv <- 5*BW # 5 mg/kg/12h for infants
```

```
# Define event record
ev5 <- et(amount.units = "mg", time.units = "hours") %>%
  add.dosing(dosing.to = "centr",
             dose = dose_gcv,  # mg
             rate = dose_gcv/1, # infusion for 1h
             nbr.doses = 14,
             dosing.interval = 12,
             start.time = 0) %>%
  # 0-12h after the last dose
  add.sampling(seq(from=156,to=168,by=1))

# Perform simulation
# total number of subject: 1000
sim5 <- rxSolve(mod2,theta,ev5,omega=omega,sigma=sigma,nSub=1000)

# Concentration
pk_gcv <- sim5 %>%
  group_by(time) %>%
  # 10-90% Prediction interval
  summarise(medconc = median(cp),
            lowconc = quantile(cp,0.1),
            highconc = quantile(cp, 0.9)) %>%
  ungroup() %>%
  # time after last dose
  mutate(tald = time-156)

dat7 <- pk_gcv %>%
  mutate(study="Nguyen2021",
         drug="GCV",
         pop="infant")

# DOSE of VGCV(oral) input -----------------------------------------
---
dose_vgcv <- 10*BW # 10 mg/kg/12h for infants

# Define event record
ev6 <- et(amount.units = "mg", time.units = "hours") %>%
  add.dosing(dosing.to = "depot",
             dose = dose_vgcv,  # mg
             nbr.doses = 14,
             dosing.interval = 12,
             start.time = 0) %>%
  # 0-12h after the last dose
```

```
  add.sampling(seq(from=156,to=168,by=1))

# Perform simulation
# total number of subject: 1000
sim6 <- rxSolve(mod2,theta,ev6,omega=omega,sigma=sigma,nSub=1000)

# Concentration
pk_vgcv <- sim6 %>%
  group_by(time) %>%
  # 10-90% Prediction interval
  summarise(medconc = median(cp),
           lowconc = quantile(cp,0.1),
           highconc = quantile(cp, 0.9)) %>%
  ungroup() %>%
  # time after last dose
  mutate(tald = time-156)


dat8 <- pk_vgcv %>%
  mutate(study="Nguyen2021",
        drug="VGCV",
        pop="infant")
```

Franck et al.(2021)

```
# 3.##Franck et al.(2021)## -----------------------------------------
-------------
# Define model -------------------------------------------------------
------
# CL   = 6.9 × (BW/26.7)^0.75 × (CrCL/149.8)^0.88 (L/h)
# V2   = 9.7 × (BW/26.7) (L)
# Q    = 10.9 (L/h)
# V3   = 7.6 x (BW/26.7) (L)
# Ka   = 0.73 (h-1)
# F1   = 0.43
# Tlag = 0.33 (h)

# BSV (CV%): CL = 66.3%, V2 = 76.8%, Ka = 83.7%, F1 = 55.7%,
# add.err = 0.98 mg/L
# CrCL:  creatinine clearance (mL/min/1.73 m2); BW (kg)

# Typical patient1: children(boys),10 years old,BW=30kg,SCR=70umol/L
```

```
# Typical patient2: infants(boys),1 year
old,HT=70cm,BW=10kg,SCR=50umol/L
# Dose: GCV iv, 5 mg/kg/12h, infusion=1h; VGCV oral, 10 mg/kg/12h

set.seed(1234)
rxSetSeed(1234)

mod3 <- rxode2({
  CL   = TVCL*exp(eta.CL);
  V2   = TVV2*exp(eta.V2);
  Q    = TVQ;
  V3   = TVV3;
  KA   = TVKA*exp(eta.KA);
  F1   = TVF1*exp(eta.F1);

  C2 = centr/V2;
  C3 = peri/V3;
  d/dt(depot) = -KA*depot;
  d/dt(centr) =  F1*KA*depot - CL*C2 - Q*C2 + Q*C3;
  d/dt(peri)  = Q*C2 - Q*C3;
  alag(depot) = TLAG;

  cp = C2 + add.err.sd;
})


# Children -----------------------------------------------------------
------
# Define typical patient: children
BW <- 30   # kg
HT <- 130  # cm
SCR <- 70/88.4 # mg/dL (1mg/dL = 88.4umol/L)
k <- 0.55 # k = 0.55 for boys aged 2 years to less than 13 years
CrCL <- k*HT/SCR # ~90mL/min/1.73 m2, calculated by modified Schwartz
formula

# Define fixed effect parameters
theta <- c(TVCL=6.9*((BW/26.7)^0.75)*((CrCL/149.8)^0.88),
         TVV2=9.7*(BW/26.7),
         TVQ =10.9,
         TVV3=7.6*(BW/26.7),
         TVKA=0.73,
         TVF1=0.43,
         TLAG=0.33)
```

```
# Define between subject variability
omega <- lotri(eta.CL ~ 0.663^2, eta.V2 ~ 0.768^2,
             eta.KA ~ 0.837^2, eta.F1 ~ 0.557^2)

# Define unexplained variability
sigma <- lotri(add.err.sd ~ 0.98^2)


# DOSE of GCV(iv) input --------------------------------------------
---
dose_gcv <- 5*BW # 5 mg/kg/12h for children

# Define event record
ev1 <- et(amount.units = "mg", time.units = "hours") %>%
  add.dosing(dosing.to = "centr",
           dose = dose_gcv,  # mg
           rate = dose_gcv/1, # infusion for 1h
           nbr.doses = 14,
           dosing.interval = 12,
           start.time = 0) %>%
  # 0-12h after the last dose
  add.sampling(seq(from=156,to=168,by=1))

# Perform simulation
# total number of subject: 1000
sim1 <- rxSolve(mod3,theta,ev1,omega=omega,sigma=sigma,nSub=1000)

# Concentration
pk_gcv <- sim1 %>%
  group_by(time) %>%
  # 10-90% Prediction interval
  summarise(medconc = median(cp),
          lowconc = quantile(cp,0.1),
          highconc = quantile(cp, 0.9)) %>%
  ungroup() %>%
  # time after last dose
  mutate(tald = time-156)

# make the margin smooth by lowess
l0.1 <- lowess(pk_gcv$tald, pk_gcv$lowconc, f=0.35)
l0.5 <- lowess(pk_gcv$tald, pk_gcv$medconc, f=0.35)
l0.9 <- lowess(pk_gcv$tald, pk_gcv$highconc, f=0.35)
```

```
df0.1 <- data.frame(tald=l0.1$x,lowconc=l0.1$y)
df0.5 <- data.frame(tald=l0.5$x,medconc=l0.5$y)
df0.9 <- data.frame(tald=l0.9$x,highconc=l0.9$y)

gcv_dat <- df0.1 %>%
  left_join(df0.5,by="tald") %>%
  left_join(df0.9,by="tald")

dat9 <- pk_gcv %>%
  mutate(study="Franck2021",
         drug="GCV",
         pop="child")

# DOSE of VGCV(oral) input ------------------------------------------
---
dose_vgcv <- 10*BW # 10 mg/kg/12h for children

# Define event record
ev2 <- et(amount.units = "mg", time.units = "hours") %>%
  add.dosing(dosing.to = "depot",
           dose = dose_vgcv,  # mg
           nbr.doses = 14,
           dosing.interval = 12,
           start.time = 0) %>%
  # 0-12h after the last dose
  add.sampling(seq(from=156,to=168.33,by=1))

# Perform simulation
# total number of subject: 1000
sim2 <- rxSolve(mod3,theta,ev2,omega=omega,sigma=sigma,nSub=1000)

# Concentration
pk_vgcv <- sim2 %>%
  group_by(time) %>%
  # 10-90% Prediction interval
  summarise(medconc = median(cp),
          lowconc = quantile(cp,0.1),
          highconc = quantile(cp, 0.9)) %>%
  ungroup() %>%
  # time after last dose
  mutate(tald = time-156)


dat10 <- pk_vgcv %>%
```

```
  mutate(study="Franck2021",
         drug="VGCV",
         pop="child")

# Infants -----------------------------------------------------------
-----
# Define typical patient: children
BW <- 10   # kg
HT <- 70  # cm
SCR <- 50/88.4 # mg/dL (1mg/dL = 88.4umol/L)
k <- 0.45 # k = 0.45 for subjects aged less than 2 years;
CrCL <- k*HT/SCR # ~ 55.7 mL/min/1.73 m2, calculated by modified
Schwartz formula

# Define fixed effect parameters
theta <- c(TVCL=6.9*((BW/26.7)^0.75)*((CrCL/149.8)^0.88),
         TVV2=9.7*(BW/26.7),
         TVQ =10.9,
         TVV3=7.6*(BW/26.7),
         TVKA=0.73,
         TVF1=0.43,
         TLAG=0.33)

# Define between subject variability
omega <- lotri(eta.CL ~ 0.663^2, eta.V2 ~ 0.768^2,
             eta.KA ~ 0.837^2, eta.F1 ~ 0.557^2)

# Define unexplained variability
sigma <- lotri(add.err.sd ~ 0.98^2)


# DOSE of GCV(iv) input --------------------------------------------
---
dose_gcv <- 5*BW # 5 mg/kg/12h for children

# Define event record
ev3 <- et(amount.units = "mg", time.units = "hours") %>%
  add.dosing(dosing.to = "centr",
           dose = dose_gcv,  # mg
           rate = dose_gcv/1, # infusion for 1h
           nbr.doses = 14,
           dosing.interval = 12,
           start.time = 0) %>%
  # 0-12h after the last dose
```

```
  add.sampling(seq(from=156,to=168,by=1))

# Perform simulation
# total number of subject: 1000
sim3 <- rxSolve(mod3,theta,ev3,omega=omega,sigma=sigma,nSub=1000)

# Concentration
pk_gcv <- sim3 %>%
  group_by(time) %>%
  # 10-90% Prediction interval
  summarise(medconc = median(cp),
            lowconc = quantile(cp,0.1),
            highconc = quantile(cp, 0.9)) %>%
  ungroup() %>%
  # time after last dose
  mutate(tald = time-156)

# make the margin smooth by lowess
l0.1 <- lowess(pk_gcv$tald, pk_gcv$lowconc, f=0.35)
l0.5 <- lowess(pk_gcv$tald, pk_gcv$medconc, f=0.35)
l0.9 <- lowess(pk_gcv$tald, pk_gcv$highconc, f=0.35)

df0.1 <- data.frame(tald=l0.1$x,lowconc=l0.1$y)
df0.5 <- data.frame(tald=l0.5$x,medconc=l0.5$y)
df0.9 <- data.frame(tald=l0.9$x,highconc=l0.9$y)

gcv_dat <- df0.1 %>%
  left_join(df0.5,by="tald") %>%
  left_join(df0.9,by="tald")

dat11 <- pk_gcv %>%
  mutate(study="Franck2021",
         drug="GCV",
         pop="infant")

# DOSE of VGCV(oral) input ------------------------------------------
---
dose_vgcv <- 10*BW # 10 mg/kg/12h for children

# Define event record
ev4 <- et(amount.units = "mg", time.units = "hours") %>%
  add.dosing(dosing.to = "depot",
             dose = dose_vgcv,  # mg
             nbr.doses = 14,
```

```
           dosing.interval = 12,
           start.time = 0) %>%
  # 0-12h after the last dose
  add.sampling(seq(from=156,to=168.33,by=1))

# Perform simulation
# total number of subject: 1000
sim4 <- rxSolve(mod3,theta,ev4,omega=omega,sigma=sigma,nSub=1000)

# Concentration
pk_vgcv <- sim4 %>%
  group_by(time) %>%
  # 10-90% Prediction interval
  summarise(medconc = median(cp),
          lowconc = quantile(cp,0.1),
          highconc = quantile(cp, 0.9)) %>%
  ungroup() %>%
  # time after last dose
  mutate(tald = time-156)

dat12 <- pk_vgcv %>%
  mutate(study="Franck2021",
        drug="VGCV",
        pop="infant")
```

Chen et al.(2021)

```
# 4.##Chen et al.(2021)## --------------------------------------------
----------
# Define model -------------------------------------------------------
------
# CL/F = 7.09 x (1 + CLcr/68.3 x 1.08) (L/h)
# V2/F = 10.8 (L)
# Q/F  = 3.98 (L/h)
# V3/F = 174 (L)
# Ka   = 0.23 (h-1)
# Tlag = 0.93 (h)

# BSV (CV%): CL/F = 27.2%, V2/F = 153%, Q/F = 63.1%, V3/F = 107%
# RUV: exponential.err = 42.9%
# CLcr:  creatinine clearance (mL/min), calculated by C-G formula

# Typical patient: adult(male),40 years old,BW=70kg,SCR=95umol/L
```

```r
# Dose: VGCV oral, 900 mg/12h (according to the drug label for
treatment)

set.seed(12345)
rxSetSeed(12345)

mod4 <- rxode2({
  CL   = TVCL*exp(eta.CL);
  V2   = TVV2*exp(eta.V2);
  Q    = TVQ*exp(eta.Q);
  V3   = TVV3*exp(eta.V3);
  KA   = TVKA;

  C2 = centr/V2;
  C3 = peri/V3;
  d/dt(depot) = -KA*depot;
  d/dt(centr) = KA*depot - CL*C2 - Q*C2 + Q*C3;
  d/dt(peri)  = Q*C2 - Q*C3;
  alag(depot) = TLAG;

  cp = C2*exp(expo.err.sd);
})

# Adults -------------------------------------------------------------
------
# Define typical patient: adults
BW <- 70   # kg
AGE <- 40  # years old
SCR <- 95/88.4 # mg/dL (1mg/dL = 88.4umol/L)
CLcr <- ((140-AGE)*BW)/(72*SCR) # ~90mL/min, calculated by C-G
formula


# Define fixed effect parameters
theta <- c(TVCL=7.09*(1 + (CLcr/68.3)*1.08),
         TVV2=10.8,
         TVQ =3.98,
         TVV3=174,
         TVKA=0.23,
         TLAG=0.93)

# Define between subject variability
omega <- lotri(eta.CL ~ 0.272^2, eta.V2 ~ 1.53^2,
             eta.Q ~ 0.631^2, eta.V3 ~ 1.07^2)
```

```r
# Define unexplained variability
sigma <- lotri(expo.err.sd ~ 0.429^2)


# DOSE of VGCV(oral) input -----------------------------------------
---
dose_vgcv <- 900 # 900 mg/12h for adults

# Define event record
ev1 <- et(amount.units = "mg", time.units = "hours") %>%
  add.dosing(dosing.to = "depot",
             dose = dose_vgcv,  # mg
             nbr.doses = 14,
             dosing.interval = 12,
             start.time = 0) %>%
  # 0-12h after the last dose
  add.sampling(seq(from=156,to=168.93,by=1))

# Perform simulation
# total number of subject: 1000
sim1 <- rxSolve(mod4,theta,ev1,omega=omega,sigma=sigma,nSub=1000)

# Concentration
pk_vgcv <- sim1 %>%
  group_by(time) %>%
  # 10-90% Prediction interval
  summarise(medconc = median(cp),
            lowconc = quantile(cp,0.1),
            highconc = quantile(cp, 0.9)) %>%
  ungroup() %>%
  # time after last dose
  mutate(tald = time-156)

dat13 <- pk_vgcv %>%
  mutate(study="Chen2021",
         drug="VGCV",
         pop="adult")
```

Li et al.(2021)

```r
# 5.##Li et al.(2021)## --------------------------------------------
--------
```

```r
# Define model -------------------------------------------------------
------
# CL = 5.23 x KF^0.92 × (BW/12)^1.02 (L/h)
# V = 11.35 × (BW/12)^0.8 (L)
# BSV (CV%): CL = 12.9%, V = 65.8%
# prop.err = 0.0823
# KF: kidney function (mL/min/1.73m2), KF=eGFR/(120mL/min/1.73m2); BW
(kg)

# Typical patient1: children(boys),10 years
old,HT=130cm,BW=30kg,SCR=70umol/L
# Typical patient2: infants(boys),1 year
old,HT=70cm,BW=10kg,SCR=50umol/L
# Dose: GCV iv, 5 mg/kg/12h, infusion=1h

set.seed(123456)
rxSetSeed(123456)

mod5 <- rxode2({
  CL   = TVCL*exp(eta.CL);
  V    = TVV*exp(eta.V);

  C = centr/V;
  d/dt(centr) = -CL*C;

  cp = C*(1 + prop.err.sd);
})


# Children -----------------------------------------------------------
------
# Define typical patient: children
BW <- 30   # kg
HT <- 130  # cm
SCR <- 70/88.4 # mg/dL (1mg/dL = 88.4umol/L)
AGE <- 10 # years
eGFR <- 0.68*(HT/SCR)-0.0008*(HT/SCR)^2+0.48*AGE-25.68 # ~ 70
mL/min/1.73 m2, calculated by Gao formula for male
KF <- eGFR/120 # mL/min/1.73 m2

# Define fixed effect parameters
theta <- c(TVCL=5.23*KF^0.92*(BW/12)^1.02,
         TVV=11.35*(BW/12)^0.8)
```

```
# Define between subject variability
omega <- lotri(eta.CL ~ 0.129^2, eta.V ~ 0.658^2)

# Define unexplained variability
sigma <- lotri(prop.err.sd ~ 0.0823^2)


# DOSE of GCV(iv) input ---------------------------------------------
---
dose_gcv <- 5*BW # 5 mg/kg/12h for children

# Define event record
ev1 <- et(amount.units = "mg", time.units = "hours") %>%
  add.dosing(dosing.to = "centr",
             dose = dose_gcv,  # mg
             rate = dose_gcv/1, # infusion for 1h
             nbr.doses = 14,
             dosing.interval = 12,
             start.time = 0) %>%
  # 0-12h after the last dose
  add.sampling(seq(from=156,to=168,by=1))

# Perform simulation
# total number of subject: 1000
sim1 <- rxSolve(mod5,theta,ev1,omega=omega,sigma=sigma,nSub=1000)

# Concentration
pk_gcv <- sim1 %>%
  group_by(time) %>%
  # 10-90% Prediction interval
  summarise(medconc = median(cp),
            lowconc = quantile(cp,0.1),
            highconc = quantile(cp, 0.9)) %>%
  ungroup() %>%
  # time after last dose
  mutate(tald = time-156)

dat14 <- pk_gcv %>%
  mutate(study="Li2021",
         drug="GCV",
         pop="child")

# Infants ------------------------------------------------------------
-----
```

```
# Define typical patient: children
BW <- 10   # kg
HT <- 70  # cm
SCR <- 50/88.4 # mg/dL (1mg/dL = 88.4umol/L)
AGE <- 1 # year
eGFR <- 0.68*(HT/SCR)-0.0008*(HT/SCR)^2+0.48*AGE-25.68 # ~ 46
mL/min/1.73 m2, calculated by Gao formula for male
KF <- eGFR/120 # mL/min/1.73 m2

# Define fixed effect parameters
theta <- c(TVCL=5.23*KF^0.92*(BW/12)^1.02,
         TVV=11.35*(BW/12)^0.8)

# Define between subject variability
omega <- lotri(eta.CL ~ 0.129^2, eta.V ~ 0.658^2)

# Define unexplained variability
sigma <- lotri(prop.err.sd ~ 0.0823^2)


# DOSE of GCV(iv) input ---------------------------------------------
---
dose_gcv <- 5*BW # 5 mg/kg/12h for children

# Define event record
ev2 <- et(amount.units = "mg", time.units = "hours") %>%
  add.dosing(dosing.to = "centr",
           dose = dose_gcv,  # mg
           rate = dose_gcv/1, # infusion for 1h
           nbr.doses = 14,
           dosing.interval = 12,
           start.time = 0) %>%
  # 0-12h after the last dose
  add.sampling(seq(from=156,to=168,by=1))

# Perform simulation
# total number of subject: 1000
sim2 <- rxSolve(mod5,theta,ev2,omega=omega,sigma=sigma,nSub=1000)

# Concentration
pk_gcv <- sim2 %>%
  group_by(time) %>%
  # 10-90% Prediction interval
  summarise(medconc = median(cp),
```

```
          lowconc = quantile(cp,0.1),
          highconc = quantile(cp, 0.9)) %>%
  ungroup() %>%
  # time after last dose
  mutate(tald = time-156)


dat15 <- pk_gcv %>%
  mutate(study="Li2021",
        drug="GCV",
        pop="infant")
```

Krens et al.(2020)

```
# 6.##Krens et al.(2020)## ------------------------------------------
-----------
# Define model ------------------------------------------------------
------
# CL = 2.3 x (CKD_EPI/65)^0.71 (L/h)
# V = 42 (L)
# BSV (CV%): CL = 47%, V = 80%
# prop.err = 0.43
# CKD_EPI: eGFR calculated by CKD-EPI equation (mL/min/1.73m2)
# GFR=141*((SCR/0.9)^-1.209)*0.993^AGE=141*(((95/88.4)/0.9)^-
1.209)*0.993^40=85.91mL/min/1.73m2

# Typical patient: adult(male),40 years
old,BW=70kg,SCR=95umol/L,CKD_EPI=86mL/min/1.73m2
# Dose: GCV iv, 5 mg/kg/12h,infusion=1h

set.seed(12345)
rxSetSeed(12345)

mod6 <- rxode2({
  CL   = TVCL*exp(eta.CL);
  V    = TVV*exp(eta.V);

  C = centr/V;
  d/dt(centr) = -CL*C;

  cp = C*(1 + prop.err.sd);
})
```

```r
# Adults -------------------------------------------------------------
------
# Define typical patient: adults
BW <- 70   # kg
AGE <- 40  # years old
SCR <- 95/88.4 # mg/dL (1mg/dL = 88.4umol/L)
CKD_EPI <- 141*((SCR/0.9)^-1.209)*0.993^AGE # mL/min/1.73 m2

# Define fixed effect parameters
theta <- c(TVCL=2.3*(CKD_EPI/65)^0.71,
         TVV=42)

# Define between subject variability
omega <- lotri(eta.CL ~ 0.47^2, eta.V ~ 0.8^2)

# Define unexplained variability
sigma <- lotri(prop.err.sd ~ 0.43^2)

# DOSE of GCV(iv) input --------------------------------------------
---
dose_gcv <- 5*BW # 5 mg/kg/12h for adults

# Define event record
ev1 <- et(amount.units = "mg", time.units = "hours") %>%
  add.dosing(dosing.to = "centr",
           dose = dose_gcv,  # mg
           rate = dose_gcv/1, # infusion for 1h
           nbr.doses = 14,
           dosing.interval = 12,
           start.time = 0) %>%
  # 0-12h after the last dose
  add.sampling(seq(from=156,to=168,by=1))

# Perform simulation
# total number of subject: 1000
sim1 <- rxSolve(mod6,theta,ev1,omega=omega,sigma=sigma,nSub=1000)

# Concentration
pk_gcv <- sim1 %>%
  group_by(time) %>%
  # 10-90% Prediction interval
  summarise(medconc = median(cp),
          lowconc = quantile(cp,0.1),
          highconc = quantile(cp, 0.9)) %>%
```

```
  ungroup() %>%
  # time after last dose
  mutate(tald = time-156)


dat16 <- pk_gcv %>%
  mutate(study="Krens2020",
         drug="GCV",
         pop="adult")
```

Facchin et al.(2019)

```
# 7.##Facchin et al.(2019)## ---------------------------------------
-------------
# Define model -----------------------------------------------------
------
# CL/F = 9.07 x (SCR/72.5)^-0.768 x BSA^1.31 x 1.15^GENDER (L/h)
# V2/F = 45 x BSA^1.28 x 1.14^GENDER (L)
# Q/F  = 1.46 (L/h)
# V3/F = 18.5 (L)
# Ka   = 6.96 (h-1)
# Tlag = 0.86 (h)

# BSV (CV%): CL/F = 16.0%, V2/F = 9.3%, V3/F = 54.6%, Ka = 59.2%
# IOV (%): IOV.CL = 14.4%, IOV.V3 = 77.2%, IOV.KA = 111.4%
# RUV: prop.err = 23.5%
# SCR: serum creatinine level (umol/L); BSA: body surface area (m2);
# GENDER: 1 for male and 0 for female


# 1.Typical patient: children(boys),10 years
old,BW=30kg,HT=130cm,SCR=70umol/L
# Dose: VGCV oral, 10 mg/kg/12h

# 2.Typical patient: adults(male),40 years
old,BW=70kg,HT=170cm,SCR=95umol/L
# Dose: VGCV oral, 900 mg/12h (according to the drug label for
treatment)

set.seed(123456)
rxSetSeed(123456)

mod7 <- rxode2({
```

```
  CL   = TVCL*exp(eta.CL + iov.CL);
  V2   = TVV2*exp(eta.V2);
  Q    = TVQ;
  V3   = TVV3*exp(eta.V3 + iov.V3);
  KA   = TVKA*exp(eta.KA + iov.KA);

  C2 = centr/V2;
  C3 = peri/V3;
  d/dt(depot) = -KA*depot;
  d/dt(centr) = KA*depot - CL*C2 - Q*C2 + Q*C3;
  d/dt(peri)  = Q*C2 - Q*C3;
  alag(depot) = TLAG;

  cp = C2*(1 + prop.err.sd);
})


# Children ---------------------------------------------
# Define typical patient: children
BW <- 30   # kg
HT <- 130  # cm
AGE <- 10  # years old
SCR <- 70 # umol/L
BSA <- sqrt((HT*BW)/3600) # m2
GENDER <- 1 # 1 for male and 0 for female

# Define fixed effect parameters
theta <- c(TVCL=9.07*((SCR/72.5)^(-0.768))*(BSA^1.31)*(1.15^GENDER),
         TVV2=45*(BSA^1.28)*(1.14^GENDER),
         TVQ =1.46,
         TVV3=18.5,
         TVKA=6.96,
         TLAG=0.86)

# Define between subject variability
omega <- lotri(eta.CL ~ 0.16^2, eta.V2 ~ 0.093^2,
             eta.V3 ~ 0.546^2, eta.KA ~ 0.592^2,
             iov.CL ~ 0.144^2, iov.V3 ~ 0.772^2, iov.KA ~ 1.114^2)

# Define unexplained variability
sigma <- lotri(prop.err.sd ~ 0.235^2)


dose_vgcv <- 10*BW # 10 mg/kg/12h for children
```

```
# Define event record
ev1 <- et(amount.units = "mg", time.units = "hours") %>%
  add.dosing(dosing.to = "depot",
             dose = dose_vgcv,  # mg
             nbr.doses = 14,
             dosing.interval = 12,
             start.time = 0) %>%
  # 0-12h after the last dose
  add.sampling(seq(from=156,to=168.86,by=1))

# Perform simulation
# total number of subject: 1000
sim1 <- rxSolve(mod7,theta,ev1,omega=omega,sigma=sigma,nSub=1000)


# Concentration
pk_vgcv <- sim1 %>%
  group_by(time) %>%
  # 10-90% Prediction interval
  summarise(medconc = median(cp),
            lowconc = quantile(cp,0.1),
            highconc = quantile(cp, 0.9)) %>%
  ungroup() %>%
  # time after last dose
  mutate(tald = time-156)

dat17 <- pk_vgcv %>%
  mutate(study="Facchin2019",
         drug="VGCV",
         pop="child")

# Adults ---------------------------------------------
# Define typical patient: adults
BW <- 70   # kg
HT <- 170  # cm
AGE <- 40  # years old
SCR <- 95 # mg/dL (1mg/dL = 88.4umol/L)
BSA <- sqrt((HT*BW)/3600) # m2
GENDER <- 1 # 1 for male and 0 for female


# Define fixed effect parameters
theta <- c(TVCL=9.07*((SCR/72.5)^(-0.768))*(BSA^1.31)*(1.15^GENDER),
```

```
          TVV2=45*(BSA^1.28)*(1.14^GENDER),
          TVQ =1.46,
          TVV3=18.5,
          TVKA=6.96,
          TLAG=0.86)

# Define between subject variability
omega <- lotri(eta.CL ~ 0.16^2, eta.V2 ~ 0.093^2,
             eta.V3 ~ 0.546^2, eta.KA ~ 0.592^2,
             iov.CL ~ 0.144^2, iov.V3 ~ 0.772^2, iov.KA ~ 1.114^2)

# Define unexplained variability
sigma <- lotri(prop.err.sd ~ 0.235^2)


dose_vgcv <- 900 # 900 mg/12h for adults

# Define event record
ev2 <- et(amount.units = "mg", time.units = "hours") %>%
  add.dosing(dosing.to = "depot",
           dose = dose_vgcv,  # mg
           nbr.doses = 14,
           dosing.interval = 12,
           start.time = 0) %>%
  # 0-12h after the last dose
  add.sampling(seq(from=156,to=168.86,by=1))

# Perform simulation
# total number of subject: 1000
sim2 <- rxSolve(mod7,theta,ev2,omega=omega,sigma=sigma,nSub=1000)


# Concentration
pk_vgcv <- sim2 %>%
  group_by(time) %>%
  # 10-90% Prediction interval
  summarise(medconc = median(cp),
          lowconc = quantile(cp,0.1),
          highconc = quantile(cp, 0.9)) %>%
  ungroup() %>%
  # time after last dose
  mutate(tald = time-156)

dat18 <- pk_vgcv %>%
```

```
  mutate(study="Facchin2019",
         drug="VGCV",
         pop="adult")
```

Horvatits et al.(2014)

```
# 8.##Horvatits et al.(2014)## --------------------------------------
---------------
# Define model -------------------------------------------------------
------
# CL = 2.2 (L/h)
# V1 = 32.4 (L)
# Q  = 16.8 (L/h)
# V2 = 33.5 (L)
# BSV (CV%): CL = 61.5%, V1 = 33.6%, Q = 34.7%, V2 = 60.6%
# prop.err = 7.22%

# Typical patient: adults(male),40 years old,BW=70kg,SCR=95umol/L
# Dose: GCV iv, 5 mg/kg/12h, infusion=1h

set.seed(12345)
rxSetSeed(12345)

mod8 <- rxode2({
  CL  = TVCL*exp(eta.CL);
  V1  = TVV1*exp(eta.V1);
  Q   = TVQ*exp(eta.Q);
  V2  = TVV2*exp(eta.V2);

  C1 = centr/V1;
  C2 = peri/V2
  d/dt(centr) = - CL*C1 - Q*C1 + Q*C2;
  d/dt(peri)  = Q*C1 - Q*C2;

  cp = C1*(1 + prop.err.sd);
})


# Adults -------------------------------------------------------------
------
# Define typical patient: adults
BW <- 70   # kg
AGE <- 40  # years old
SCR <- 95/88.4 # mg/dL (1mg/dL = 88.4umol/L)
```

```r
# Define fixed effect parameters
theta <- c(TVCL=2.2, TVV1=32.4, TVQ=16.8, TVV2=33.5)

# Define between subject variability
omega <- lotri(eta.CL ~ 0.615^2, eta.V1 ~ 0.336^2,
               eta.Q ~ 0.347^2, eta.V2 ~ 0.606^2)

# Define unexplained variability
sigma <- lotri(prop.err.sd ~ 0.0722^2)


# DOSE of GCV(iv) input -----------------------------------------------
---
dose_gcv <- 5*BW # 5 mg/kg/12h for adults

# Define event record
ev1 <- et(amount.units = "mg", time.units = "hours") %>%
  add.dosing(dosing.to = "centr",
             dose = dose_gcv,  # mg
             rate = dose_gcv/1, # infusion for 1h
             nbr.doses = 14,
             dosing.interval = 12,
             start.time = 0) %>%
  # 0-12h after the last dose
  add.sampling(seq(from=156,to=168,by=1))

# Perform simulation
# total number of subject: 1000
sim1 <- rxSolve(mod8,theta,ev1,omega=omega,sigma=sigma,nSub=1000)

# Concentration
pk_gcv <- sim1 %>%
  group_by(time) %>%
  # 10-90% Prediction interval
  summarise(medconc = median(cp),
            lowconc = quantile(cp,0.1),
            highconc = quantile(cp, 0.9)) %>%
  ungroup() %>%
  # time after last dose
  mutate(tald = time-156)


dat19 <- pk_gcv %>%
```

```
  mutate(study="Horvatits2014",
         drug="GCV",
         pop="adult")
```

Vezina et al.(2014)

```
# 9.##Vezina et al.(2014)## -----------------------------------------
------------
# Define model -------------------------------------------------------
------
# CL/F = 14.5 × ((CLcr/60) x (70/BW))^0.492 x (BW/70)^0.75 (L/h)
# V2/F = 87.5 x (BW/70) (L)
# Q/F  = 4.8 x (BW/70)^0.75 (L/h)
# V3/F = 42.6 x (BW/70) (L)
# Ka   = 3 (h-1)
# Tlag = 0.5 (h)

# BSV (CV%): CL/F = 33.5%
# RUV: prop.err = 32.7%
# CLcr: creatinine clearance, calculated by C-G method for adults and
Schwartz formula for children


# 1.Typical patient: children(boys),10 years
old,BW=30kg,HT=130cm,SCR=70umol/L
# Dose: VGCV oral, 10 mg/kg/12h

# 2.Typical patient: adults(male),40 years
old,BW=70kg,HT=170cm,SCR=95umol/L
# Dose: VGCV oral, 900 mg/12h (according to the drug label for
treatment)

# 3.Typical patient: infants(boys),1 year
old,BW=10kg,HT=70cm,SCR=50umol/L
# Dose: VGCV oral, 10 mg/kg/12h

set.seed(123456)
rxSetSeed(123456)

mod9 <- rxode2({
  CL   = TVCL*exp(eta.CL);
  V2   = TVV2;
  Q    = TVQ;
```

```
  V3   = TVV3;
  KA   = TVKA;

  C2 = centr/V2;
  C3 = peri/V3;
  d/dt(depot) = -KA*depot;
  d/dt(centr) = KA*depot - CL*C2 - Q*C2 + Q*C3;
  d/dt(peri)  = Q*C2 - Q*C3;
  alag(depot) = TLAG;

  cp = C2*(1 + prop.err.sd);
})


# Children ---------------------------------------------
# Define typical patient: children
BW <- 30   # kg
HT <- 130  # cm
AGE <- 10  # years old
SCR <- 70/88.4 # mg/dL (1mg/dL = 88.4umol/L)
k <- 0.55 # k = 0.55 for boys aged 2 years to less than 13 years
CLcr <- k*HT/SCR # ~90mL/min/1.73 m2, calculated by modified Schwartz
formula for children


# Define fixed effect parameters
theta <- c(TVCL=14.5*((CLcr/60)*(70/BW))^0.492*(BW/70)^0.75,
         TVV2=87.5*(BW/70),
         TVQ =4.8*(BW/70)^0.75 ,
         TVV3=42.6*(BW/70),
         TVKA=3,
         TLAG=0.5)

# Define between subject variability
omega <- lotri(eta.CL ~ 0.335^2)

# Define unexplained variability
sigma <- lotri(prop.err.sd ~ 0.327^2)

dose_vgcv <- 10*BW # 10 mg/kg/12h for children

# Define event record
ev1 <- et(amount.units = "mg", time.units = "hours") %>%
  add.dosing(dosing.to = "depot",
```

```
           dose = dose_vgcv,  # mg
           nbr.doses = 14,
           dosing.interval = 12,
           start.time = 0) %>%
  # 0-12h after the last dose
  add.sampling(seq(from=156,to=168.5,by=1))

# Perform simulation
# total number of subject: 1000
sim1 <- rxSolve(mod9,theta,ev1,omega=omega,sigma=sigma,nSub=1000)


# Concentration
pk_vgcv <- sim1 %>%
  group_by(time) %>%
  # 10-90% Prediction interval
  summarise(medconc = median(cp),
          lowconc = quantile(cp,0.1),
          highconc = quantile(cp, 0.9)) %>%
  ungroup() %>%
  # time after last dose
  mutate(tald = time-156)


dat20 <- pk_vgcv %>%
  mutate(study="Vezina2014",
        drug="VGCV",
        pop="child")

# Adults ---------------------------------------------
# Define typical patient: adults
BW <- 70   # kg
HT <- 170  # cm
AGE <- 40  # years old
SCR <- 95/88.4 # mg/dL (1mg/dL = 88.4umol/L)
CLcr <- ((140-AGE)*BW)/(72*SCR) # ~90mL/min, calculated by C-G
formula for adults

# Define fixed effect parameters
theta <- c(TVCL=14.5*((CLcr/60)*(70/BW))^0.492*(BW/70)^0.75,
         TVV2=87.5*(BW/70),
         TVQ =4.8*(BW/70)^0.75 ,
         TVV3=42.6*(BW/70),
         TVKA=3,
```

```
         TLAG=0.5)

# Define between subject variability
omega <- lotri(eta.CL ~ 0.335^2)

# Define unexplained variability
sigma <- lotri(prop.err.sd ~ 0.327^2)

dose_vgcv <- 900 # 900 mg/12h for adults

# Define event record
ev2 <- et(amount.units = "mg", time.units = "hours") %>%
  add.dosing(dosing.to = "depot",
             dose = dose_vgcv,  # mg
             nbr.doses = 14,
             dosing.interval = 12,
             start.time = 0) %>%
  # 0-12h after the last dose
  add.sampling(seq(from=156,to=168.5,by=1))

# Perform simulation
# total number of subject: 1000
sim2 <- rxSolve(mod9,theta,ev2,omega=omega,sigma=sigma,nSub=1000)


# Concentration
pk_vgcv <- sim2 %>%
  group_by(time) %>%
  # 10-90% Prediction interval
  summarise(medconc = median(cp),
            lowconc = quantile(cp,0.1),
            highconc = quantile(cp, 0.9)) %>%
  ungroup() %>%
  # time after last dose
  mutate(tald = time-156)


dat21 <- pk_vgcv %>%
  mutate(study="Vezina2014",
         drug="VGCV",
         pop="adult")

# Infants ---------------------------------------------
# Define typical patient: infants
```

```
BW <- 10   # kg
HT <- 70  # cm
AGE <- 1  # years old
SCR <- 50/88.4 # mg/dL (1mg/dL = 88.4umol/L)
k <- 0.45 # k = 0.45 for subjects aged less than 2 years
CLcr <- k*HT/SCR # ~ 55.7 mL/min/1.73 m2, calculated by modified
Schwartz formula for children

# Define fixed effect parameters
theta <- c(TVCL=14.5*((CLcr/60)*(70/BW))^0.492*(BW/70)^0.75,
          TVV2=87.5*(BW/70),
          TVQ =4.8*(BW/70)^0.75 ,
          TVV3=42.6*(BW/70),
          TVKA=3,
          TLAG=0.5)

# Define between subject variability
omega <- lotri(eta.CL ~ 0.335^2)

# Define unexplained variability
sigma <- lotri(prop.err.sd ~ 0.327^2)

dose_vgcv <- 10*BW # 10 mg/kg/12h for children

# Define event record
ev3 <- et(amount.units = "mg", time.units = "hours") %>%
  add.dosing(dosing.to = "depot",
           dose = dose_vgcv,  # mg
           nbr.doses = 14,
           dosing.interval = 12,
           start.time = 0) %>%
  # 0-12h after the last dose
  add.sampling(seq(from=156,to=168.5,by=1))

# Perform simulation
# total number of subject: 1000
sim3 <- rxSolve(mod9,theta,ev3,omega=omega,sigma=sigma,nSub=1000)

# Concentration
pk_vgcv <- sim3 %>%
  group_by(time) %>%
  # 10-90% Prediction interval
  summarise(medconc = median(cp),
          lowconc = quantile(cp,0.1),
```

```
          highconc = quantile(cp, 0.9)) %>%
  ungroup() %>%
  # time after last dose
  mutate(tald = time-156)


dat22 <- pk_vgcv %>%
  mutate(study="Vezina2014",
         drug="VGCV",
         pop="infant")
```

Vezina et al.(2010)

```
# 10.##Vezina et al.(2010)## ----------------------------------------
-------------
# Define model --------------------------------------------------------
------
# CL/F = 7.33 (L/h)
# V/F  = 35.1 (L)
# Ka   = 0.85 (h-1)

# BSV (CV%): CL/F = 36.3%, V/F = 41.4%, Ka = 74.3%
# RUV: prop.err = 33.5%

# Typical patient: children (boys),10 years
old,BW=30kg,HT=130cm,SCR=70umol/L
# Dose: VGCV oral, 10 mg/kg/12h

set.seed(1234)
rxSetSeed(1234)

mod10 <- rxode2({
  CL   = TVCL*exp(eta.CL);
  V    = TVV*exp(eta.V);
  KA   = TVKA*exp(eta.KA);

  C = centr/V;
  d/dt(depot) = -KA*depot;
  d/dt(centr) = KA*depot - CL*C;

  cp = C*(1 + prop.err.sd);
})
```

```
# Children ------------------------------------------------------------
------
# Define typical patient: children
BW <- 30   # kg
AGE <- 10  # years old

# Define fixed effect parameters
theta <- c(TVCL=7.33,
         TVV =35.1,
         TVKA=0.85)

# Define between subject variability
omega <- lotri(eta.CL ~ 0.363^2, eta.V ~ 0.414^2, eta.KA ~ 0.743^2)

# Define unexplained variability
sigma <- lotri(prop.err.sd ~ 0.335^2)


# DOSE of VGCV(oral) input ------------------------------------------
---
dose_vgcv <- 10*BW # 10 mg/kg/12h for Children

# Define event record
ev1 <- et(amount.units = "mg", time.units = "hours") %>%
  add.dosing(dosing.to = "depot",
           dose = dose_vgcv,  # mg
           nbr.doses = 14,
           dosing.interval = 12,
           start.time = 0) %>%
  # 0-12h after the last dose
  add.sampling(seq(from=156,to=168,by=1))

# Perform simulation
# total number of subject: 1000
sim1 <- rxSolve(mod10,theta,ev1,omega=omega,sigma=sigma,nSub=1000)


# Concentration
pk_vgcv <- sim1 %>%
  group_by(time) %>%
  # 10-90% Prediction interval
  summarise(medconc = median(cp),
          lowconc = quantile(cp,0.1),
```

```
          highconc = quantile(cp, 0.9)) %>%
  ungroup() %>%
  # time after last dose
  mutate(tald = time-156)

# make the margin smooth by lowess: can't make it
l0.1 <- lowess(pk_vgcv$tald, pk_vgcv$lowconc, f=0.2)
l0.5 <- lowess(pk_vgcv$tald, pk_vgcv$medconc, f=0.2)
l0.9 <- lowess(pk_vgcv$tald, pk_vgcv$highconc, f=0.2)

df0.1 <- data.frame(tald=l0.1$x,lowconc=l0.1$y)
df0.5 <- data.frame(tald=l0.5$x,medconc=l0.5$y)
df0.9 <- data.frame(tald=l0.9$x,highconc=l0.9$y)

vgcv_dat <- df0.1 %>%
  left_join(df0.5,by="tald") %>%
  left_join(df0.9,by="tald")

dat23 <- vgcv_dat %>%
  mutate(study="Vezina2010",
         drug="VGCV",
         pop="child")
```

Caldés et al.(2009)

```
# 11.##Caldés et al.(2009)## ----------------------------------------
-------------
# Define model -------------------------------------------------------
------
# CL   = 7.49 × (CLcr/57)  (L/h)
# V2   = 31.9  (L)
# Q    = 10.2  (L/h)
# V3   = 32  (L)
# Ka   = 0.895  (h-1)
# F1   = 0.825  ; TVF1=exp(1.5506)/(1+exp(1.5506))=0.825,
logitF=1.5506
# Tlag = 0.382  (h)
# BSV (CV%): CL = 32.7%, V2 = 47.6%, Ka = 68.1%, F1 = 22.1%
# prop.err = 14.3%, add.err =  0.465 ug/mL = 0.465 mg/L
# CLcr: creatinine clearance (mL/min), calculated by C-G formula

# Typical patient: adult (male),40 years
old,BW=70kg,HT=170cm,SCR=95umol/L
```

```
# Dose:GCV iv: 5 mg/kg/12h, infusion=1h
# VGCV oral, 900 mg/12h (according to the drug label for treatment)

set.seed(123456)
rxSetSeed(123456)

mod11 <- rxode2({
  CL   = TVCL*exp(eta.CL);
  V2   = TVV2*exp(eta.V2);
  Q    = TVQ;
  V3   = TVV3;
  KA   = TVKA*exp(eta.KA);
  F1   = exp(logitF + eta.F1)/(1 + exp(logitF + eta.F1));

  C2 = centr/V2;
  C3 = peri/V3;
  d/dt(depot) = -KA*depot;
  d/dt(centr) =  F1*KA*depot - CL*C2 - Q*C2 + Q*C3;
  d/dt(peri)  = Q*C2 - Q*C3;
  alag(depot) = TLAG;

  cp = C2*(1 + prop.err.sd) + add.err.sd;
})


# Adults -------------------------------------------------------------
------
# Define typical patient: adults
BW <- 70   # kg
HT <- 170  # cm
AGE <- 40  # years old
SCR <- 95/88.4 # mg/dL (1mg/dL = 88.4umol/L)
CLcr <- (140-AGE)*BW/(72*SCR) # mL/min, C-G formula

# Define fixed effect parameters
theta <- c(TVCL   = 7.49*(CLcr/57),
         TVV2   = 31.9,
         TVQ    = 10.2,
         TVV3   = 32,
         TVKA   = 0.895,
         logitF = 1.5506,
         TLAG   = 0.382)

# Define between subject variability
```

```
omega <- lotri(eta.CL ~ 0.327^2, eta.V2 ~ 0.476^2,
            eta.KA ~ 0.681^2, eta.F1 ~ 0.221^2)

# Define unexplained variability
sigma <- lotri(prop.err.sd ~ 0.143^2, add.err.sd ~ 0.465^2)


# DOSE of GCV(iv) input ---------------------------------------------
---
dose_gcv <- 5*BW # 5 mg/kg/12h for adults

# Define event record
ev1 <- et(amount.units = "mg", time.units = "hours") %>%
  add.dosing(dosing.to = "centr",
             dose = dose_gcv,  # mg
             rate = dose_gcv/1, # infusion for 1h
             nbr.doses = 14,
             dosing.interval = 12,
             start.time = 0) %>%
  # 0-12h after the last dose
  add.sampling(seq(from=156,to=168,by=1))

# Perform simulation
# total number of subject: 1000
sim1 <- rxSolve(mod11,theta,ev1,omega=omega,sigma=sigma,nSub=1000)

# Concentration
pk_gcv <- sim1 %>%
  group_by(time) %>%
  # 10-90% Prediction interval
  summarise(medconc = median(cp),
            lowconc = quantile(cp,0.1),
            highconc = quantile(cp, 0.9)) %>%
  ungroup() %>%
  # time after last dose
  mutate(tald = time-156)

dat24 <- pk_gcv %>%
  mutate(study="Caldés2009",
         drug="GCV",
         pop="adult")

# DOSE of VGCV(oral) input ------------------------------------------
---
```

```
dose_vgcv <- 900*0.72 # 900 mg/12h × 0.72 (the ratio between the
molecular weights of GCV and VGCV)

# Define event record
ev2 <- et(amount.units = "mg", time.units = "hours") %>%
  add.dosing(dosing.to = "depot",
             dose = dose_vgcv,  # mg
             nbr.doses = 14,
             dosing.interval = 12,
             start.time = 0) %>%
  # 0-12h after the last dose
  add.sampling(seq(from=156,to=168,by=1))

# Perform simulation
# total number of subject: 1000
sim2 <- rxSolve(mod11,theta,ev2,omega=omega,sigma=sigma,nSub=1000)

# Concentration
pk_vgcv <- sim2 %>%
  group_by(time) %>%
  # 10-90% Prediction interval
  summarise(medconc = median(cp),
            lowconc = quantile(cp,0.1),
            highconc = quantile(cp, 0.9)) %>%
  ungroup() %>%
  # time after last dose
  mutate(tald = time-156)

dat25 <- pk_vgcv %>%
  mutate(study="Caldés2009",
         drug="VGCV",
         pop="adult")
```

Perrottet et al.(2009)

```
# 12. ##Perrottet et al.(2009)## ------------------------------------
-----------------
# Define model -------------------------------------------------------
------
# CL = θGraftType x GFR_MDRD x 1.21^sex (L/h)
# V2 = 24 x (BW/70) x 0.78^sex (L)
# Q  = 4.1 (L/h)
# V3 = 22 (L)
```

```
# Ka = 0.56 (h-1)
# F  = 0.6

# BSV (CV%): CL = 26%, V2 = 20%
# IOV (%): IOV.CL = 12%
# RUV: prop.err = 21%
# θGraftType:  θkidney=1.68, θheart=0.86, θlung/liver=1.17
# GFR_MDRD: : four-variable modification of diet in renal disease
eGFR (L/h)
# sex: for male, sex=0 and for female, sex=1

# Typical patient: adult (male),40 years
old,BW=70kg,HT=170cm,SCR=95umol/L
# Dose: GCV iv, 5 mg/kg/12h, infusion=1h
# VGCV oral, 900 mg/12h (according to the drug label for treatment)

set.seed(123456)
rxSetSeed(123456)

mod12 <- rxode2({
  CL   = TVCL*exp(eta.CL + iov.CL);
  V2   = TVV2*exp(eta.V2);
  Q    = TVQ;
  V3   = TVV3;
  KA   = TVKA;
  F1   = TVF1;

  C2 = centr/V2;
  C3 = peri/V3;
  d/dt(depot)  = -KA*depot;
  d/dt(centr)  = F1*KA*depot - CL*C2 - Q*C2 + Q*C3;
  d/dt(peri)   = Q*C2 - Q*C3;

  cp = C2*(1 + prop.err.sd);
})


# Adults -------------------------------------------------------------
------
# Define typical patient: adults
BW <- 70   # kg
HT <- 170  # cm
BSA <- sqrt(BW*HT/3600) # m2
AGE <- 40  # years old
```

```r
SCR <- 95/88.4 # mg/dL (1mg/dL = 88.4umol/L)
sex <- 0 # 0 for male and 1 for female
# GFR_MDRD=175*(SCR^-1.154)*(AGE^-0.203) *0.742 (if female)
GFR_MDRD <- 175*(SCR^-1.154)*(AGE^-0.203) # ~ 76 mL/min/1.73m2
GFR_MDRD <- (GFR_MDRD/(1000/60))*(BSA/1.73) # ~ 4.8 L/h

# values of θGraftType
θkidney <- 1.68
θheart <- 0.86
θlung_liver <- 1.17


# 12.1 Kidney transplant -----------------------------------------
-----------
# Define fixed effect parameters
theta <- c(TVCL=θkidney*GFR_MDRD*(1.21^sex) ,
         TVV2=24*(BW/70)*(0.78^sex),
         TVQ =4.1,
         TVV3=22,
         TVKA=0.56,
         TVF1=0.6)

# Define between subject variability
omega <- lotri(eta.CL ~ 0.26^2, eta.V2 ~ 0.2^2,
             iov.CL ~ 0.12^2)

# Define unexplained variability
sigma <- lotri(prop.err.sd ~ 0.21^2)


# DOSE of GCV(iv) input ----------------------------------------------
---
dose_gcv <- 5*BW # 5 mg/kg/12h for adults

# Define event record
ev1 <- et(amount.units = "mg", time.units = "hours") %>%
  add.dosing(dosing.to = "centr",
           dose = dose_gcv,  # mg
           rate = dose_gcv/1, # infusion for 1h
           nbr.doses = 14,
           dosing.interval = 12,
           start.time = 0) %>%
  # 0-12h after the last dose
  add.sampling(seq(from=156,to=168,by=1))
```

```
# Perform simulation
# total number of subject: 1000
sim1 <- rxSolve(mod12,theta,ev1,omega=omega,sigma=sigma,nSub=1000)

# Concentration
pk_gcv <- sim1 %>%
  group_by(time) %>%
  # 10-90% Prediction interval
  summarise(medconc = median(cp),
           lowconc = quantile(cp,0.1),
           highconc = quantile(cp, 0.9)) %>%
  ungroup() %>%
  # time after last dose
  mutate(tald = time-156)

dat26 <- pk_gcv %>%
  mutate(study="Perrottet2009_kidney",
        drug="GCV",
        pop="adult")

# plot adults VGCV(oral) conc vs time -------------------------------
--------------
dose_vgcv <- 900 # 900 mg/12h for adults

# Define event record
ev2 <- et(amount.units = "mg", time.units = "hours") %>%
  add.dosing(dosing.to = "depot",
            dose = dose_vgcv,  # mg
            nbr.doses = 14,
            dosing.interval = 12,
            start.time = 0) %>%
  # 0-12h after the last dose
  add.sampling(seq(from=156,to=168,by=1))

# Perform simulation
# total number of subject: 1000
sim2 <- rxSolve(mod12,theta,ev2,omega=omega,sigma=sigma,nSub=1000)


# Concentration
pk_vgcv <- sim2 %>%
  group_by(time) %>%
  # 10-90% Prediction interval
```

```
  summarise(medconc = median(cp),
            lowconc = quantile(cp,0.1),
            highconc = quantile(cp, 0.9)) %>%
  ungroup() %>%
  # time after last dose
  mutate(tald = time-156)

dat27 <- pk_vgcv %>%
  mutate(study="Perrottet2009_kidney",
         drug="VGCV",
         pop="adult")

# 12.2 heart transplant -----------------------------------------------
----------
# Define fixed effect parameters
theta <- c(TVCL=θheart*GFR_MDRD*(1.21^sex) ,
           TVV2=24*(BW/70)*(0.78^sex),
           TVQ =4.1,
           TVV3=22,
           TVKA=0.56,
           TVF1  =0.6)

# Define between subject variability
omega <- lotri(eta.CL ~ 0.26^2, eta.V2 ~ 0.2^2,
               iov.CL ~ 0.12^2)

# Define unexplained variability
sigma <- lotri(prop.err.sd ~ 0.21^2)


# DOSE of GCV(iv) input ----------------------------------------------
---
dose_gcv <- 5*BW # 5 mg/kg/12h for adults

# Define event record
ev3 <- et(amount.units = "mg", time.units = "hours") %>%
  add.dosing(dosing.to = "centr",
             dose = dose_gcv,  # mg
             rate = dose_gcv/1, # infusion for 1h
             nbr.doses = 14,
             dosing.interval = 12,
             start.time = 0) %>%
  # 0-12h after the last dose
  add.sampling(seq(from=156,to=168,by=1))
```

```
# Perform simulation
# total number of subject: 1000
sim3 <- rxSolve(mod12,theta,ev3,omega=omega,sigma=sigma,nSub=1000)

# Concentration
pk_gcv <- sim3 %>%
  group_by(time) %>%
  # 10-90% Prediction interval
  summarise(medconc = median(cp),
            lowconc = quantile(cp,0.1),
            highconc = quantile(cp, 0.9)) %>%
  ungroup() %>%
  # time after last dose
  mutate(tald = time-156)

dat28 <- pk_gcv %>%
  mutate(study="Perrottet2009_heart",
         drug="GCV",
         pop="adult")

# plot adults VGCV(oral) conc vs time -------------------------------
--------------
dose_vgcv <- 900 # 900 mg/12h for adults

# Define event record
ev4 <- et(amount.units = "mg", time.units = "hours") %>%
  add.dosing(dosing.to = "depot",
             dose = dose_vgcv,  # mg
             nbr.doses = 14,
             dosing.interval = 12,
             start.time = 0) %>%
  # 0-12h after the last dose
  add.sampling(seq(from=156,to=168,by=1))

# Perform simulation
# total number of subject: 1000
sim4 <- rxSolve(mod12,theta,ev4,omega=omega,sigma=sigma,nSub=1000)


# Concentration
pk_vgcv <- sim4 %>%
  group_by(time) %>%
  # 10-90% Prediction interval
```

```
  summarise(medconc = median(cp),
            lowconc = quantile(cp,0.1),
            highconc = quantile(cp, 0.9)) %>%
  ungroup() %>%
  # time after last dose
  mutate(tald = time-156)


dat29 <- pk_vgcv %>%
  mutate(study="Perrottet2009_heart",
         drug="VGCV",
         pop="adult")

# 12.3 lung_liver transplant -----------------------------------------
---------------
# Define fixed effect parameters
theta <- c(TVCL=θlung_liver*GFR_MDRD*(1.21^sex) ,
         TVV2=24*(BW/70)*(0.78^sex),
         TVQ =4.1,
         TVV3=22,
         TVKA=0.56,
         TVF1  =0.6)

# Define between subject variability
omega <- lotri(eta.CL ~ 0.26^2, eta.V2 ~ 0.2^2,
            iov.CL ~ 0.12^2)

# Define unexplained variability
sigma <- lotri(prop.err.sd ~ 0.21^2)


# DOSE of GCV(iv) input ----------------------------------------------
---
dose_gcv <- 5*BW # 5 mg/kg/12h for adults

# Define event record
ev5 <- et(amount.units = "mg", time.units = "hours") %>%
  add.dosing(dosing.to = "centr",
           dose = dose_gcv,  # mg
           rate = dose_gcv/1, # infusion for 1h
           nbr.doses = 14,
           dosing.interval = 12,
           start.time = 0) %>%
  # 0-12h after the last dose
```

```
  add.sampling(seq(from=156,to=168,by=1))

# Perform simulation
# total number of subject: 1000
sim5 <- rxSolve(mod12,theta,ev5,omega=omega,sigma=sigma,nSub=1000)

# Concentration
pk_gcv <- sim5 %>%
  group_by(time) %>%
  # 10-90% Prediction interval
  summarise(medconc = median(cp),
            lowconc = quantile(cp,0.1),
            highconc = quantile(cp, 0.9)) %>%
  ungroup() %>%
  # time after last dose
  mutate(tald = time-156)

dat30 <- pk_gcv %>%
  mutate(study="Perrottet2009_lung_liver",
         drug="GCV",
         pop="adult")

# plot adults VGCV(oral) conc vs time -------------------------------
---------------
dose_vgcv <- 900 # 900 mg/12h for adults

# Define event record
ev6 <- et(amount.units = "mg", time.units = "hours") %>%
  add.dosing(dosing.to = "depot",
             dose = dose_vgcv,  # mg
             nbr.doses = 14,
             dosing.interval = 12,
             start.time = 0) %>%
  # 0-12h after the last dose
  add.sampling(seq(from=156,to=168.86,by=1))

# Perform simulation
# total number of subject: 1000
sim6 <- rxSolve(mod12,theta,ev6,omega=omega,sigma=sigma,nSub=1000)


# Concentration
pk_vgcv <- sim6 %>%
  group_by(time) %>%
```

```
  # 10-90% Prediction interval
  summarise(medconc = median(cp),
            lowconc = quantile(cp,0.1),
            highconc = quantile(cp, 0.9)) %>%
  ungroup() %>%
  # time after last dose
  mutate(tald = time-156)

dat31 <- pk_vgcv %>%
  mutate(study="Perrottet2009_lung_liver",
         drug="VGCV",
         pop="adult")
```

Zhao et al.(2009)

```
# 13.##zhao et al.(2009)## -----------------------------------------
-----------
# Define model -------------------------------------------------------
------
# CL = 8.04 x (CLcr/89)^2.93 + 3.62 x (BW/28) (L/h)
# V2 = 5.2 (L)
# Q  = 3.97 (L/h)
# V3 = 30.7 (L)
# Ka = 0.369 (h-1)
# Tlag = 0.743 (h)

# BSV (CV%): CL = 23.83%, V2 = 58.22%, Ka = 32.25%
# RUV: exponential.err = 20.93%
# CLcr: creatinine clearance (mL/min), calculated by Schwartz formula

# Typical patient: children(boys),10 years old,BW=30kg,SCR=70umol/L
# VGCV oral, 10 mg/kg/12h

set.seed(1234)
rxSetSeed(1234)

mod13 <- rxode2({
  CL   = TVCL*exp(eta.CL);
  V2   = TVV2*exp(eta.V2);
  Q    = TVQ;
  V3   = TVV3;
  KA   = TVKA*exp(eta.KA);
```

```
  C2 = centr/V2;
  C3 = peri/V3;
  d/dt(depot) = -KA*depot;
  d/dt(centr) = KA*depot - CL*C2 - Q*C2 + Q*C3;
  d/dt(peri)  = Q*C2 - Q*C3;
  alag(depot) = TLAG;

  cp = C2*exp(expo.err.sd);
})

# Children -----------------------------------------------------------
------
# Define typical patient: children
BW <- 30   # kg
HT <- 130  # cm
BSA <- sqrt(BW*HT/3600) # m2
SCR <- 70/88.4 # mg/dL (1mg/dL = 88.4umol/L)
k <- 0.55 # k = 0.55 for boys aged 2 years to less than 13 years
CLcr <- k*HT/SCR # ~ 90 mL/min/1.73 m2, calculated by Schwartz
formula
CLcr <- CLcr*(BSA/1.73) # ~ 54 mL/min

# Define fixed effect parameters
theta <- c(TVCL=8.04*((CLcr/89)^2.93) + 3.62*(BW/28),
         TVV2=5.2,
         TVQ =3.97,
         TVV3=30.7,
         TVKA=0.369,
         TLAG=0.743)

# Define between subject variability
omega <- lotri(eta.CL ~ 0.2383^2,
             eta.V2 ~ 0.5822^2,
             eta.KA ~ 0.3225^2)

# Define unexplained variability
sigma <- lotri(expo.err.sd ~ 0.2093^2)

# DOSE of VGCV(oral) input ------------------------------------------
---
dose_vgcv <- 10*BW # 10 mg/kg/12h for children

# Define event record
ev1 <- et(amount.units = "mg", time.units = "hours") %>%
```

```
  add.dosing(dosing.to = "depot",
             dose = dose_vgcv,  # mg
             nbr.doses = 14,
             dosing.interval = 12,
             start.time = 0) %>%
  # 0-12h after the last dose
  add.sampling(seq(from=156,to=168.93,by=1))

# Perform simulation
# total number of subject: 1000
sim1 <- rxSolve(mod13,theta,ev1,omega=omega,sigma=sigma,nSub=1000)

# Concentration
pk_vgcv <- sim1 %>%
  group_by(time) %>%
  # 10-90% Prediction interval
  summarise(medconc = median(cp),
            lowconc = quantile(cp,0.1),
            highconc = quantile(cp, 0.9)) %>%
  ungroup() %>%
  # time after last dose
  mutate(tald = time-156)

dat32 <- pk_vgcv %>%
  mutate(study="Zhao2009",
         drug="VGCV",
         pop="child")
```

Acosta et al.(2007)

```
# 14.##Acosta et al.(2007)## ----------------------------------------
-------------
# Define model ------------------------------------------------------
------
# Ka = 0.591 (h-1)
# CL = 0.146 x BW^1.68  (L/h)
# V  = 1.15 x BW (L)
# F1  = 0.536
# BSV (CV%): CL = 28.4%, F1 = 12.4%
# expo.err = 45.4%
# BW (kg)
```

```
# Typical patient:
neonates(boys),PMA=40weeks,HT=50cm,BW=3kg,SCR=30umol/L
# Dose: GCV iv, 5 mg/kg/12h, inf=1h; VGCV oral, 10 mg/kg/12h

set.seed(123456)
rxSetSeed(123456)

mod14 <- rxode2({
  CL   = TVCL*exp(eta.CL);
  V    = TVV;
  KA   = TVKA;
  F1   = TVF1*exp(eta.F1);

  C = centr/V;
  d/dt(depot) = -KA*depot;
  d/dt(centr) =  F1*KA*depot - CL*C;

  cp = C*exp(expo.err.sd);
})


# Neonates ------------------------------------------------------------
------
# Define typical patient: neonates
BW <- 3   # kg

# Define fixed effect parameters
theta <- c(TVCL=0.146*(BW^1.68),
         TVV=1.15*BW,
         TVKA=0.591,
         TVF1=0.563)

# Define between subject variability
omega <- lotri(eta.CL ~ 0.284^2, eta.F1 ~ 0.124^2)

# Define unexplained variability
sigma <- lotri(expo.err.sd ~ 0.454^2)


# DOSE of GCV(iv) input ----------------------------------------------
---
dose_gcv <- 5*BW # 5 mg/kg/12h for neonates

# Define event record
```

```
ev1 <- et(amount.units = "mg", time.units = "hours") %>%
  add.dosing(dosing.to = "centr",
             dose = dose_gcv,  # mg
             rate = dose_gcv/1, # infusion for 1h
             nbr.doses = 14,
             dosing.interval = 12,
             start.time = 0) %>%
  # 0-12h after the last dose
  add.sampling(seq(from=156,to=168,by=1))

# Perform simulation
# total number of subject: 1000
sim1 <- rxSolve(mod14,theta,ev1,omega=omega,sigma=sigma,nSub=1000)

# Concentration
pk_gcv <- sim1 %>%
  group_by(time) %>%
  # 10-90% Prediction interval
  summarise(medconc = median(cp),
            lowconc = quantile(cp,0.1),
            highconc = quantile(cp, 0.9)) %>%
  ungroup() %>%
  # time after last dose
  mutate(tald = time-156)


dat33 <- pk_gcv %>%
  mutate(study="Acosta2007",
         drug="GCV",
         pop="neonate")

# DOSE of VGCV(oral) input ------------------------------------------
---
dose_vgcv <- 10*BW # 10 mg/kg/12h for neonates

# Define event record
ev2 <- et(amount.units = "mg", time.units = "hours") %>%
  add.dosing(dosing.to = "depot",
             dose = dose_vgcv,  # mg
             nbr.doses = 14,
             dosing.interval = 12,
             start.time = 0) %>%
  # 0-12h after the last dose
  add.sampling(seq(from=156,to=168,by=1))
```

```
# Perform simulation
# total number of subject: 1000
sim2 <- rxSolve(mod14,theta,ev2,omega=omega,sigma=sigma,nSub=1000)

# Concentration
pk_vgcv <- sim2 %>%
  group_by(time) %>%
  # 10-90% Prediction interval
  summarise(medconc = median(cp),
            lowconc = quantile(cp,0.1),
            highconc = quantile(cp, 0.9)) %>%
  ungroup() %>%
  # time after last dose
  mutate(tald = time-156)


dat34 <- pk_vgcv %>%
  mutate(study="Acosta2007",
         drug="VGCV",
         pop="neonate")
```

Zhou et al.(1996)

```
# 15.##Zhou et al.(1996)## ----------------------------------------
# Define model ------------------------------------------------------
------
# CL = 0.262 + (0.00271 x ASCC) (L/h)
# V  = 0.627 + (0.437 x BW) (L)

# see in Table 2
# omega^2: CL = 0.125, V = 0.0904
# COV: covariance between CL and V 0.0813
# prop.err^2 = 0.00715

# Typical patient:
neonates(boys),PMA=40weeks,HT=50cm,BW=3kg,SCR=30umol/L
# ASCC, approximated creatininie clearance from serum (mL/min/1.73
m2), Schwartz formula
# Dose: GCV iv, 5 mg/kg/12h, infusion=1h

set.seed(12345)
rxSetSeed(12345)
```

```
mod15 <- rxode2({
  CL  = TVCL*exp(eta.CL);
  V   = TVV*exp(eta.V);

  C = centr/V;
  d/dt(centr) = - CL*C;

  cp = C*(1 + prop.err.sd);
})

# Neonates -------------------------------------------------------------
------
# Define typical patient: neonates
BW <- 3   # kg
HT <- 50  # cm
SCR <- 30/88.4 # mg/dL (1mg/dL = 88.4umol/L)
k <- 0.55 # k = 0.55 in the discussion
ASCC <- k*HT/SCR # ~ 81 mL/min/1.73 m2, calculated by Schwartz
formula

# Define fixed effect parameters
theta <- c(TVCL=0.262 + (0.00271*ASCC),
         TVV=0.627 + (0.437*BW))

# Define between subject variability
omega <- lotri(eta.CL + eta.V ~
              c(0.125,
                0.0813,0.0904))

# Define unexplained variability
sigma <- lotri(prop.err.sd ~ 0.00715)


# DOSE of GCV(iv) input ----------------------------------------------
---
dose_gcv <- 5*BW # 5 mg/kg/12h for neonates

# Define event record
ev1 <- et(amount.units = "mg", time.units = "hours") %>%
  add.dosing(dosing.to = "centr",
           dose = dose_gcv,  # mg
           rate = dose_gcv/1, # infusion for 1h
           nbr.doses = 14,
```

```
           dosing.interval = 12,
           start.time = 0) %>%
  # 0-12h after the last dose
  add.sampling(seq(from=156,to=168,by=1))

# Perform simulation
# total number of subject: 1000
sim1 <- rxSolve(mod15,theta,ev1,omega=omega,sigma=sigma,nSub=1000)

# Concentration
pk_gcv <- sim1 %>%
  group_by(time) %>%
  # 10-90% Prediction interval
  summarise(medconc = median(cp),
          lowconc = quantile(cp,0.1),
          highconc = quantile(cp, 0.9)) %>%
  ungroup() %>%
  # time after last dose
  mutate(tald = time-156)

dat35 <- pk_gcv %>%
  mutate(study="Zhou1996",
        drug="GCV",
        pop="neonate")
```

Yuen et al.(1995)

```
# 16.##Yuen et al.(1995)## -----------------------------------------
-----------
# Define model -----------------------------------------------------
------
# CL = 0.382 + 0.168 x BW x CLcr/100 x (1-Trans) x (1-CMV) (L/h)
# V1 = 0.381 x BW (L)
# Q  = 13.4 (L/h)
# V2 = 0.511 x BW (L)

# BSV (CV%): CL = 47.5%, V1 = 27.5%
# prop.err = 36.1%

# CLcr: creatinine clearance (mL/min), calculated by C-G formula
# T: 0 for nontransplant patients and 0.76 for transplant patients
# CMV: 0 for CMV-shedding patients and 0.41 for patients with CMV
retinitis
```

```
# Typical patient: adult(male),40 years old,BW=70kg,SCR=95umol/L
# Dose: GCV iv, 5 mg/kg/12h

set.seed(12345)
rxSetSeed(12345)

mod16 <- rxode2({
  CL  = TVCL*exp(eta.CL);
  V1  = TVV1*exp(eta.V1);
  Q   = TVQ;
  V2  = TVV2;

  C1 = centr/V1;
  C2 = peri/V2
  d/dt(centr) = - CL*C1 - Q*C1 + Q*C2;
  d/dt(peri)  = Q*C1 - Q*C2;

  cp = C1*(1 + prop.err.sd);
})


# Adults ------------------------------------------------------------
------
# Define typical patient: adults
BW <- 70   # kg
AGE <- 40  # years old
SCR <- 95/88.4 # mg/dL (1mg/dL = 88.4umol/L)
CLcr <- (140-AGE)*BW/(72*SCR) # ~ 90 mL/min
Trans <- 0 # 0 for nontransplant patients and 0.76 for transplant
patients
CMV <- 0 # 0 for CMV-shedding patients and 0.41 for patients with CMV
retinitis

# Define fixed effect parameters
theta <- c(TVCL=0.382 + 0.168*BW *CLcr/100*(1-Trans)*(1-CMV),
         TVV1=0.381*BW,
         TVQ=13.4,
         TVV2=0.511*BW)

# Define between subject variability
omega <- lotri(eta.CL ~ 0.475^2, eta.V1 ~ 0.275^2)

# Define unexplained variability
```

```
sigma <- lotri(prop.err.sd ~ 0.361^2)


# DOSE of GCV(iv) input ---------------------------------------------
---
dose_gcv <- 5*BW # 5 mg/kg/12h for adults

# Define event record
ev1 <- et(amount.units = "mg", time.units = "hours") %>%
  add.dosing(dosing.to = "centr",
             dose = dose_gcv,  # mg
             rate = dose_gcv/1, # infusion for 1h
             nbr.doses = 14,
             dosing.interval = 12,
             start.time = 0) %>%
  # 0-12h after the last dose
  add.sampling(seq(from=156,to=168,by=1))

# Perform simulation
# total number of subject: 1000
sim1 <- rxSolve(mod16,theta,ev1,omega=omega,sigma=sigma,nSub=1000)

# Concentration
pk_gcv <- sim1 %>%
  group_by(time) %>%
  # 10-90% Prediction interval
  summarise(medconc = median(cp),
            lowconc = quantile(cp,0.1),
            highconc = quantile(cp, 0.9)) %>%
  ungroup() %>%
  # time after last dose
  mutate(tald = time-156)


dat36 <- pk_gcv %>%
  mutate(study="Yuen1995",
        drug="GCV",
        pop="adult")

###Merge data###------------------------------------------
# combine data
dat <-
rbind(dat1,dat2,dat3,dat5,dat6,dat7,dat8,dat9,dat10,dat11,dat12,
```

```
dat13,dat14,dat15,dat16,dat17,dat18,dat19,dat20,dat21,dat22,

dat24,dat25,dat26,dat27,dat28,dat29,dat30,dat31,dat32,dat33,
           dat34,dat35,dat36) %>%
  select(tald,lowconc,medconc,highconc,study,drug,pop) %>%
  rbind(dat4,dat23)

# filter different drug's data
dat_gcv <- dat %>%
  filter(drug=="GCV")

dat_vgcv <- dat %>%
  filter(drug=="VGCV")

# plot -----------------------------------------------------------
# GCV-Children -----------------------------------------------------
------
dat_gcv1 <- dat_gcv %>%
  filter(pop=="child")

# arrange by levels
dat_gcv1$study <- factor(dat_gcv1$study, levels =
c("Li2021","Franck2021","Nguyen2021"))

# change the label names
labelname1 <- c("Li2021"="Li et al. (2021)",
             "Franck2021"="Franck et al. (2021)",
             "Nguyen2021"="Nguyen et al. (2021)")

y_breaks <- c(0,10,20,30)

pl1 <- dat_gcv1 %>%
  ggplot(mapping=aes(x=tald, y=medconc)) +
  geom_line(size=1.5, color="orange") +
  geom_ribbon(aes(x=tald,ymin=lowconc,ymax=highconc), fill =
"orange",alpha=0.2) +
  facet_wrap( ~ study, nrow = 1,
          labeller = labeller(study=as_labeller(labelname1))) +
  theme_bw(base_size = 30) +
  scale_x_continuous("Time (h)", limits = c(0,12.1), breaks =
c(0,2,4,6,8,10,12)) +
  scale_y_continuous("Concentration (mg/L)") +
  coord_cartesian(ylim = c(0,35)) +
```

```
  ggtitle("C Children: 30 kg, 10 years old") +
  theme (legend.position = "none",
         plot.background = element_blank(),
         panel.grid.minor = element_blank(),
         panel.grid.major = element_blank(),
         plot.title = element_text(hjust = 0,
                                   size = 30))


jpeg(filename = paste0(output_dir,"/GCV_children.jpg"),
     width=8000, height=2500, res=300)
print(pl1)
dev.off()


# GCV-adults --------------------------------------------------------
------
dat_gcv2 <- dat_gcv %>%
  filter(pop=="adult")

# arrange by levels
dat_gcv2$study <- factor(dat_gcv2$study, levels = c("Yuen1995",
                                                    "Perrottet2009_kidney",
                                                    "Perrottet2009_heart",

"Perrottet2009_lung_liver",
                                                    "Caldés2009",
                                                    "Horvatits2014",
                                                    "Krens2020",
                                                    "Lalagkas2023"))

# change the label names
labelname2 <- c("Lalagkas2023"="Lalagkas et al. (2023)",
                "Krens2020"="Krens et al. (2020)",
                "Horvatits2014"="Horvatits et al. (2014)",
                "Caldés2009"="Caldés et al. (2009)",
                "Perrottet2009_kidney"="Perrottet et al.
(2009)\nkidney",
                "Perrottet2009_heart"="Perrottet et al. (2009)\nheart",
                "Perrottet2009_lung_liver"="Perrottet et al.
(2009)\nlung/liver",
                "Yuen1995"="Yuen et al. (1995)")

pl2 <- dat_gcv2 %>%
```

```r
  ggplot(mapping=aes(x=tald, y=medconc)) +
  geom_line(size=1.5, color="orange") +
  geom_ribbon(aes(x=tald,ymin=lowconc,ymax=highconc), fill =
"orange",alpha=0.2) +
  facet_wrap( ~ study, nrow = 1,
             labeller = labeller(study=as_labeller(labelname2))) +
  theme_bw(base_size = 30) +
  scale_x_continuous("Time (h)", limits = c(0,12.1), breaks =
c(0,2,4,6,8,10,12)) +
  scale_y_continuous("Concentration (mg/L)") +
  coord_cartesian(ylim = c(0,35)) +
  ggtitle("D Adults: 70 kg, 40 years old") +
  theme (legend.position = "none",
        plot.background = element_blank(),
        panel.grid.minor = element_blank(),
        panel.grid.major = element_blank(),
        plot.title = element_text(hjust = 0,
                                  size = 30))


jpeg(filename = paste0(output_dir,"/GCV_adults.jpg"),
     width=8000, height=2500, res=300)
print(pl2)
dev.off()



# GCV-neonates -------------------------------------------------------
------
dat_gcv3 <- dat_gcv %>%
  filter(pop=="neonate")

# arrange by levels
dat_gcv3$study <- factor(dat_gcv3$study, levels = c("Zhou1996",
                                                    "Acosta2007",
                                                    "Nguyen2021"))

# change the label names
labelname3 <- c("Zhou1996"="Zhou et al. (1996)",
                "Acosta2007"="Acosta et al. (2007)",
                "Nguyen2021"="Nguyen et al. (2021)")

pl3 <- dat_gcv3 %>%
  ggplot(mapping=aes(x=tald, y=medconc)) +
```

```r
  geom_line(size=1.5, color="orange") +
  geom_ribbon(aes(x=tald,ymin=lowconc,ymax=highconc), fill =
"orange",alpha=0.2) +
  facet_wrap( ~ study, nrow = 1,
             labeller = labeller(study=as_labeller(labelname3))) +
  theme_bw(base_size = 30) +
  scale_x_continuous("Time (h)", limits = c(0,12.1), breaks =
c(0,2,4,6,8,10,12)) +
  scale_y_continuous("Concentration (mg/L)") +
  coord_cartesian(ylim = c(0,35)) +
  ggtitle("A Neonates: 3 kg, PMA 40 weeks") +
  theme (legend.position = "none",
        plot.background = element_blank(),
        panel.grid.minor = element_blank(),
        panel.grid.major = element_blank(),
        plot.title = element_text(hjust = 0,
                                  size = 30))


jpeg(filename = paste0(output_dir,"/GCV_neonates.jpg"),
    width=8000, height=2500, res=300)
print(pl3)
dev.off()

# GCV-infants -------------------------------------------------------
-----
dat_gcv4 <- dat_gcv %>%
  filter(pop=="infant")

# arrange by levels
dat_gcv4$study <- factor(dat_gcv4$study, levels = c("Li2021",
                                                    "Franck2021",
                                                    "Nguyen2021"))

# change the label names
labelname4 <- c("Li2021"="Li et al. (2021)",
               "Franck2021"="Franck et al. (2021)",
               "Nguyen2021"="Nguyen et al. (2021)")

pl4 <- dat_gcv4 %>%
  ggplot(mapping=aes(x=tald, y=medconc)) +
  geom_line(size=1.5, color="orange") +
  geom_ribbon(aes(x=tald,ymin=lowconc,ymax=highconc), fill =
"orange",alpha=0.2) +
```

```
  facet_wrap( ~ study, nrow = 1,
              labeller = labeller(study=as_labeller(labelname4))) +
  theme_bw(base_size = 30) +
  scale_x_continuous("Time (h)", limits = c(0,12.1), breaks =
c(0,2,4,6,8,10,12)) +
  scale_y_continuous("Concentration (mg/L)") +
  coord_cartesian(ylim = c(0,35)) +
  ggtitle("B Infants: 10 kg, 1 year old") +
  theme (legend.position = "none",
        plot.background = element_blank(),
        panel.grid.minor = element_blank(),
        panel.grid.major = element_blank(),
        plot.title = element_text(hjust = 0,
                                  size = 30))


jpeg(filename = paste0(output_dir,"/GCV_infants.jpg"),
    width=8000, height=2500, res=300)
print(pl4)
dev.off()

# add a blank grid by plot_grid for GCV_neonates
f1 <- plot_grid(pl3 + theme(axis.title = element_blank()), # remove
axis titles
             NULL,
             align = "h",
             rel_widths = c(3.2,5)) # adjust the relative widths

# add a blank grid by plot_grid for GCV_infants
f2 <- plot_grid(pl4 + theme(axis.title = element_blank()), # remove
axis titles
             NULL,
             align = "h",
             rel_widths = c(3.2,5)) # adjust the relative widths

# add a blank grid by plot_grid for GCV_children
f3 <- plot_grid(pl1 + theme(axis.title = element_blank()), # remove
axis titles
             NULL,
             align = "h",
             rel_widths = c(3.2,5)) # adjust the relative widths

# combine GCV
pl <- plot_grid(f1,
```

```
             f2,
             f3,
             pl2 + theme(axis.title = element_blank()), # remove
axis titles
             nrow = 4,
             axis = "l",
             rel_widths = c(1,0.8,1))

pl_neo_inf <- plot_grid(pl3 + theme(axis.title = element_blank()),
                    NULL,
                    pl4 + theme(axis.title = element_blank()),
                    nrow = 1,
                    rel_widths = c(1.1,0.6,1.1))

pl_neo_inf

pl <- plot_grid(pl_neo_inf,
             f3,
             pl2 + theme(axis.title = element_blank()),
             nrow = 3,axis = "l",
             rel_widths = c(0.05,1,1))

# Add a common axis title
library(ggpubr) # annotate_figure()

p <- annotate_figure(pl, # the objective
                    left=text_grob("Concentration (mg/L)", # on the
left
                                 face = "bold",
                                 size = 30,
                                 rot = 90), # the angle to rotate the
text
                    bottom=text_grob("Time (h)",
                                  face = "bold",
                                  size = 30))


# output ------------------------------------------------------------------
jpeg(filename = paste0(output_dir,"/GCV1.jpg"),
    width=9000, height=6200, res=300)
print(p)
dev.off()
```

```
# VGCV-Children -------------------------------------------------------
------
dat_vgcv1 <- dat_vgcv %>%
  filter(pop=="child")

# arrange by levels
dat_vgcv1$study <- factor(dat_vgcv1$study, levels=c("Zhao2009",
                                                     "Vezina2010",
                                                     "Vezina2014",
                                                     "Facchin2019",
                                                     "Franck2021",
                                                     "Nguyen2021"))

# change the label names
labelname1 <- c("Zhao2009"="Zhao et al. (2009)",
                "Vezina2010"="Vezina et al. (2010)",
                "Vezina2014"="Vezina et al. (2014)",
                "Facchin2019"="Facchin et al. (2019)",
                "Franck2021"="Franck et al. (2021)",
                "Nguyen2021"="Nguyen et al. (2021)")

y_breaks <- c(0,10,20,30)

pl1 <- dat_vgcv1 %>%
  ggplot(mapping=aes(x=tald, y=medconc)) +
  geom_line(size=1.5, color="orange") +
  geom_ribbon(aes(x=tald,ymin=lowconc,ymax=highconc), fill =
"orange",alpha=0.2) +
  facet_wrap( ~ study, nrow = 1,
          labeller = labeller(study=as_labeller(labelname1))) +
  theme_bw(base_size = 30) +
  scale_x_continuous("Time (h)", limits = c(0,12.1), breaks =
c(0,2,4,6,8,10,12)) +
  scale_y_continuous("Concentration (mg/L)") +
  coord_cartesian(ylim = c(0,20)) +
  ggtitle("C Children: 30 kg, 10 years old") +
  theme (legend.position = "none",
        plot.background = element_blank(),
        panel.grid.minor = element_blank(),
        panel.grid.major = element_blank(),
        plot.title = element_text(hjust = 0,
                                  size = 30))
```

```
jpeg(filename = paste0(output_dir,"/VGCV_children.jpg"),
     width=8000, height=2500, res=300)
print(pl1)
dev.off()


# VGCV-adults --------------------------------------------------------
-------
dat_vgcv2 <- dat_vgcv %>%
  filter(pop=="adult")

# arrange by levels
dat_vgcv2$study <- factor(dat_vgcv2$study,
levels=c("Perrottet2009_kidney",
                                            "Perrottet2009_heart",

"Perrottet2009_lung_liver",
                                            "Caldés2009",
                                            "Vezina2014",
                                            "Facchin2019",
                                            "Chen2021",
                                            "Lalagkas2023"))

# change the label names
labelname2 <- c("Perrottet2009_kidney"="Perrottet et al.
(2009)\nkidney",
             "Perrottet2009_heart"="Perrottet et al. (2009)\nheart",
             "Perrottet2009_lung_liver"="Perrottet et al.
(2009)\nlung/liver",
             "Caldés2009"="Caldés et al. (2009)",
             "Vezina2014"="Vezina et al. (2014)",
             "Facchin2019"="Facchin et al. (2019)",
             "Chen2021"="Chen et al. (2021)",
             "Lalagkas2023"="Lalagkas et al. (2023)")

pl2 <- dat_vgcv2 %>%
  ggplot(mapping=aes(x=tald, y=medconc)) +
  geom_line(size=1.5, color="orange") +
  geom_ribbon(aes(x=tald,ymin=lowconc,ymax=highconc), fill =
"orange",alpha=0.2) +
  facet_wrap( ~ study, nrow = 1,
          labeller = labeller(study=as_labeller(labelname2))) +
  theme_bw(base_size = 30) +
```

```
  scale_x_continuous("Time (h)", limits = c(0,12.1), breaks =
c(0,2,4,6,8,10,12)) +
  scale_y_continuous("Concentration (mg/L)") +
  coord_cartesian(ylim = c(0,20)) +
  ggtitle("D Adults: 70 kg, 40 years old") +
  theme (legend.position = "none",
        plot.background = element_blank(),
        panel.grid.minor = element_blank(),
        panel.grid.major = element_blank(),
        plot.title = element_text(hjust = 0,
                                  size = 30))


jpeg(filename = paste0(output_dir,"/VGCV_adults.jpg"),
    width=8000, height=2500, res=300)
print(pl2)
dev.off()

# VGCV-neonates -----------------------------------------------------
---------
dat_vgcv3 <- dat_vgcv %>%
  filter(pop=="neonate")

# arrange by levels
dat_vgcv3$study <- factor(dat_vgcv3$study, levels=c("Acosta2007",
                                                    "Nguyen2021"))

# change the label names
labelname3 <- c("Acosta2007"="Acosta et al. (2007)",
                "Nguyen2021"="Nguyen et al. (2021)")

pl3 <- dat_vgcv3 %>%
  ggplot(mapping=aes(x=tald, y=medconc)) +
  geom_line(size=1.5, color="orange") +
  geom_ribbon(aes(x=tald,ymin=lowconc,ymax=highconc), fill =
"orange",alpha=0.2) +
  facet_wrap( ~ study, nrow = 1,
           labeller = labeller(study=as_labeller(labelname3))) +
  theme_bw(base_size = 30) +
  scale_x_continuous("Time (h)", limits = c(0,12.1), breaks =
c(0,2,4,6,8,10,12)) +
  scale_y_continuous("Concentration (mg/L)") +
  coord_cartesian(ylim = c(0,20)) +
  ggtitle("A Neonates: 3 kg, PMA 40 weeks") +
```

```
  theme (legend.position = "none",
        plot.background = element_blank(),
        panel.grid.minor = element_blank(),
        panel.grid.major = element_blank(),
        plot.title = element_text(hjust = 0,
                                  size = 30))


jpeg(filename = paste0(output_dir,"/VGCV_neonates.jpg"),
    width=8000, height=2500, res=300)
print(pl3)
dev.off()


# VGCV-infants -------------------------------------------------------
--------
dat_vgcv4 <- dat_vgcv %>%
  filter(pop=="infant")

# arrange by levels
dat_vgcv4$study <- factor(dat_vgcv4$study, levels=c("Vezina2014",
                                                     "Franck2021",
                                                     "Nguyen2021"))

# change the label names
labelname4 <- c("Vezina2014"="Vezina et al. (2014)",
                "Franck2021"="Franck et al. (2021)",
                "Nguyen2021"="Nguyen et al. (2021)")

pl4 <- dat_vgcv4 %>%
  ggplot(mapping=aes(x=tald, y=medconc)) +
  geom_line(size=1.5, color="orange") +
  geom_ribbon(aes(x=tald,ymin=lowconc,ymax=highconc), fill =
"orange",alpha=0.2) +
  facet_wrap( ~ study, nrow = 1,
            labeller = labeller(study=as_labeller(labelname4))) +
  theme_bw(base_size = 30) +
  scale_x_continuous("Time (h)", limits = c(0,12.1), breaks =
c(0,2,4,6,8,10,12)) +
  scale_y_continuous("Concentration (mg/L)") +
  coord_cartesian(ylim = c(0,20)) +
  ggtitle("B Infants: 10 kg, 1 year old") +
  theme (legend.position = "none",
        plot.background = element_blank(),
```

```
        panel.grid.minor = element_blank(),
        panel.grid.major = element_blank(),
        plot.title = element_text(hjust = 0,
                                  size = 30))


jpeg(filename = paste0(output_dir,"/VGCV_infants.jpg"),
    width=8000, height=2500, res=300)
print(pl4)
dev.off()

# add a blank grid by plot_grid for VGCV_neonates
f1 <- plot_grid(pl3 + theme(axis.title = element_blank()), # remove
axis titles
             NULL,
             align = "l",
             rel_widths = c(2.2,5)) # adjust the relative widths

# add a blank grid by plot_grid for VGCV_infants
f2 <- plot_grid(pl4 + theme(axis.title = element_blank()), # remove
axis titles
             NULL,
             align = "l",
             rel_widths = c(3.22,4)) # adjust the relative widths

# add a blank grid by plot_grid for VGCV_children
f3 <- plot_grid(pl1 + theme(axis.title = element_blank()), # remove
axis titles
             NULL,
             align = "l",
             rel_widths = c(6.1,2)) # adjust the relative widths

# combine GCV-Children with GCV-adults
pl <- plot_grid(f1,
             f2,
             f3,
             pl2 + theme(axis.title = element_blank()), # remove
axis titles,
             nrow = 4,
             axis = "l",
             rel_widths = c(1,1,1))

pl_neo_inf <- plot_grid(pl3 + theme(axis.title = element_blank()),
                    NULL,
```

```
                         pl4 + theme(axis.title = element_blank()),
                         NULL,
                         nrow = 1,
                         rel_widths = c(2.1,0.8,3,2))

pl <- plot_grid(pl_neo_inf,
                f3,
                pl2 + theme(axis.title = element_blank()),
                nrow = 3,
                rel_widths = c(1,1,1))

# Add a common axis title
library(ggpubr) # annotate_figure()

p <- annotate_figure(pl, # the objective
                     left=text_grob("Concentration (mg/L)", # on the
left
                                    face = "bold",
                                    size = 30,
                                    rot = 90), # the angle to rotate the
text
                     bottom=text_grob("Time (h)",
                                      face = "bold",
                                      size = 30))


# output -----------------------------------------------------------
jpeg(filename = paste0(output_dir,"/VGCV1.jpg"),
     width=9000, height=6200, res=300)
print(p)
dev.off()
```

R Codes of Covariate Effects Evaluation

```
rm(list=ls())
#set working directory to current folder
curr.dir<-dirname(rstudioapi::getActiveDocumentContext()$path)
setwd(curr.dir)

# library(devtools)
# devtools::install_github("nicolash2/ggbrace")

# load R packages
library(tidyverse) # for data visualisation and manipulation
library(readxl)
library(ggbrace) # for adding braces

# Create fold for figure output
output_dir <- "forest_figure"
if (!file.exists(output_dir)) {dir.create (output_dir)}

# Read in data
dat <- read_xlsx("forest_GCV230604.xlsx",sheet = "Sheet2",range =
"A1:I35",
                col_names = TRUE) %>%
  mutate(mean=(lower+upper)/2)

# reverse y labels
y_label <- rev(dat$study)

# use ggplot to draw forest figure
pl <- ggplot(data = dat, aes(x = mean, y = number)) +
  geom_point(size=0) +
  geom_vline(xintercept = 1) +
  geom_errorbarh(aes(xmin=lower, xmax=upper), height = 0.4, size = 1,
color = "darkorange") +
  scale_x_log10("Covariate effect on CL", breaks =
c(0.1,0.25,0.5,0.8,1,1.25,2,3)) +
  scale_y_continuous(NULL, expand = c(0,0),breaks = c(1:34), labels =
y_label) +
  annotate("rect", xmin = 0.8, xmax = 1.25, ymin = 0.5, ymax = 34.5,
          fill = "orange", alpha = 0.4) +
  theme_bw() +
  theme(panel.grid = element_blank(),
       rect = element_blank(),
```

```
      axis.line.x = element_line(),
      axis.text.x = element_text(size = 13, face = "bold"),
      axis.ticks.y = element_blank(),
      # add space between y labels and the graph
      axis.text.y = element_text(margin = margin(0,3,0,0,"cm"),
                           # family = "serif", # Times New Roman
                           size = 13,
                           face = "bold"),
      axis.title.x = element_text(margin = margin(0.7,0,0,0,"cm"),
                            size = 16, face = "bold")) +
  geom_brace(aes(x=c(0.13,0.15), y=c(21,34), label = "Weight"),
           inherit.data = FALSE,
           rotate = 270,
           labelsize = 4.5,
           labeldistance = 0.03) +
  geom_brace(aes(x=c(0.14,0.15), y=c(8,12),
              label = "CLcr (20-130) [mL/min]"),
           inherit.data = FALSE,
           rotate = 270,
           labelsize = 4.5,
           labeldistance = 0.05) +
  geom_brace(aes(x=c(0.145,0.15), y=c(19,20)),
           inherit.data = FALSE,
           rotate = 270) +
  annotate("text", x=0.087, y=19.5, label = bquote("CrCL: Schwartz
(20-120) [mL/min/1.73"~m^2~"]"), size = 4.5) +
  geom_brace(aes(x=c(0.05,0.055), y=c(13,20),
              label = bquote("eGFR")),
           inherit.data = FALSE,
           rotate = 270,
           labelsize = 4.5,
           labeldistance = 0.05) +
  annotate("text", x=0.086, y=18, label = bquote("eGFR: Schwartz (20-
120) [mL/min/1.73"~m^2~"]"), size = 4.5) +
  annotate("text", x=0.086, y=17, label = bquote("ASCC: Schwartz (20-
120) [mL/min/1.73"~m^2~"]"), size = 4.5) +
  annotate("text", x=0.0895, y=16, label = bquote("eGFR: Gao (20-120)
[mL/min/1.73"~m^2~"]"), size = 4.5) +
  annotate("text", x=0.0912, y=14.5, label = bquote("CKD-EPI (20-120)
[mL/min/1.73"~m^2~"]"), size = 4.5) +
  geom_brace(aes(x=c(0.145,0.15), y=c(14,15)),
           inherit.data = FALSE,
           rotate = 270) +
```

```
  annotate("text", x=0.09, y=13, label = bquote(""~GFR[MDRD]~" (20-
120) [mL/min/1.73"~m^2~"]"), size = 4.5) +
  annotate("text", x=0.103, y=7, label = paste("SCR (22-265)
[","\u00B5","mol/L]"), size = 4.5) +
  annotate("text", x=0.105, y=6, label = bquote("BSA (0.54-1.95)
["~m^2~"]"), size = 4.5) +
  geom_brace(aes(x=c(0.145,0.15), y=c(4,5),label = "Sex (M/F)"),
           inherit.data = FALSE,
           rotate = 270,
           labelsize = 4.5,
           labeldistance = 0.06) +
  annotate("text", x=0.11, y=3, label = "Critically ill (Y/N)", size
= 4.5) +
  annotate("text", x=0.11, y=2, label = "Transplant (Y/N)", size =
4.5) +
  annotate("text", x=0.115, y=1, label = "CMV (Y/N)", size = 4.5) +
  geom_brace(aes(x=c(2.5,2.6), y=c(33,34),
              label = "Neonates\n(1-5)[kg]"),
           inherit.data = FALSE,
           rotate = 90,
           labelsize = 4,
           labeldistance = 0.01,
           bending = 0.23) +
  geom_brace(aes(x=c(2.5,2.6), y=c(29,32),
              label = "Infants\n(5-16)[kg]"),
           inherit.data = FALSE,
           rotate = 90,
           labelsize = 4,
           labeldistance = 0.01) +
  geom_brace(aes(x=c(2.5,2.6), y=c(24,28),
              label = "Children\n(16-40)[kg]"),
           inherit.data = FALSE,
           rotate = 90,
           labelsize = 4,
           labeldistance = 0.01) +
  geom_brace(aes(x=c(2.48,2.55), y=c(21,23),
              label = "Adults\n(40-100)[kg]"),
           inherit.data = FALSE,
           rotate = 90,
           labelsize = 4,
           labeldistance = 0.01)

# output the figure
jpeg(filename = paste0(output_dir,"/forest_GCV.jpg"),
```

```
    width=6000, height=3000, res=300)
print(pl)
dev.off()
```

R Codes of AUC Calculator based on MAP-BE

# Online AUC calculator based on MAP-BE
# Author: Wenyu Yang
# Email: 21211030109@m.fudan.edu.cn

# Globally used objects and functions

```
# -----------------------------------------------------------------------
-----------
# Load package libraries
library(shiny)  #Interactive applications
library(plyr)
library(tidyverse)  #Plotting
library(rxode2) #Differential equation solver
library(MASS)
library(rhandsontable) # dynamic table
#-------------------- Global parameter and setting --------------------
-# ####
# 1. Prior PK information from a developed PPK model: Franck et al.
(2021)
# Population typical value: CL, Vc, Q, Vp, KA, F, Tlag
POPpar <- c(6.9, 9.7, 10.9, 7.6, 0.73, 0.43, 0.33)

# Omega matrix (IIV): CL, Vc, KA, F
OMEGA <- matrix(c(0.44, 0, 0, 0,
               0, 0.59, 0, 0,
               0, 0, 0.7, 0,
               0, 0, 0, 0.31),4,4)
# Sigma matrix (RUV): prop.err=0, add.err=0.98 mg/L
SIGMA <-c(0, 0.98^2)

# initial etas
init_eta <- c(0,0,0,0)

# 2. Define model
```

```
# Function containing differential equations for amount in each
compartment
mod <- rxode2({
  CL  = indCL;
  Vc  = indVc;
  Q   = indQ;
  Vp  = indVp;
  KA  = indKA;
  F1  = indF1;
  Tlag = indTlag;

  conc  = centr/Vc;                              # central
compartment concentration
  conp  = peri/Vp;                               # peripheral
compartment concentration
  d/dt(depot) = -KA*depot;                        # depot
compartment
  d/dt(centr) = F1*KA*depot - CL*conc - Q*conc + Q*conp; # central
compartment amount
  d/dt(peri)  = Q*conc - Q*conp;                   # peripheral
compartment amount
  alag(depot) = Tlag;                            # lag time of
absorption

})

# 3. Define objective function for MAP estimator
# For more information on obtaining the MAP estimate,
# have a look at the following publication:
# https://www.ncbi.nlm.nih.gov/pmc/articles/PMC3339294/
obj.MAP <- function(eta, # ETA vector to optimize
                 omega, # OMEGA matrix for IIV
                 sigma, # sigma square for residual error
                 weight,
                 crcl, # creatinine clearance (mL/min/1.73 m2)
                 POPpar, # typical value for individual's parameters
                 mod, # model defined in RxODE
                 obs_data, # data frame for observations
                 dose_data # data frame for dosing
) {

  # Weight for each individual
  weight <- weight
  crcl <- crcl
```

```
  # typical value of individual's model parameters
  TVpar <- c(POPpar[1]*(weight/26.7)^0.75*(crcl/149.8)^0.88, # CL
(L/h)
             POPpar[2]*(weight/26.7), # Vc (L)
             POPpar[3],   # Q (L/h)
             POPpar[4]*(weight/26.7), # Vp (L)
             POPpar[5], # KA (1/h)
             POPpar[6], # F1
             POPpar[7]) # Tlag (h)

  # Define individual parameter values
  theta <- c(indCL = TVpar[1]*exp(eta[1]),     # CL (L/h)
             indVc = TVpar[2]*exp(eta[2]),     # Vc (L)
             indQ  = TVpar[3],     # Q (L/h)
             indVp = TVpar[4],     # Vp (L)
             indKA = TVpar[5]*exp(eta[3]),     # KA (1/h)
             indF1 = TVpar[6]*exp(eta[4]),     # F1
             indTlag = TVpar[7])     # Tlag (h)

  #### Define event record
  ev <- et(time = dose_data$time,
           amt  = dose_data$dose,
           evid = 1, # dosing records
           cmt = dose_data$cmt,
           dur = dose_data$dur) %>%
    add.sampling(obs_data$time)

  # Apply RxODE for simulation
  sim_data <- rxSolve(mod,theta,ev)

  # Individual prediction
  ipred <- sim_data$conc

  # sigma2 for combined error model
  sig2j <- ipred^2*sigma[1] + sigma[2]

  with(obs_data, sum(log(sig2j)+(ipred - conc)^2/sig2j,na.rm=TRUE) +
t(eta) %*% solve(omega) %*% eta)
}


# 4. MAP estimator
```

```
MAP.est <- function(init_eta, obs_data, dose_data, mod, weight, crcl,
POPpar, omega, sigma) {
  # optimization with simplex method
  res <- optim(par=init_eta, fn=obj.MAP, obs_data=obs_data,
dose_data=dose_data,
               mod=mod, weight=weight, crcl=crcl, POPpar=POPpar,
omega=omega, sigma=sigma,
               method="L-BFGS-B")

  # Weight for each individual
  weight <- weight
  crcl <- crcl

  # typical value of individual's model parameters
  TVpar <- c(POPpar[1]*(weight/26.7)^0.75*(crcl/149.8)^0.88, # CL
(L/h)
             POPpar[2]*(weight/26.7), # Vc (L)
             POPpar[3],   # Q (L/h)
             POPpar[4]*(weight/26.7), # Vp (L)
             POPpar[5], # KA (1/h)
             POPpar[6], # F1
             POPpar[7]) # Tlag (h)

  # eta estimated from map estimation
  map.eta <- res$par
  CL  <- TVpar[1]*exp(map.eta[1])
  Vc  <- TVpar[2]*exp(map.eta[2])
  Q   <- TVpar[3]
  Vp  <- TVpar[4]
  KA  <- TVpar[5]*exp(map.eta[3])
  F1  <- TVpar[6]*exp(map.eta[4])
  Tlag <- TVpar[7]

  # Collect the individual parameter values
  map_est <- c(indCL=CL, indVc=Vc, indQ=Q,indVp=Vp, indKA=KA,
indF1=F1, indTlag=Tlag)
  return(map_est)
}



# 5. Area under the curve (AUC) calculator
aucCal <- function(time,conc){
  temp <- vector()
```

```
  n <- length(time)
  for(i in 1:n-1){
    temp[i] <- (time[i+1] - time[i])*(conc[i+1] + conc[i])/2
  }
  auc <- sum(temp)
  return(auc)
}

# 6. AUC for individual
auc_ind <- function(data){
  res <- data %>%
    filter(time>=0,time <= 24) %>%
    summarise(AUC = aucCal(time,conc)) %>%
    as.numeric()
  return(res)
}


# 7. transform sample time into hour
obstimeAfterdose <- function(dose_data,sample_data){
  # remove the NA input
  dose_data <- dose_data[complete.cases(dose_data), ]
  sample_data <- sample_data[complete.cases(sample_data), ]
  # reorder the time: date, time
  dose_data <- dose_data %>%
    mutate(time = as.POSIXct(time,format="%H:%M"))%>%
    arrange(date, time)
  sample_data <- sample_data %>%
    mutate(time = as.POSIXct(time,format="%H:%M"))%>%
    arrange(date, time)

  # calculate time after the first dose
  sample_time <-as.numeric(difftime(sample_data$date,
dose_data[1,]$date, units = "hours")) +
    round(as.numeric(difftime(sample_data$time,dose_data[1,]$time,
units="hours")),2)

  obs_dat <- data.frame(time = sample_time, conc = sample_data$conc)
  return(obs_dat)
}

# 8. transform dosing time into hour
dostimeAfterdose <- function(dose_data,sample_data){
  # remove the NA input
```

```
  dose_data <- dose_data[complete.cases(dose_data), ]
  sample_data <- sample_data[complete.cases(sample_data), ]
  # reorder the time: date, time
  dose_data <- dose_data %>%
    mutate(time = as.POSIXct(time,format="%H:%M"))%>%
    arrange(date, time)
  sample_data <- sample_data %>%
    mutate(time = as.POSIXct(time,format="%H:%M"))%>%
    arrange(date, time)

  # calculate time after the first dose
  dose_time <- as.numeric(difftime(dose_data$date,
dose_data[1,]$date, units = "hours")) +
    round(as.numeric(difftime(dose_data$time, dose_data[1,]$time,
units="hours")),2)
  #transformed dataframes
  dos_dat <- data.frame(time = dose_time, dose = dose_data$dose,
                        cmt = case_when(dose_data$iv=="Y" ~ "centr",
                                        TRUE ~ "depot"),
                        dur =  case_when(dose_data$iv=="Y" ~
dose_data$duration,
                                         TRUE ~ 0))
  return(dos_dat)
}

# 9. for plotting: raw data + individual prediction
simPlot <- function(sim_ind_data,raw_dat) {

  # Compare the concentration-time profile from simulation with
observed data
  pl1 <- ggplot(data = sim_ind_data) +
    geom_line(aes(x = time, y = conc),size=1.5) +
    geom_point(inherit.aes = F, data = raw_dat, aes(x = time, y =
conc), color="red",size=3)+
    scale_y_continuous("Concentration (mg/L)") +
    scale_x_continuous("Time (hours)")
  return(pl1)
}

#-----------------------------Default-------------------------------
-------------
# Default table content
# rhandsontable cannot handle factor so stringsAsFactors = F
df1.Dose <- data.frame(date = c(Sys.Date(),Sys.Date()),
```

```r
                        time = c("08:00", "20:10"),
                        dose  = c(40, 400),
                        iv = c("Y", "N"),
                        duration = c(1, 0),
                        stringsAsFactors = F)
df1.Sample <- data.frame(date = c(Sys.Date()),
                          time = c("23:36"),
                          conc = c(7.6),
                          stringsAsFactors = F)
# Default table content in time
df.sample <- obstimeAfterdose(df1.Dose,df1.Sample)
df.dose <- dostimeAfterdose(df1.Dose,df1.Sample)
# Use MAP estimator for individual parameters
df_par_ind <- MAP.est(init_eta, df.sample, df.dose,mod,
                      weight = 8, crcl = 96, POPpar, OMEGA, SIGMA)

df_max_time <- max(c(df.dose$time,df.sample$time))

# Sample times: when a concentration was collected
df_sample_times <- seq(from=0, to=df_max_time+36, by=0.1)
# Define event record
df_ev <- et(time = df.dose$time,
         amt  = df.dose$dose,
         evid = 1, # dosing records
         dur = df.dose$dur,
         cmt = df.dose$cmt) %>%
  add.sampling(df_sample_times)

# Simulation for individual
df_ind_data <- rxSolve(mod, params = df_par_ind, events = df_ev)
```

Define Shiny Server

```
shinyServer(function(input, output, session) {

    # Dynamic table
    dynamicTable <- reactiveValues(dose_data = NULL)

    # add a row to the dose table
    observeEvent(input$addPoint, {
        df_temp1 <- hot_to_r(input$table1)
        # rhandsontable cannot handle factor so stringsAsFactors = F
        dynamicTable$dose_data <- data.frame(date=c(df_temp1$date,NA),
                                             time=c(df_temp1$time,NA),
                                             dose=c(df_temp1$dose,NA),
                                             iv=c(df_temp1$iv,NA),

duration=c(df_temp1$duration,NA),
                                             stringsAsFactors = F)
    })

    # drop a row from the dose table
    observeEvent(input$dropPoint, {
        df_temp1 <- hot_to_r(input$table1)
        dynamicTable$dose_data <- df_temp1[-nrow(df_temp1),]
    })

    # update the dose table
    output$table1 <- renderRHandsontable({
        # converts the R dataframe to rhansontable object
        if (is.null(dynamicTable$dose_data))
return(rhandsontable(df1.Dose))
        rhandsontable(dynamicTable$dose_data)
    })


     # Dynamic table
    dynamicTable <- reactiveValues(data = NULL)

    # add a row to the sample table
    observeEvent(input$addLink, {
        df_temp2 <- hot_to_r(input$table2)
        # rhandsontable cannot handle factor so stringsAsFactors = F
        dynamicTable$data <- data.frame(date=c(df_temp2$date,NA),
                                        time=c(df_temp2$time,NA),
```

```
                                    conc=c(df_temp2$conc,NA),
                                    stringsAsFactors = F)
    })

    # drop a row from the sample table
    observeEvent(input$dropLink, {
        df_temp2 <- hot_to_r(input$table2)
        dynamicTable$data <- df_temp2[-nrow(df_temp2),]
    })

    # update the sample sample table
    output$table2 <- renderRHandsontable({
        # converts the R dataframe to rhansontable object
        if (is.null(dynamicTable$data))
return(rhandsontable(df1.Sample))
        rhandsontable(dynamicTable$data)
    })

    ## bayesian forcasting result: updated by clicking action button
    res <- eventReactive(input$submit, {
        set.seed(123456)
        # get the original data from table input
        dat_dose <- hot_to_r(input$table1)
        dat_sample <- hot_to_r(input$table2)
        # transform into the dataset
        obs_dat <- obstimeAfterdose(dat_dose,dat_sample)
        dos_dat <- dostimeAfterdose(dat_dose,dat_sample)
        max_time <- max(c(dos_dat$time,obs_dat$time))


        weight <- input$weight
        crcl <- input$crcl


        par_ind <- MAP.est(init_eta, obs_dat, dos_dat, mod,
                        weight, crcl, POPpar, OMEGA, SIGMA)
        # Sample times: when a concentration was collected
        sim_sample_times <- seq(from=0, to=max_time+36, by=0.1)
        # Define event record
        ev <- et(time = dos_dat$time,
                 amt  = dos_dat$dose,
                 evid = 1, # dosing records
                 dur = dos_dat$dur,
                 cmt = dos_dat$cmt) %>%
```

```
        add.sampling(sim_sample_times)

      #----------------- Concentration-time profile simulation -----
-------#
      # Simulation for individual
      sim_ind_data <-  rxSolve(mod, params = par_ind, events = ev)


#-------------------- PD endpoint calculation -----------------------
# ####
      # AUC
      auc_ind <- auc_ind(data=sim_ind_data)

      res_text <- paste0("The AUC0-24h calculated by MAP-BE method
is ", round(auc_ind,2)," mg*h/L.")

      # plot individual prediction
      plotobj <- simPlot(sim_ind_data = sim_ind_data, raw_dat =
obs_dat)

      # return results
      return(list(res_text = res_text,
                  plotobj = plotobj))
   })

   #-----------------------------------------------------------------
------------------------
   # output

   ## indicator for the first click of action button
   click <- reactiveValues(data = NULL)
   observeEvent(input$submit, {
      click$data <- 1
   })

   ## display a plot with original data and simulation result
   output$plotConc <- renderPlot({
      if (is.null(click$data)) {
         # default plot when open the website: default case
         print(simPlot(sim_ind_data = df_ind_data,raw_dat =
df.sample))
      }else{
         # update plot by clicking action button
         print(res()$plotobj)
```

```
        }
    }, height = function(){
        if(session$clientData$output_plotConc_width > 400) {
            return(400)
        }else{
            return(0.9*session$clientData$output_plotConc_width)
        }
    })  #Brackets closing "renderPlot" function

    # Report AUC
    output$res_text <- renderText({
        if (is.null(click$data)) {
            # default table when open the website:
            "The AUC0-24h calculated by MAP-BE method is 60.15 mg*h/L."
        }else{
            # update table by clicking action button
            res()$res_text
        }
    })  #Brackets closing "renderText" function
    #############
    ##_SESSION_##
    #############
    # Close the R session when Chrome closes
    session$onSessionEnded(function() {
        stopApp()
    })

})  #shinyServer
```

Define UI of Shiny app

```
# Define UI
fixedPage(
    # Application Title and Logo
    fixedRow(
        column(12,
               h3("AUC0-24h Calculator of valganciclovir and
ganciclovir"),
               align = "center")
    ),  #Brackets closing "fixedRow"
    # Add a break with a horizontal line
    # Sidebar panel with widgets
    sidebarLayout(
        sidebarPanel(
            h4("Patient information:"),
            # input weight
            numericInput('weight', label = h4("Weight (kg):"), 8, min =
0, max = 200),
            # input scr
            numericInput('crcl', label = h4("CrCL (mL/min/1.73 m2):"),
96, min = 0, max = 500),

            h4("Dose record (mg)"),
            rHandsontableOutput("table1"),
            # add a row to table
            actionLink("addPoint","Add"),
            # drop a row from table
            actionLink("dropPoint","Drop"),

            # Sample concentration record table
            h4("Sample record (mg/L)"),
            rHandsontableOutput("table2"),
            # add a row to table
            actionLink("addLink","Add"),
            # drop a row from table
            actionLink("dropLink","Drop"),
            br(),
            # actionButton
            actionButton("submit","Calculate",
                         icon = icon("refresh")), #actionButton

            align = "left",
            width = 5
```

```
        ), #sidebarPanel

        # Main panel to contain help notes and result
        mainPanel(
            width = 7,
            # Plot output for concentration-time profile
            fixedRow(
                plotOutput("plotConc",width="95%")
            ),
            hr(),
            h4("The result of AUC:",align="left"),
            verbatimTextOutput("res_text"),
            align = "center",
            hr(),
            h6("The PopPK model we used in this AUC calcultor was
developed by Franck et al.",
               align="right"),
            h6("For more information on the PopPK model, please look at
the following publication:",align="right"),
            h6(span("https://pubmed.ncbi.nlm.nih.gov/33373493/", style
= "color:blue"), align="right"),
            tags$a(href="https://pubmed.ncbi.nlm.nih.gov/33373493/",
target="_blank",
                   h5("Click here!",align="right"))
        )    #mainPanel
    ), #sidebarLayout
    )   #fixedPage
```

Validation of the $AUC_{0\text{-}24h}$ calculator with NONMEM

To prove the reliability of our AUC calculator, we compared AUC calculator's results with NONMEM's results in 3 different virtual patients.

1. Virtual patients' demographics

ID	1	2	3
Weight (kg)	15	100	5
CrCL (mL/min/1.73m^2)	90	250	35

2. Dosing records

	1			2			3		
Time (h)	0	11.5	23.5	0	11.5	23.5	0	11.5	23.5
Dose (mg)	75	75	75	800	1000	1000	30	50	50
Intravenous infusion (Y/N)	Y	Y	Y	N	N	N	Y	N	N
Infusion during (h)	1	1	1	0	0	0	1	0	0

3. Sampling records

	1		2		3	
Time (h)	23	25	23	25	23	25
Concentration (mg/L)	0.8	3.2	1	3.8	0.6	3

4. Estimates of individual PK parameters

	1		2		3	
	AUC calculator	NONMEM	AUC calculator	NONMEM	AUC calculator	NONMEM
CL	3.953227	3.9532	23.5186454	23.51800	0.7082963	0.70829
Vc	11.112090	11.1120	40.5690043	40.56900	1.8278532	1.82790
Q	10.900000	10.9000	10.900000	10.9000	10.900000	10.9000
Vp	4.269663	4.2697	28.4644195	28.46400	1.4232210	1.42320
Tlag	0.330000	0.3300	0.330000	0.3300	0.330000	0.3300
Ka	0.730000	0.7300	0.6456028	0.64560	0.6596735	0.65967
F	0.430000	0.4300	0.4518597	0.45186	0.3590508	0.35905

5. Results of $AUC_{0\text{-}24h}$

	1		2		3	
	AUC calculator	NONMEM	AUC calculator	NONMEM	AUC calculator	NONMEM
$AUC_{0\text{-}24h}$	37.67698	37.67698	33.51024	33.51049	64.80461	64.80505

So, the calculation result of $AUC_{0\text{-}24h}$ calculator is reliable.
